# Ancient DNA Reveals Matrilineal Continuity in Present-Day Poland over the Last Two Millennia

**DOI:** 10.1371/journal.pone.0110839

**Published:** 2014-10-22

**Authors:** Anna Juras, Miroslawa Dabert, Alena Kushniarevich, Helena Malmström, Maanasa Raghavan, Jakub Z. Kosicki, Ene Metspalu, Eske Willerslev, Janusz Piontek

**Affiliations:** 1 Department of Human Evolutionary Biology, Faculty of Biology, Adam Mickiewicz University in Poznan, Poznan, Poland; 2 Molecular Biology Techniques Laboratory, Faculty of Biology, Adam Mickiewicz University in Poznan, Poznan, Poland; 3 Evolutionary Biology Group, Estonian Biocentre, Tartu, Estonia; 4 Centre for GeoGenetics, Natural History Museum of Denmark, University of Copenhagen, Copenhagen, Denmark; 5 Department of Evolutionary Biology, Uppsala University, Uppsala, Sweden; 6 Institute of Environmental Biology, Faculty of Biology, Adam Mickiewicz University in Poznan, Poznan, Poland; 7 Department of Evolutionary Biology, University of Tartu, Tartu, Estonia; University of Florence, Italy

## Abstract

While numerous ancient human DNA datasets from across Europe have been published till date, modern-day Poland in particular, remains uninvestigated. Besides application in the reconstruction of continent-wide human history, data from this region would also contribute towards our understanding of the history of the Slavs, whose origin is hypothesized to be in East or Central Europe. Here, we present the first population-scale ancient human DNA study from the region of modern-day Poland by establishing mitochondrial DNA profiles for 23 samples dated to 200 BC – 500 AD (Roman Iron Age) and for 20 samples dated to 1000–1400 AD (Medieval Age). Our results show that mitochondrial DNA sequences from both periods belong to haplogroups that are characteristic of contemporary West Eurasia. Haplotype sharing analysis indicates that majority of the ancient haplotypes are widespread in some modern Europeans, including Poles. Notably, the Roman Iron Age samples share more rare haplotypes with Central and Northeast Europeans, whereas the Medieval Age samples share more rare haplotypes with East-Central and South-East Europeans, primarily Slavic populations. Our data demonstrates genetic continuity of certain matrilineages (H5a1 and N1a1a2) in the area of present-day Poland from at least the Roman Iron Age until present. As such, the maternal gene pool of present-day Poles, Czechs and Slovaks, categorized as Western Slavs, is likely to have descended from inhabitants of East-Central Europe during the Roman Iron Age.

## Introduction

Continuity of human occupation in the territory of Central Europe, modern-day Poland in particular, and its relation to the origins of the Slavs have been widely discussed in the archaeological, linguistic and historical literature; however, these questions still remain contentious [1]–[5]. At present, vast territories of East-Central and South-East Europe are inhabited by Slavic populations [5]. Three groups of present-day Slavs are identified on the basis of their linguistic affinities: Western Slavs (Poles, Czechs and Slovaks), Eastern Slavs (Ukrainians, Belarusians and Russians) and Southern Slavs (Croatians, Bulgarians, Slovenians, Bosnians, Macedonians, Montenegrins and Serbians) [6]. It is supposed that all Slavs, besides their linguistic affinity, also share a common place of origin, although the latter is still inconclusive [5].

Several hypotheses have been advanced regarding the origin and early migrations of Slavs, of which two - *autochthonous* and *allochthonous* - have predominated. According to the *autochthonous* hypothesis, territories around Oder and Vistula rivers (in present-day Poland) were continuously inhabited by ancestors of Slavs from the Roman Iron Age (0**–**400 AD), or perhaps even further back in time from the Bronze Age (3200**–**600 BC) [7] until the Medieval Age (500**–**1500 AD) [8]. In contrast, the *allochthonous* theory suggests the discontinuity of settlements between Roman Iron Age and Medieval Age in the territory of present-day Poland. Allochthonists hypothesize that the Slavs originated in the Pripet and Middle Dnieper River basins in modern-day Ukraine, from where they migrated to the west and south of Europe in the beginning of 5^th^ century AD and inhabited the lands of present-day Poland, which was previously occupied by Germanic tribes during the Roman Iron Age [9]. However, morphological analyses of skeletal materials from present-day Poland have suggested a continuity between Roman Iron Age (represented by Przeworsk and Wielbark cultures) and Medieval Age populations [10], [11] thus providing less support to the *allochthonous* model.

Genetic studies on present-day Slavic-speaking populations have also addressed the complex genetic history of the Slavs [12]–[15]. The comparison of the complete mitochondrial genome sequences revealed a number of lineages that seem specific for Central and Eastern Europe. Moreover, based on age estimations, the authors suggest a genetic continuity of some Slavic mitochondrial lineages from at least the Bronze Age [15].

Ancient DNA (aDNA) provides direct genetic evidence for past demographic events. Mitochondrial DNA (mtDNA) from skeletal remains has been particularly successful in reconstructing the evolutionary history of European populations (e.g. [16]–[21]). However, no large-scale aDNA study on putative ancestral populations of modern-day Slavs have been reported thus far. Ancient DNA datasets from regions geographically adjacent to present-day Poland are limited to Iron and Middle Age samples from Denmark [22]–[25], Neolithic samples e.g. from the *Linearbandkeramik* culture from Germany [26] and Bronze Age samples from Ukraine, Bulgaria and Moldova [27].

Therefore, to provide fresh perspectives on the debate of genetic continuity in Central Europe during the last two millennia, and to contribute to the resolution of the complex origin of the Slavs, we present the first population-level ancient mtDNA analysis on samples originating from six archaeological sites in Poland. The studied samples date to the Roman Iron Age, represented by Wielbark and Przeworsk cultures and to the Medieval Age. Our aim is to determine matrilineal genetic structure of ancient populations in the area comprising contemporary Poland, their relationships to one other and to other ancient and modern human populations from Europe, and to investigate potential genetic continuity between populations spanning two millennia.

## Materials and Methods

### Archaeological sites and samples

**Figure 1:**
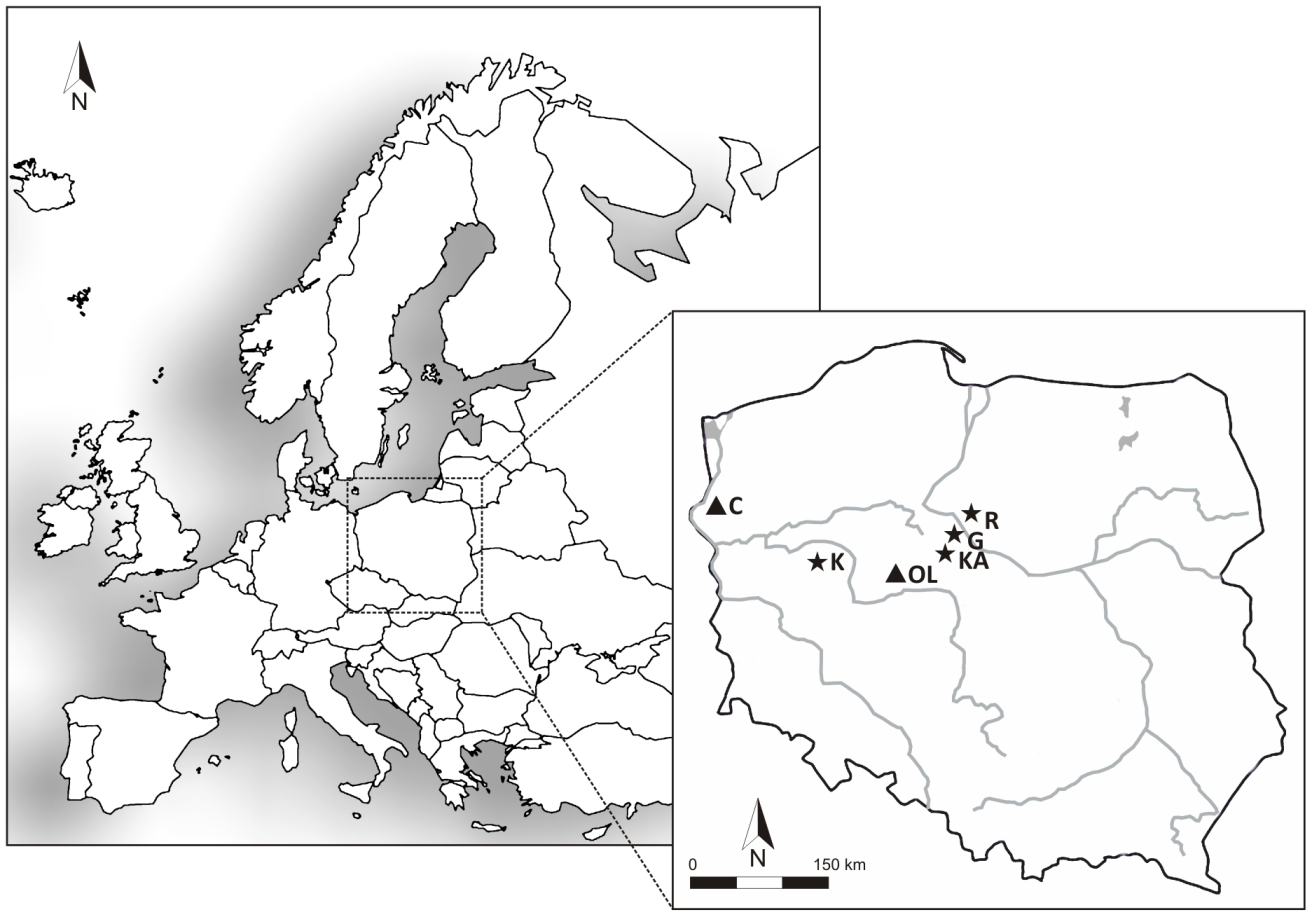
Locations of the Roman Iron Age (RoIA) and Medieval Age (ME) burial sites in the territory of present-day Poland. RoIA (stars): Kowalewko (K), Karczyn (KA), Gąski (G), Rogowo (R). ME (triangles): Cedynia (C), Ostrów Lednicki (OL).

The skeletal material studied here originated from burial sites located in present-day Poland dating to the Roman Iron Age and the Medieval Age (n = 72) (Figure 1). The Roman Iron Age samples (RoIA) comprised 38 human remains from cemeteries in Kowalewko (n = 11) and Rogowo (n = 13) assigned to the Wielbark culture, and two burial sites in Karczyn (n = 12) and Gąski (n = 2) assigned to the Przeworsk culture. The Wielbark culture extended in the north-eastern territories of contemporary Poland during the 1^st^ to the 4^th^ century AD. The Przeworsk culture was present in the Western, Central and Southern Poland from the 3^rd^ century BC to the 5^th^ century AD. Both Wielbark and Przeworsk cultures were dated to the Roman Iron Age based on the archaeological context. Medieval Age (ME) samples comprised 34 human remains recovered from cemeteries in Cedynia (n = 18) and Ostrów Lednicki (n = 16). Detailed information about each sample, the archaeological context of the burial sites and their geographic origins is presented in Table 1 and Table S1. The handing history of samples is not well recognized; however seems to be minimal due to obtained results since aDNA was obtained only from intact teeth that are thought to be less prone to modern human DNA contaminations than other skeletal parts [28]. The permission for collecting samples for aDNA studies from all mentioned above archaeological sites was provided by the supervisor of the skeletal materials, Head of the Institute of Anthropology and Department of Human Evolutionary Biology at Adam Mickiewicz University in Poznan.

**Table 1:** Geographical origins and archaeological contexts of ancient samples analyzed in the present study.

BURIAL SITES	N	SAMPLE NAMES	CONTEXT
Kowalewko (K)	11	K1-K11	RoIA (100-300 AD; Wielbark culture)
Rogowo (R)	13	R1-R13	RoIA (200 AD; Wielbark culture)
Karczyn (KA)	12	KA1-KA12	RoIA (200-500 AD; Przeworsk culture)
Gąski (G)	2	G1-G2	RoIA (200 BC-100 AD; Przeworsk culture)
Cedynia (C)	18	C1-C18	ME (1000-1400 AD)
Ostrów Lednicki (OL)	16	OL1-OL16	ME (1100-1400 AD)

N represents the number of analyzed individuals from each of the sites.

### Extraction of ancient DNA

Sampling was performed using disposable gloves, facemasks and body suits to minimize the risk of contamination from modern humans. Two teeth were collected from each individual. All pre-PCR work was conducted in laboratories dedicated exclusively to the analysis of low copy number DNA (Centre for GeoGenetics at the University of Copenhagen, Denmark and Ancient DNA Laboratory at the Adam Mickiewicz University in Poznan, Poland). The outer surface of the teeth was decontaminated using 0.1 M HCl, followed by drilling of the teeth and treatment of the powder with 0.5% NaOCl [29]. DNA was extracted using a silica-column based method [29], [30] which was modified by the addition of urea to the extraction buffer [31]. DNA extractions from the two teeth from each individual were performed at separate times. Preparation of reagents and solutions was conducted under sterile conditions and appropriate precautions (e.g. UV irradiation) were taken to avoid modern DNA contamination. Negative controls were set up during extractions (one control for every four samples) and amplifications (one control for every eight PCR reactions). Three faunal samples, contemporary with the human skeletal samples, were retrieved from one of the locations (Kowalewko) and were used as controls to screen for contamination from modern human sources both during and post-excavation.

### Mitochondrial DNA analysis

Seven sets of overlapping primer pairs (Table S2) were used to amplify 360 base pairs (bp) of the first hypervariable region (HVRI) of the mtDNA control region between nucleotide positions (nps) 16043-16403, according to the revised Cambridge Reference Sequence (rCRS) (NC_012920.1) [32]. Haplogroup-diagnostic nps in HVRII and mtDNA coding region were amplified using eighteen primer pairs, respectively, and HVRI regions between nps 16043-16132 and 16307-16403 were amplified using M13-tailed primers (Table S2). PCR reactions were set up as follows: 2 µl DNA extract, 0.5 U Platinum Taq DNA Polymerase High Fidelity (Invitrogen), 1X High-Fidelity PCR Buffer, 2 mM MgSO_4_, 0.8 mg/ml RSA (Calbiochem), 200 µM each of dNTPs (Invitrogen), 500 nM of each primer and ddH_2_O up to 25 µl. The thermocycling conditions were as follows: initial denaturation at 94°C for 4 minutes; 42 cycles of 94°C for 30 seconds, 52°C**–**60°C depending on the primer pair, (Table S2) for 20 seconds, 68°C for 20 seconds; and final extension at 72°C for 10 minutes. PCR products were visualized through electrophoresis on 2% agarose gel.

Almost all analyzed fragments, including HVRI, HVR-II and coding region fragments containing diagnostic SNPs were cloned and sequenced. Only fragments comprising nps 16048-16132 and 16307-16403 were amplified with M13-tailed primers and directly sequenced without cloning. Amplicons were cloned into pCR2.1-TOPO vector and transformed into competent *E. coli* cells (One Shot *E. coli*) using TOPO TA cloning kit (Invitrogen), following the supplier's instructions. More than twelve bacterial colonies from each cloning experiment were screened for inserts using M13 universal primers. At least four positive clones were sequenced for each case. PCR products were purified with exonuclease I and Fast alkaline phosphatase (Fermentas) and sequenced using BigDye Terminator v3.1 kit and ABI Prism 3130xl Genetic Analyzer (Applied Biosystems), following manufacturer's instructions. Amplicons generated with M13-tailed primers were sequenced in both directions using M13 universal primers.

In order to determine consensus sequence and detect *post-mortem* damages or/and possible contaminations, alignment of mtDNA sequences was performed using BioEdit v.7.0.5.3 (http://www.mbio.ncsu.edu/BioEdit/bioedit.html). Polymorphic positions in HVRI, HVRII and coding region sequences were scored against the rCRS sequence. Haplogroups were assigned following the established hierarchy of mtDNA phylogeny [33].

### Populations used in comparative analyses

In order to compare mtDNA profiles of RoIA and ME samples to modern populations, we compiled a database of published mtDNA diversity in contemporary Poles, Czechs, Slovaks, Belarusians, Ukrainians, Russians (from the European part), Slovenians, Bosnians, Croatians, Bulgarians, Serbs, Germans, Finns, Estonians, Latvians, Lithuanians, Macedonians and Swedes (Table S3). To determine genetic distances between ancient populations, we also compiled a dataset of published mtDNA diversity in populations from Iron Age, Early Christian period and Middle Age from present-day Denmark (Germanic tribes), and Neolithic population from Linear Pottery Culture (LBK) from Germany (Table S3).

For haplotype sharing analysis we considered sequences bounded by np 16043 to 16403 of the HVRI for both aDNA samples in this study and those from modern populations of our database. All matches between haplotypes were scored taking into account their haplogroup affiliation. Because different studies have used different phylogenetic resolution of mtDNA, for haplotype sharing analysis and for MDS we considered the phylogenetic resolution depth reached in our aDNA samples.

### Haplotype sharing analysis

Haplotype sharing analysis was conducted in order to detect mtDNA haplotypes shared between RoIA and ME samples and modern Europeans. To this end, eighteen contemporary populations representing East-Central, Southeast and North Europe were used in the analysis (Table S3). Several populations (Slovaks/Czechs, Macedonians/Serbs, Bosnians/Slovenians/Croatians, Finns/Estonians and Lithuanians/Latvians) were pooled together so that the total sample size (N) for each population amounted to approximately 300 individuals (between 277 and 317). Pooling was performed according to the linguistic affinities and geographic location of populations. Likewise, the sample sizes for populations with N>300 samples were decreased by sampling 300 randomly selected individuals. Consequently, a total of twelve populations or groups of pooled populations were used in the analysis.

The presence or absence of a particular haplotype in a given contemporary population was marked as „1” or „0”, respectively, and the total number as well as frequency of ancient haplotypes found in each modern population was calculated. Furthermore, all haplotypes present in RoIA and ME samples were divided into three classes based on their incidence in the comparative modern populations: informative haplotypes, which were found in less than half of the comparative populations; non-informative haplotypes, which occurred in more than half of the comparative populations; and unique haplotypes, which did not have exact matches in contemporary populations. In cases where we reduced sample sizes, we have cross-checked for informative/unique haplotypes if they present in whole samples size.

We used the two-tailed z-test to assess statistical significance of shared informative haplotypes between ancient and modern populations [26]. Nonparametric bootstrapping of 1000 replicates for each population was used to generate the confidence intervals for the percentage of all matches, informative matches, and non-informative matches. The analysis was performed in R (R Development Core Team 2013) [34] using boot library [35].

### Population pairwise F_ST_

MtDNA haplogroup frequencies were used to determine genetic distances between ancient and modern populations. MtDNA haplogroups of comparative modern Slavic groups as well as Iron Age and Middle Age populations from present-day Denmark [22]–[25] and Neolithic population (LBK) from present-day Germany [26] were used in this analysis. Slatkin's linearized pairwise *F*_ST_ were calculated using Arlequin v.3.5 [36]. Multidimensional Scaling (MDS) plot was built based on population pairwise *F_ST_* values using Statistica v.10 StatSoft (2011).

## Results

### Authenticity of ancient DNA results

Reproducible mtDNA sequences were obtained from 23 out of the 38 specimens from Roman Iron Age burial sites (Table 2), and from 20 out of the 34 samples from the Medieval Age sites (Table 3). The consensus nucleotide sequences were supported by an alignment of at least 10 clones deriving from partially overlapping amplicons obtained from at least two independent DNA extractions (nps 16050-16130, 16119-16196, 16181-16226 and 16209-16356) (Figure S1). Therefore, if the same polymorphic positions were detected in cloned sequences retrieved from two teeth of each individual, the results were considered to be authentic. In four individuals we did not obtain a fragment 16209-16356 from one of the two DNA samples extracted from the same individual. Thus a shorter fragment of HVRI (np 16249-16317) was amplified, cloned and sequenced. For eleven individuals a fragment comprising nps 16050-16130 was obtained. For samples which had been confirmed by cloning as containing aDNA free from contaminations of modern human DNA a direct sequencing of M13-tailed amplicons comprising nps 16048-16132 and 16307-16403 was performed. Twenty nine out of 72 samples failed during the PCR amplifications or showed inconsistent alignment of cloned sequences and were discarded from the analyses.

**Table 2:** MtDNA haplogroups (hg) identified in Roman Iron Age populations.

SAMPLE NAMES	HVRI REGION (16043-16403)	CR SNPs	Hg
R1	rCRS	7028C	H
R2	16093C, 16129A, 16316G	7028C	H
R3	16153A, 16304C	7028C, 15833T	H5a1
R4	16362C	7028C	H
R5	16183C, 16189C, 16356C	7028C	H
R6	rCRS	7028C	H
R7	rCRS	7028C	H
R8	16093C	7028C	H
R9	16183C, 16189C, 16356C	7028C	H
R10	16129A	7028C	H
R11	16069T, 16126C, 16145A, 16231C, 16261T, 16299G	10398G	J2a
R12	16126C, 16294T, 16296T, 16304C		T2
K1	16304C	7028C, 15833T	H5a1
K2	rCRS	7028C	H
K3	16223T, 16292T	8251A	W
K4	16223T, 16292T	8251A	W
K5	16192T, 16223T, 16292T, 16399G	8251G	W
K6	16192T, 16270T	7768G	U5b
K7	16343G, 16390A	14139G	U3
KA1	16222T	7028C	H
KA2	16147A, 16223T, 16248T, 16320T, 16355T	10238C	N1a1a2
G1	16129A, 16304C	7028C, 15833T	H5a1
G2	16256T, 16263C, 16270T, 16399G	15218G, 3816A	U5a1

rCRS refers to the revised Cambridge Reference Sequence and CR refers to the mtDNA coding region.

**Table 3:** MtDNA haplogroups identified in Medieval populations.

SAMPLE NAMES	HVRI REGION (16043-16403)	CR SNPs	Hg
C1	16111T	7028C	H
C2	16362C	7028C	H
C3	16354T	7028C	H
C4	16080G, 16189C, 16356C	7028C	H
C5	16126C	7028C	H
C6	16162G	7028C	H1a
C7	16224C, 16311C	1189C	K1
C8	16222T, 16224T, 16270T, 16311C	146C, 152T (HVRII), 1189T	K2
C9	16183C,16189C, 16223T, 16258C, 16266T, 16274A, 16278T, 16390A	146C, 195C (HVRII), 1719G	X4
C10	16183C,16189C, 16223T, 16278T	146T, 195C (HVRII), 1719A	X2
C11	16189C, 16271C	7028T, 14766T	HV
C12	16069T, 16126C, 16145A, 16222T, 16261T		J1b
C13	16126C, 16362C		R0a
C14	16129A, 16145A, 16298C	7028T, 72C (HVRII)	HV0
C15	16145A, 16176G, 16209C, 16223T, 16390A		N1b
OL1	16304C	7028C, 15833T	H5a1
OL2	16311C	7028C	H
OL3	16126C,16163G, 16186T, 16189C, 16294T		T1a
OL4	16069T, 16126C, 16145A, 16172C, 16222T, 16261T	10398G	J1b
OL5	16223T	8251A	W

rCRS refers to the revised Cambridge Reference Sequence and CR refers to the mtDNA coding region.

All cloned mtDNA inserts showed characteristic *post-mortem* ancient DNA damages, of which 99% wereby C>T and G>A transitions [37]–[39]. Sequences obtained with the same procedure in the two independent laboratories were consistent. No evidence of modern human DNA contamination was present in the faunal material used as negative controls.

### MtDNA haplogroup composition in ancient populations

All haplotypes identified in RoIA and ME populations belonged to typical West Eurasian mtDNA haplogroups (hg): H, K, U, T, J, W, HV, X, HV0, R0, and N (Table 2 and 3). There were 18 different mtDNA haplotypes found among 23 RoIA individuals and 20 distinct mtDNA haplotypes identified among 20 ME samples.

Haplogroup (hg) H was the most abundant hg in the RoIA populations (60.9% of all genotyped RoIA individuals). Samples from Rogowo comprised the highest number of individuals (10 out of 12) assigned to hg H, of which three displayed HVRI sequence identical to the rCRS. Three haplotypes, originating from Kowalewko and Gąski burial sites, were assigned to three sub-branches of hg U (U5a, U5b and U3) (Table 2). Haplogroup W was only identified in three individuals from the Kowalewko site. Remaining hgs (T, J2a and N1a) were identified in one individual each.

Haplogroup (hg) H was also the most frequent hg among the ME samples (40% of all analyzed ME individuals) (Table 3). Other frequent hgs in the ME population were K, J and X, each occurring in two specimens. Haplogroups T, W, N1b, HV, HVO, and R0a were only observed in one individual each.

### MtDNA haplotypes shared between ancient and modern populations

A set of 3595 modern mitochondrial haplotypes, including majority of Slavic populations from regions geographically adjacent to Poland, was used for comparative analysis. Haplotypes shared between ancient and modern populations are presented in Table S4.

**Figure 2:**
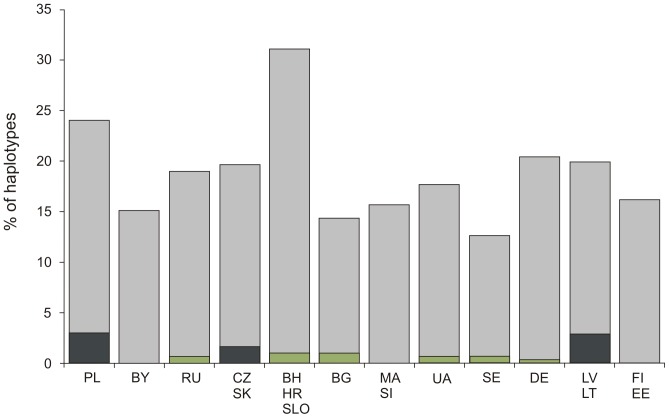
Frequencies (%) of haplotypes shared between Roman Iron Age individuals and sampled modern Europeans. Grey bar- all shared haplotypes; green - informative haplotypes, which not differ significantly from the average frequency (p<0.05); graphite - informative haplotypes, which differ significantly from the average frequency (p<0.05). Abbreviations for populations: Poles (PL), Belarusians (BY), Russians (European part) (RU), Czechs/Slovaks (CZ, SK), Bosnians/Slovenians/Croatians (BH, SLO, HR), Bulgarians (BG), Macedonians/Serbs (MA, SI), Ukrainians (UA), Swedes (SE), Germans (DE), Lithuanians/Latvians (LT, LV), Estonians (EE), Finns (FI).

Among haplotypes identified in the RoIA populations, seven were found to be frequent in most of the modern-day populations in our database and thus classified as non-informative. These widespread haplotypes belong to common West Eurasian hgs H, T and W. The 16223-16292 (hg W) haplotype represents a basal haplotype within the phylogeny of hg W. Eight RoIA haplotypes were infrequent in contemporary populations and were classified as informative (Table S4). The relative frequencies of the shared informative and non-informative RoIA haplotypes were calculated in each of twelve present-day populations. Three modern populations or groups of populations (Lithuanians and Latvians, Poles, and Czechs and Slovaks) were found to contain significantly higher percentages (*p*<0.05) of shared informative haplotypes with the RoIA samples compared to other present-day populations (Figure 2, Table S4). Notably, modern Poles shared the highest number (nine) of informative mtDNA haplotypes with the RoIA individuals. The remaining three haplotypes had no match in the screened modern populations and were classified as unique (Table S3). These unique haplotypes belonged to mtDNA hgs N1a (16147A-16223-16248-16320-16355), with (likely) back mutation (C>T) at the position 16172, U5a (16256-16263-16270) and W (16192-16223-16292-16399).

**Figure 3:**
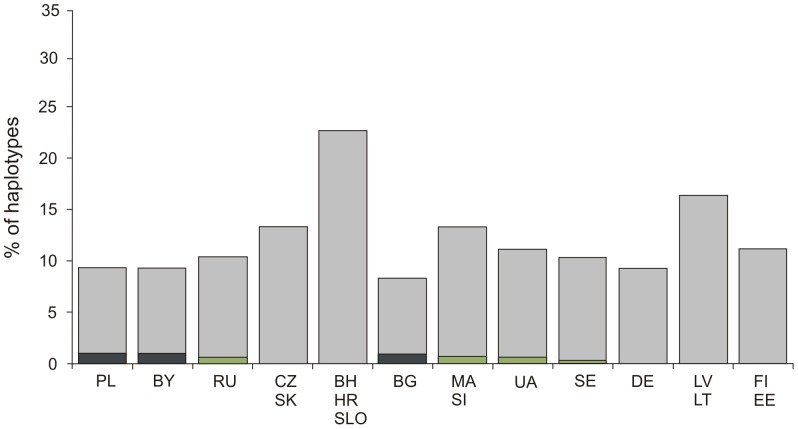
Frequencies (%) of haplotypes (matches) shared between Medieval Age individuals and sampled modern Europeans. Grey bar - all shared haplotypes; green bar- informative haplotypes, which not differ significantly from the average frequency; graphite bar - informative haplotypes, which differ significantly from the average frequency (p<0.05). For population abbreviations, see Figure 2.

Among distinct mtDNA haplotypes in the ME samples, eight belonging to hgs H, T, K, and J were present in high frequencies in modern-day populations and were hence classified as non-informative (Table S4). The informative group consisted of nine haplotypes occurring in low frequencies in six modern populations (Table S4); however, only three out of the 12 tested modern populations (Bulgarians, Poles and Belarusians) were found to share significantly higher percentage (*p*<0.05) of informative haplotypes with the ME samples (Figure 3). Three haplotypes, characteristic of hg X4 (16183-16189-16223-16258-16266-16274-16278), hg HV0 (16129-16145-16298) and hg HV (16189-16271) did not have matches to the studied modern populations and were thus considered as unique to the ME samples.

### MtDNA haplotypes shared between RoIA and ME populations

Comparison of mtDNA compositions of the RoIA and ME populations revealed two shared haplotypes. The first one was assigned to hg H with a mutation at position 16362. The second one belonged to hg H5 with a mutation at the diagnostic position 16304. Of the analyzed individuals assigned to hg H5, three RoIA haplotypes: 16153-16304 (sample R3), 16304 (sample K1), 16129-16304 (sample G1) (Table 2), and one ME haplotype (16304 (sample OL1)) (Table 3), further belonged to subhaplogroup H5a1 with defining mutation at coding region position 15833.

### Pairwise distances and MDS

**Figure 4:**
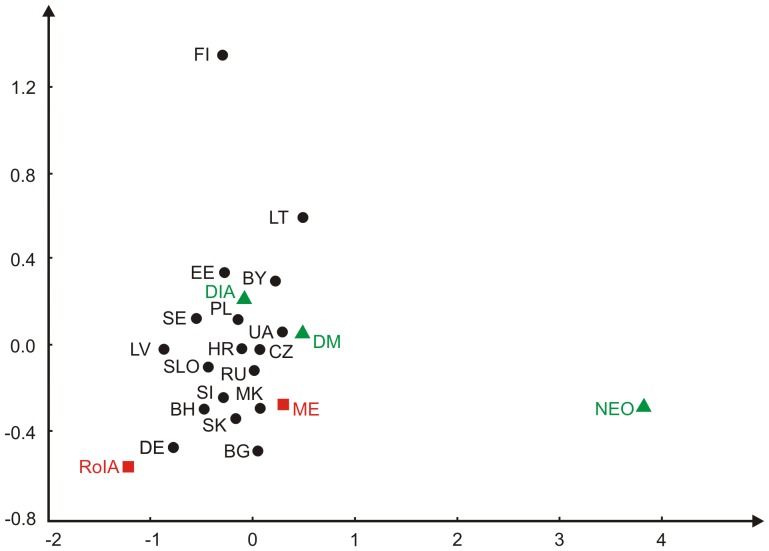
Multidimensional Scaling plot based on F*_ST_* values calculated from mitochondrial haplogroup frequencies in sampled European populations. Modern Slavic populations and other populations adjacent to Poles (black): for populations abbreviations, see Figure 2; ancient comparative populations (green): Danish (Iron Age) (DIA), Danish (Medieval) (DM), Neolithic (LBK, Germany) (NEO); present study populations (red): Roman Iron Age (RoIA), Medieval Age (ME).

Pairwise genetic distances were calculated in order to reconstruct the genetic relationship between the ancient and modern populations. Pairwise *F_ST_* values showed non-significant differences between the RoIA and the ME samples (p>0.01) (Table S5). The RoIA samples differed significantly when compared to Neolithic individuals (LBK, Germany), Ukrainians, Belarusians, Latvians and Finns (p<0.01). The ME individuals showed no significant genetic differences to other populations used in the analysis (p>0.01), with the exception of the Neolithic (LBK) population and Finns (p<0.01) (Table S5). Correspondingly, in resulted MDS plot both ancient samples from this study (RoIA, ME) are mostly differentiated from northeast modern Europeans and Neolithic LBK sample, while being within the conglomerate of the remaining populations (Figure 4).

## Discussion

This is the first large-scale study presenting mtDNA profiles for 43 individuals recovered from Roman Iron Age and Medieval Age burial sites from modern-day Poland. All mtDNA hgs identified in RoIA and ME populations are observed in most modern Slavic populations [40]–[51]. However, distinct haplotypes detected in the ancient samples and corresponding frequencies in modern populations have the potential to address the question of continuity of mitochondrial lineages over time in the area of present-day Poland.

Haplotype sharing analysis indicates that the RoIA individuals shared the highest number of informative mtDNA haplotypes with present day Poles (Table S4). Of particular interest are three RoIA samples assigned to subhaplogroup H5a1, which were recovered from the Kowalewko (sample K1), the Gąski, and the Rogowo (samples G1 and R3) burial sites (see Figure 1). Recent studies on mtDNA hg H5 have revealed that phylogenetically older subbranches, H5a3, H5a4 and H5e, are observed primarily in modern populations from southern Europe, while the younger ones, including H5a1 that was found among RoIA individuals in our study, date to around 4.000 years ago (kya) and are found predominantly among Slavic populations of Central and East Europe, including contemporary Poles [15]. Notably, we also found one ME sample belonging to subhaplogroup H5a1 (sample OL1 in Table 3). The presence of subclusters of H5a1 in four ancient samples belonging to both the RoIA and the ME periods, and in contemporary Poles, indicates the genetic continuity of this maternal lineage in the territory of modern-day Poland from at least Roman Iron Age i.e. ∼ 2 kya. Age estimates for other subhaplogroups of mtDNA hg H5 (Ha5a2, H5e1a and H5u1) as well as for U5a2b1, U5a2a and U4a are similar as for subhaplogroup H5a1 (∼4 kya) in Central and Eastern Europe [15], [42].

The evolutionary age of H5 sub-branches (∼4 kya) [15] also approximates the age of N1a1a2 subclade found in the RoIA population (sample KA2) (Table 2). The coalescence age of N1a1a2 is around 3.4**–**4 kya, making this haplotype one of the youngest sub-branches within hg N [52]. The N1a1a2 haplotype found in one RoIA individual was classified as unique because no exact match was found among the twelve comparative populations or groups of populations used in the haplotype sharing test. Notably, a similar N1a1a2 haplotype carrying an additional transition at position 16172 was found in a modern-day Polish individual [53]. Taken together, the presence of mtDNA subhaplogroups N1a1a2 and H5a1 in both the ancient populations as well as in studied modern Poles suggests a genetic continuity of certain matrilineages in the territory of present-day Poland, at least from the Roman Iron Age.

Haplotype sharing test revealed that RoIA populations share significantly higher number of haplotypes not only with present-day Poles, and Czechs and Slovaks (Slavic populations), but also with Lithuanians and Latvians (Table S4). Similarity in genetic compositions among ancient populations across Central and north-eastern Europeans may reflect their shared deep matrilineal history being consistent with demographic history of Europe in general [54]. Additionally, more recent events such as migration and admixture might have also contributed to the observed genetic similarity. For instance, archaeological evidence and historical records show extensive cultural connections between populations over wide areas of Europe during the Iron Age [55]. In particular, the Roman Empire extended throughout the latitudinal breadth of Europe in the Iron Age. During this period, trade routes crossed the territories of modern Poland and eastern regions, inhabited by Baltic tribes [55] thus enabling contacts between RoIA populations (in particular those belonging to Wielbark culture) and neighboring Baltic groups, resulting not only in cultural exchange but, possibly, also in gene flow.

The ME populations share significantly higher percentages of haplotypes with modern Poles, Belarusians and Bulgarians (p<0.05) (Table S4). Shared haplotypes over time might reflect genetic continuity between populations from RoIA, ME and modern Poles as well as a matrilineal gene flow towards east and south of Europe at the onset of the Middle Ages, coinciding with movements of Slavic groups [56].

Inclusion of ancient Danish samples for comparative analysis was prompted by the fact that these groups as well as those from the other parts of Scandinavia are thought to have descended from Germanic tribes that occupied the territory of modern-day Poland during the Roman Iron Age [9]. The MDS plot showed large genetic differentiation between Iron Age individuals from Denmark and RoIA individuals from Poland (Figure 4), although the *F_ST_* values were not statistically significant (p>0.01). Both populations shared haplotypes belonging to subhaplogroups U3a and T2. Notably, we did not identify mtDNA hg I, occurring at high frequency (13%) in ancient Iron and Middle Age Danish populations [23], among any of the 43 successfully amplified ancient individuals from Poland.

We note that due to the uniparental inheritance of the mitochondrial genomes and relatively small sample sizes in our study, our observation of the various mtDNA hgs in the ancient populations, both the presence/absence and frequencies, are likely to be prone to stochastic events. Hence, future research including nuclear DNA markers are necessary to further explore the genetic connections between these ancient and modern populations, including the question of a common origin and the extent of admixture, if any, between them.

## Conclusions

Results of our study indicate genetic continuity of mitochondrial lineages between ancient and modern populations in the territory of contemporary Poland. In particular, presence of sub-clades of hg H5a1 among both RoIA and ME ancient samples and present-day Poles, and the identification of N1a1a2 haplotype in RoIA and contemporary Poles is consistent with the idea of continuity of maternal lineages from at least Roman Iron Age in the region. Our data demonstrates that present-day Western Slavs, among analyzed Europeans, exhibit a mtDNA profile that is more similar to one found among ancient inhabitants of Central Europe. This observation appears to be in concordance with the autochthonous hypothesis. Studies on the genetic profiles of other ancient Slavic populations, especially employing nuclear markers, are necessary for further resolution of the complex origin of the Slavs.

## Supporting Information

Figure S1 **Alignments of cloned aDNA sequences analyzed in this study.** The first lines report the revised Cambridge Reference Sequence (rCRS) with the numbering of the nucleotide positions.
(PDF)

Table S1 **Detailed information about all samples used in the study involving their repository names, geographical location, context and references.**
(XLS)

Table S2 **PCR primers used in the present study.**
(DOCX)

Table S3 **Modern and ancient populations used in the comparative analysis.**
(DOCX)

Table S4 **Haplotype sharing analysis.**
(XLSX)

Table S5 ***F_ST_*** **distances by Slatkin's based on haplogroup frequencies (P<0.01).**
(DOCX)
