# Supplementary material for: Ancient DNA Reveals Matrilineal Continuity in Present-Day Poland over the Last Two Millennia
Source: PLoS One. 2014 Oct 22;9(10):e110839. doi: 10.1371/journal.pone.0110839 (PMC4206425; doi:10.1371/journal.pone.0110839)
Supplement: Figure S1 — Alignments of cloned aDNA sequences analyzed in this study. The first lines report the revised Cambridge Reference Sequence (rCRS) with the numbering of the nucleotide positions. (PDF) [file pone.0110839.s001.pdf]

```
Sample R1 (RoIA)

1111111111111111111111111111111111111111111111111111111111111111111111111111111111111111111111111111111111111111111111111111111111111111111111111111111111111111111111111111111111111111111111111111111111111111111111111111111111111111111111111111111111111111111111111111111111111111111111111111111111111111111111111111111111111111111111
6666666666666666666666666666666666666666666666666666666666666666666666666666666666666666666666666666666666666666666666666666666666666666666666666666666666666666666666666666666666666666666666666666666666666666666666666666666666666666666666666666666666666666666666666666666666666666666666666666666666666666666666666666666666666666666666
0000000000000000000000000000000000000000000000000111111111111111111111111111111111111111111111111111111111111111111111111111111111111111111111111111111112222222222222222222222222222222222222222222222222222222222222222222222222222222222222222222222222222222223333333333333333333333333333333333333333333333333333333333333333333333333333333333333
5555555556666666666777777777788888888889999999999000000000011111111112222222222333333333344444444445555555555666666666677777777778888888888999999999900000000001111111111222222222233333333334444444444555555555566666666667777777777888888888899999999990000000000111111111122222222223333333333444444444455555555556666666666777777777788888
1234567890123456789012345678901234567890123456789012345678901234567890123456789012345678901234567890123456789012345678901234567890123456789012345678901234567890123456789012345678901234567890123456789012345678901234567890123456789012345678901234567890123456789012345678901234567890123456789012345678901234567890123456789012345678901234

CRS             ACCACCCAAGTATTGACTCACCCATCAACAACCGCTATGTATTTCGTACATTACTGCCAGCCACCATGAATATTGTACGGTACCATAAATACTTGACCACCTGTAGTACATAAAAACCCAATCCACATCAAAACCCCCTCCCCATGCTTACAAGCAAGTACAGCAATCAACCCTCAACTATCACACATCAACTGCAACTCCAAAGCCACCCCTCACCCACTAGGATACCAACAAACCTACCCACCCTTAACAGTACATAGTACATAAAGCCATTTACCGTACATAGCACATTACAGTCAAATCCCTTCTCGTCCCCATGGATGACCCCCCTCAG

clone 1 tooth 1    L16050...............................................................................H16130
clone 2 tooth 1    ...............................................................................
clone 3 tooth 1    ...............................................................................
clone 4 tooth 1    ...............................................................................
clone 5 tooth 1    ...............................................................................
clone 6 tooth 1    ...............................................................................
clone 7 tooth 1    ...............................................................................
clone 8 tooth 1    ...............................................................................
clone 1 tooth 1    L16119............................................................................H16196
clone 2 tooth 1    ............................................................................
clone 3 tooth 1    ............................................................................
clone 4 tooth 1    ............................................................................
clone 5 tooth 1    ............................................................................
clone 6 tooth 1    ......................T.....................................................
clone 7 tooth 1    .............TT.......T...........................................TT........
clone 8 tooth 1    .......................................................T....................
clone 9 tooth 1    ...............................A............................................
clone 10 tooth 1   ......................T.............................T..................TT...
clone 11 tooth 1   ............................................................................
clone 12 tooth 2   ..........A..............A..........A.......................................
clone 13 tooth 2   ............................................................................
clone 14 tooth 2   ........T...........................................................T.......
clone 15 tooth 2   ............................................................................
clone 16 tooth 2   .................................................................T..........
clone 17 tooth 2   ............................................................................
clone 18 tooth 2   ............................................................................
clone 19 tooth 2   ............................................................................
clone 1 tooth 1    l16181...................................................................H16249
clone 2 tooth 1    ...................................................................
clone 3 tooth 1    ...................................................................
clone 4 tooth 1    ...................................................................
clone 5 tooth 1    ...................................T...............................
clone 6 tooth 1    .................................................T.T....T..T..T....
clone 7 tooth 1    ......................A........................G...................
clone 8 tooth 1    ......................A............................................
clone 9 tooth 2    ......T........T...............T...T..T.........C..................
clone 10 tooth 2   ...................................................................
clone 11 tooth 2   ...................................................................
clone 12 tooth 2   ...................................................................
clone 13 tooth 2   ...................................................................
clone 1 tooth 1    L16209 ..................................................................................................................................................H16356
clone 2 tooth 1    ..................................................................................................................................................
clone 3 tooth 1    ..............................................T...................................................................................................
clone 4 tooth 1    ........................................................................T.........................................................................
clone 5 tooth 1    .............................................................................................................Y....................................
clone 6 tooth 1    ..................................................................................................................................................
clone 7 tooth 1    ......................................T...........................................................................................................
clone 8 tooth 1    ......................................T...........................................................................................................
clone 9 tooth 2    ..................................A...............................................................................................................
clone 10 tooth 2   ......................................................T.T.T.............T...T.........T..............................T............................
clone 11 tooth 2   ......................................................T.T.T.............T...T.........T..............................T............................
clone 12 tooth 2   ..................................................................................................................................................
clone 13 tooth 2   ..................................................................................................................................................
clone 14 tooth 2   ..................................................................................................................................................
clone 15 tooth 2   ..................................................................................................................................................
clone 16 tooth 2   ...........TT.....T.......T..T.....T..T.TT......................................................T......T..............................T...........

Sample R2 (RoIA)

1111111111111111111111111111111111111111111111111111111111111111111111111111111111111111111111111111111111111111111111111111111111111111111111111111111111111111111111111111111111111111111111111111111111111111111111111111111111111111111111111111111111111111111111111111111111111111111111111111111111111111111111111111111111111111111111
6666666666666666666666666666666666666666666666666666666666666666666666666666666666666666666666666666666666666666666666666666666666666666666666666666666666666666666666666666666666666666666666666666666666666666666666666666666666666666666666666666666666666666666666666666666666666666666666666666666666666666666666666666666666666666666666
0000000000000000000000000000000000000000000000000111111111111111111111111111111111111111111111111111111111111111111111111111111111111111111111111111111112222222222222222222222222222222222222222222222222222222222222222222222222222222222222222222222222222222223333333333333333333333333333333333333333333333333333333333333333333333333333333333333
5555555556666666666777777777788888888889999999999000000000011111111112222222222333333333344444444445555555555666666666677777777778888888888999999999900000000001111111111222222222233333333334444444444555555555566666666667777777777888888888899999999990000000000111111111122222222223333333333444444444455555555556666666666777777777788888
1234567890123456789012345678901234567890123456789012345678901234567890123456789012345678901234567890123456789012345678901234567890123456789012345678901234567890123456789012345678901234567890123456789012345678901234567890123456789012345678901234567890123456789012345678901234567890123456789012345678901234567890123456789012345678901234

CRS             ACCACCCAAGTATTGACTCACCCATCAACAACCGCTATGTATTTCGTACATTACTGCCAGCCACCATGAATATTGTACGGTACCATAAATACTTGACCACCTGTAGTACATAAAAACCCAATCCACATCAAAACCCCCTCCCCATGCTTACAAGCAAGTACAGCAATCAACCCTCAACTATCACACATCAACTGCAACTCCAAAGCCACCCCTCACCCACTAGGATACCAACAAACCTACCCACCCTTAACAGTACATAGTACATAAAGCCATTTACCGTACATAGCACATTACAGTCAAATCCCTTCTCGTCCCCATGGATGACCCCCCTCAG

clone 1 tooth 2    L16050..........................................C...................................AH16130
clone 2 tooth 2    ..........................................C...................................A
clone 3 tooth 2    ..........................................C...................................A
clone 4 tooth 2    ..........................................C...................................A
clone 5 tooth 2    ..........................................C...................................A
clone 6 tooth 2    ..........................................C...................................A
clone 7 tooth 2    ..........................................C...................G...............A
clone 8 tooth 2    ..........................................C.....T.............................A
clone 1 tooth 1    L16119.........A..................................................................H16196
clone 2 tooth 1    .........A..................................................................
clone 3 tooth 1    .........A....T.............................................................
clone 4 tooth 1    .........AA...............G.................................................
clone 5 tooth 1    .........A..................................................................
clone 6 tooth 1    .........A..................................................................
clone 7 tooth 1    .........A..........................A.......................................
clone 8 tooth 1    .........A..................................................................
clone 9 tooth 2    .........A..................................................................
clone 10 tooth 2   .........A..................................................................
clone 11 tooth 2   .........A..................................................................
clone 12 tooth 2   .........A..................................................................
clone 13 tooth 2   .........A..................................................................
clone 14 tooth 2   .........A..................................................................
clone 1 tooth 1    L16181...................................................................H16249
clone 2 tooth 1    ...................................................................
clone 3 tooth 1    ...................................................................
clone 4 tooth 1    ..........................................T..T...T...T.............
clone 5 tooth 1    ...................................................................
clone 6 tooth 1    ...................................................................
clone 7 tooth 1    ...................................................................
clone 8 tooth 2    ...................................T....T..........................
clone 9 tooth 2    ........................................................A..........
clone 10 tooth 2   ..............................A....................................
clone 11 tooth 2   ........................................................A..........
clone 1 tooth 1    L16209.........................................................................................................G........................................H16356
clone 2 tooth 1    ..........................T........T.....................................................................G...............T........................
clone 3 tooth 1    ...........T...................................T.T..T..............T.......T.......T.....................G.....................................T..
clone 4 tooth 1    ..................T..........T............................................................T..............G........................................
clone 1 tooth 1    .........................................T....TT..TTT.T.T................................................G..............G.........................
clone 2 tooth 1    .T.........T.T..........................T.....T.....T.T.TT...............................................G........................................
clone 3 tooth 2    .......C...........................................T..............................................................................................
clone 4 tooth 2    .........................................................................................................G........................................
clone 5 tooth 2    .............................................A...........................................................G........................................
clone 6 tooth 2    ..................T.....T................................................................................G........................................
clone 7 tooth 2    .........................................................................................................G........................................
clone 8 tooth 2    .......C...........................................T..............................................................................................
clone 9 tooth 2    .........................................................................................................G........................................

Sample R3 (RoIA)

1111111111111111111111111111111111111111111111111111111111111111111111111111111111111111111111111111111111111111111111111111111111111111111111111111111111111111111111111111111111111111111111111111111111111111111111111111111111111111111111111111111111111111111111111111111111111111111111111111111111111111111111111111111111111111111111
6666666666666666666666666666666666666666666666666666666666666666666666666666666666666666666666666666666666666666666666666666666666666666666666666666666666666666666666666666666666666666666666666666666666666666666666666666666666666666666666666666666666666666666666666666666666666666666666666666666666666666666666666666666666666666666666
0000000000000000000000000000000000000000000000000111111111111111111111111111111111111111111111111111111111111111111111111111111111111111111111111111111112222222222222222222222222222222222222222222222222222222222222222222222222222222222222222222222222222222223333333333333333333333333333333333333333333333333333333333333333333333333333333333333
5555555556666666666777777777788888888889999999999000000000011111111112222222222333333333344444444445555555555666666666677777777778888888888999999999900000000001111111111222222222233333333334444444444555555555566666666667777777777888888888899999999990000000000111111111122222222223333333333444444444455555555556666666666777777777788888
1234567890123456789012345678901234567890123456789012345678901234567890123456789012345678901234567890123456789012345678901234567890123456789012345678901234567890123456789012345678901234567890123456789012345678901234567890123456789012345678901234567890123456789012345678901234567890123456789012345678901234567890123456789012345678901234

CRS             ACCACCCAAGTATTGACTCACCCATCAACAACCGCTATGTATTTCGTACATTACTGCCAGCCACCATGAATATTGTACGGTACCATAAATACTTGACCACCTGTAGTACATAAAAACCCAATCCACATCAAAACCCCCTCCCCATGCTTACAAGCAAGTACAGCAATCAACCCTCAACTATCACACATCAACTGCAACTCCAAAGCCACCCCTCACCCACTAGGATACCAACAAACCTACCCACCCTTAACAGTACATAGTACATAAAGCCATTTACCGTACATAGCACATTACAGTCAAATCCCTTCTCGTCCCCATGGATGACCCCCCTCAG

clone 1 tooth 1    L16050...............................................................................H16130
clone 2 tooth 1    ......................................A.....A.......................A..........
clone 3 tooth 1    ...............................................................................
clone 4 tooth 1    ...............................................................................
clone 5 tooth 1    ...............................................................................
clone 6 tooth 1    ...............................................................................
clone 7 tooth 2    .....T............................T............................................
clone 8 tooth 2    ..T.......................................................................A....
clone 9 tooth 2    ..............................T.....C..........................................
clone 10 tooth 2   ..T.................T..........................................................
clone 11 tooth 2   ............................................A..................................
clone 1 tooth 1    L16119.................................A..........................................H16196
clone 2 tooth 1    .................................A..........................................
clone 3 tooth 1    .................................A..........................................
clone 4 tooth 1    .................................A..........................................
clone 5 tooth 1    .................................A..........................................
clone 6 tooth 1    .................................A..........................................
clone 7 tooth 1    .................................A..........................................
clone 8 tooth 2    .................................A..........................................
clone 9 tooth 2    .................................A..........................................
```

```
clone 10 tooth 2  .................................A...........................................
clone 11 tooth 2  .................................A...........................................
clone 12 tooth 2  .................................A...........................................
clone 13 tooth 2  ..............................Y..A...................................Y..YY...
clone 14 tooth 2  .................................A...........................................
clone 15 tooth 2  .................................A...........................................
clone 16 tooth 2  .................................A...........................................
clone 17 tooth 2  .................................A........................C....T.............
clone 1 tooth 1   L16181....................................................................H16249
clone 2 tooth 1   ..............A.....................................................
clone 3 tooth 1   ....................................................................
clone 4 tooth 1   ....................................................................
clone 5 tooth 1   ....................................................................
clone 6 tooth 1   .......................A............................................
clone 7 tooth 1   ....................................................................
clone 8 tooth 2   ....................T..........................................T..T.
clone 9 tooth 2   ....................................................................
clone 10 tooth 2  ....................................................................
clone 11 tooth 2  ....................................................................
clone 12 tooth 2  ....................................................................
clone 13 tooth 2  ....................................................................
clone 1 tooth 1   L16209..............................................................................................C....................................................H16356
clone 2 tooth 1   ..............................................................................................C....................................................
clone 3 tooth 1   ..............................................................................................C....................................................
clone 4 tooth 1   ...............................................................A..............................C....................................................
clone 5 tooth 1   .............T......................................T.T..T....................................C....................................................
clone 6 tooth 1   ..............................................................................................C..............A.........A...........................
clone 7 tooth 2   ...........T......T...........................................................................C...................................T................
clone 8 tooth 2   .................................................T............................................C....................................................
clone 9 tooth 2   .T..T.............T.....................................................T.....................C......................TT............................
clone 10 tooth 2  .............................................Y................................................C....................................................
clone 11 tooth 2  ............................................................................T.................C............................T.......................
clone 12 tooth 2  ...........T.T.T..............................................................................C....................................................
clone 13 tooth 2  ........................................T.....................................................C........................T.........................T.
clone 14 tooth 2  ....................................................................T.........................C....................................................
clone 15 tooth 2  ........................T.T..T........................T.T........................................T.................................................
clone 16 tooth 2  ..............................................................................................C....................................................
clone 17 tooth 2  ..............................................................................................C....................................................
clone 18 tooth 2  ..............................................................................................C....................................................
clone 19 tooth 2  ..............................................................................................C....................................................
```

Sample R4 (RoIA)

```
      1111111111111111111111111111111111111111111111111111111111111111111111111111111111111111111111111111111111111111111111111111111111111111111111111111111111111111111111111111111111111111111111111111111111111111111111111111111111111111111111111111111111111111111111111111111111111111111111111111111111111111111111111111111111111111111111
      6666666666666666666666666666666666666666666666666666666666666666666666666666666666666666666666666666666666666666666666666666666666666666666666666666666666666666666666666666666666666666666666666666666666666666666666666666666666666666666666666666666666666666666666666666666666666666666666666666666666666666666666666666666666666666666666
      0000000000000000000000000000000000000000000000000111111111111111111111111111111111111111111111111111111111111111111111111111111111111111111111111111111122222222222222222222222222222222222222222222222222222222222222222222222222222222222222222222222222222222222222222222222222222222223333333333333333333333333333333333333333333333333333333333333333333333333333333333333
      5555555556666666666777777777788888888889999999999000000000011111111112222222222333333333344444444445555555555666666666677777777778888888888999999999900000000001111111111222222222233333333334444444444555555555566666666667777777777888888888899999999990000000000111111111122222222223333333333444444444455555555556666666666777777777788888
      1234567890123456789012345678901234567890123456789012345678901234567890123456789012345678901234567890123456789012345678901234567890123456789012345678901234567890123456789012345678901234567890123456789012345678901234567890123456789012345678901234567890123456789012345678901234567890123456789012345678901234567890123456789012345678901234

CRS   ACCACCCAAGTATTGACTCACCCATCAACAACCGCTATGTATTTCGTACATTACTGCCAGCCACCATGAATATTGTACGGTACCATAAATACTTGACCACCTGTAGTACATAAAAACCCAATCCACATCAAAACCCCCTCCCCATGCTTACAAGCAAGTACAGCAATCAACCCTCAACTATCACACATCAACTGCAACTCCAAAGCCACCCCTCACCCACTAGGATACCAACAAACCTACCCACCCTTAACAGTACATAGTACATAAAGCCATTTACCGTACATAGCACATTACAGTCAAATCCCTTCTCGTCCCCATGGATGACCCCCCTCAG

clone 1 tooth 1   L16050............................T...................................................H16130
clone 2 tooth 1   ................................................................................
clone 3 tooth 1   .............................................T........T.........................
clone 4 tooth 1   ................................................................................
clone 5 tooth 2   ......................T.....T.........................T.........................
clone 6 tooth 2   .........A............................A.............................A..........A
clone 7 tooth 2   ....T...........................T.T..........T..................................
clone 1 tooth 1   L16119.............................................................................H16196
clone 2 tooth 1   .............................................................................
clone 3 tooth 1   .............................................................................
clone 4 tooth 1   ...............................................T.............................
clone 5 tooth 1   .............................................................................
clone 6 tooth 1   ............................................................T................
clone 7 tooth 1   .................................................T...................T..T.T..
clone 8 tooth 2   .............T.................T.............................................
clone 9 tooth 2   .......................................T.....................................
clone 10 tooth 2  .......................................T.....................................
clone 11 tooth 2  .........A...................................................................
clone 12 tooth 2  ............................T..........T.....................................
clone 13 tooth 2  .............T.............TT.T........................................T.....
clone 14 tooth 2  .........A...................................................................
clone 15 tooth 2  ...........C...........................T....................T...T..T.........
clone 16 tooth 2  ........T.....T.....................................................T.....T..
clone 17 tooth 2  .............T...............................................................
clone 1 tooth 2   L16181....................................................................H16249
clone 2 tooth 2   ..........TT...T...T...................T............................
clone 3 tooth 2   ....................................................................
clone 4 tooth 2   ...................T.........T.........TT...........................
clone 5 tooth 2   ...................T.....................T..........................
clone 1 tooth 1   L16209...................................................................................................................................................H16356
clone 2 tooth 1   ...................................................................................................................................................
clone 3 tooth 1   ......................................................T.T...................................................................T......................
clone 4 tooth 1   ...................................................................................................................................................
clone 5 tooth 1   ...................................................................................................................................................
clone 6 tooth 1   ...A...............................................................................................................................................
clone 7 tooth 2   ...................................................................................................................................................
clone 8 tooth 2   ...................................................................................................................................................
clone 9 tooth 2   ...................................................................................................................................................
clone 10 tooth 2  ......................T...............T...............T...............TT.......T...................................................................
clone 11 tooth 2  ......................T...............T...............T...............TT.......T...................................................................
clone 12 tooth 2  ...................................................................................................................................................
```

Sample R5 (RoIA)

```
      1111111111111111111111111111111111111111111111111111111111111111111111111111111111111111111111111111111111111111111111111111111111111111111111111111111111111111111111111111111111111111111111111111111111111111111111111111111111111111111111111111111111111111111111111111111111111111111111111111111111111111111111111111111111111111111111
      6666666666666666666666666666666666666666666666666666666666666666666666666666666666666666666666666666666666666666666666666666666666666666666666666666666666666666666666666666666666666666666666666666666666666666666666666666666666666666666666666666666666666666666666666666666666666666666666666666666666666666666666666666666666666666666666
      0000000000000000000000000000000000000000000000000111111111111111111111111111111111111111111111111111111111111111111111111111111111111111111111111111111122222222222222222222222222222222222222222222222222222222222222222222222222222222222222222222222222222222222222222222222222222222223333333333333333333333333333333333333333333333333333333333333333333333333333333333333
      5555555556666666666777777777788888888889999999999000000000011111111112222222222333333333344444444445555555555666666666677777777778888888888999999999900000000001111111111222222222233333333334444444444555555555566666666667777777777888888888899999999990000000000111111111122222222223333333333444444444455555555556666666666777777777788888
      1234567890123456789012345678901234567890123456789012345678901234567890123456789012345678901234567890123456789012345678901234567890123456789012345678901234567890123456789012345678901234567890123456789012345678901234567890123456789012345678901234567890123456789012345678901234567890123456789012345678901234567890123456789012345678901234

CRS   ACCACCCAAGTATTGACTCACCCATCAACAACCGCTATGTATTTCGTACATTACTGCCAGCCACCATGAATATTGTACGGTACCATAAATACTTGACCACCTGTAGTACATAAAAACCCAATCCACATCAAAACCCCCTCCCCATGCTTACAAGCAAGTACAGCAATCAACCCTCAACTATCACACATCAACTGCAACTCCAAAGCCACCCCTCACCCACTAGGATACCAACAAACCTACCCACCCTTAACAGTACATAGTACATAAAGCCATTTACCGTACATAGCACATTACAGTCAAATCCCTTCTCGTCCCCATGGATGACCCCCCTCAG

clone 1 tooth 1   L16050................................................................................H16130
clone 2 tooth 1   ................................................................................
clone 3 tooth 1   ................................................................................
clone 4 tooth 1   ....................T....................................T......T...............
clone 5 tooth 1   ................................................................................
clone 6 tooth 1   ................................................................................
clone 7 tooth 2   .........................................................................C......
clone 8 tooth 2   .................................A....A.........................................
clone 9 tooth 2   ......T.........................................................................
clone 10 tooth 2  ................................................................................
clone 1 tooth 1   L16119................................................................C.....C......H16196
clone 2 tooth 1   ................................................................C...T....T...
clone 3 tooth 1   ................................................................C.....C......
clone 4 tooth 1   ...............................T................................C............
clone 5 tooth 1   ................................................................C.....C......
clone 6 tooth 2   ....................................A...........................C.....C......
clone 7 tooth 2   ................................................................C.....-......
clone 8 tooth 2   .........................A.......A..............................C.....-......
clone 9 tooth 2   ................................................................C.....C......
clone 10 tooth 2  .............................Y..................................C.....C......
clone 11 tooth 2  ................................................................C.....C......
clone 12 tooth 2  ................................................................T.T..TCT..T..
clone 13 tooth 2  ................................................................C.....C......
clone 1 tooth 1   L16181.......C.....................................................C......H16249
clone 2 tooth 1   .C.....C............................................................
clone 3 tooth 1   .......-............................................................
clone 4 tooth 2   .C.....C......................................................A.....
clone 5 tooth 2   .C.....C.......T....................................................
clone 6 tooth 2   .C.....C............................................................
clone 7 tooth 2   .......C..................A.........................................
clone 1 tooth 1   H16209.......................................................................................................G...........................................H16356
clone 2 tooth 1   .............................T................T.....T....T.........................................................................................
clone 3 tooth 2   ...................................................................................................................................................
clone 4 tooth 2   .......C.................G....................................................................................................................G....
clone 5 tooth 2   ......................T.................................T............T..T........T...............T..T...........................................T.T
clone 6 tooth 2   ...................................................................................................................................................
clone 7 tooth 2   .......C.................G.......C.................................................................................................................
clone 8 tooth 2   ...................................................................................................................................................
```

```
Sample R6 (RoIA)
                    1111111111111111111111111111111111111111111111111111111111111111111111111111111111111111111111111111111111111111111111111111111111111111111111111111111111111111111111111111111111111111111111111111111111111111111111111111111111111111111111111111111111111111111111111111111111111111111111111111111111111111111111111111111111111111
                    6666666666666666666666666666666666666666666666666666666666666666666666666666666666666666666666666666666666666666666666666666666666666666666666666666666666666666666666666666666666666666666666666666666666666666666666666666666666666666666666666666666666666666666666666666666666666666666666666666666666666666666666666666666666666666
                    0000000000000000000000000000000000000000000000000111111111111111111111111111111111111111111111111111111111111111111111111111111111111111111111111111122222222222222222222222222222222222222222222222222222222222222222222222222222222222222222222222222222222222223333333333333333333333333333333333333333333333333333333333333333333333333333333333333
                    5555555556666666666777777777788888888889999999999000000000011111111112222222222333333333344444444445555555555666666666677777777778888888888999999999900000000001111111111222222222233333333334444444444555555555566666666667777777777888888888899999999990000000000111111111122222222223333333333444444444455555555556666666666777777777788888
                    1234567890123456789012345678901234567890123456789012345678901234567890123456789012345678901234567890123456789012345678901234567890123456789012345678901234567890123456789012345678901234567890123456789012345678901234567890123456789012345678901234567890123456789012345678901234567890123456789012345678901234567890123456789012345678901234

CRS                 ACCACCCAAGTATTGACTCACCCATCAACAACCGCTATGTATTTCGTACATTACTGCCAGCCACCATGAATATTGTACGGTACCATAAATACTTGACCACCTGTAGTACATAAAAACCCAATCCACATCAAAACCCCCTCCCCATGCTTACAAGCAAGTACAGCAATCAACCCTCAACTATCACACATCAACTGCAACTCCAAAGCCACCCCTCACCCACTAGGATACCAACAAACCTACCCACCCTTAACAGTACATAGTACATAAAGCCATTTACCGTACATAGCACATTACAGTCAAATCCCTTCTCGTCCCCATGGATGACCCCCCTCAG

clone 1 tooth 1                                                                    L16119............................................................................H16196
clone 2 tooth 1                                                                           ............................Y.Y.................................YYY.........
clone 3 tooth 1                                                                           ............................................................................
clone 4 tooth 1                                                                           ................................................................T.T.T..T....
clone 5 tooth 1                                                                           ...............................................TTT...T..............T.T.....
clone 6 tooth 2                                                                           ............................................................................
clone 7 tooth 2                                                                           ....................................Y.......................................
clone 8 tooth 2                                                                           ............................................................................
clone 9 tooth 2                                                                           .................................A......................................T...
clone 10 tooth 2                                                                          ............................................................................
clone 11 tooth 2                                                                          ............................................................................
clone 1 tooth 2                                                                           ....................Y.......................................................
clone 2 tooth 2                                                                                                                                                  L16181........T..........T...T............................T..............H16249
clone 3 tooth 2                                                                                                                                                        ...................................................................
clone 4 tooth 2                                                                                                                                                        ..........................................C........................
clone 5 tooth 2                                                                                                                                                        ...............................Y...................................
clone 1 tooth 1                                                                                                                                                        ...................................................................
clone 2 tooth 1                                                                                                                                                                              L16209....T.............................................T....T.T..................................G...............T.....................................H16356
clone 3 tooth 1                                                                                                                                                                                    .T.....................................................................................................................................A..........
clone 4 tooth 1                                                                                                                                                                                    ....T.............................................T....T.T..................................................T.....................................
clone 5 tooth 1                                                                                                                                                                                    ....Y..................................................................................................................................A..........
clone 6 tooth 2                                                                                                                                                                                    ...........T............T...............................................T....T......T......T..................................T...................
clone 7 tooth 2                                                                                                                                                                                    ........................................T.........T.TT.T.T.T.T................................................T...................................
clone 8 tooth 2                                                                                                                                                                                    ......................................................................................................................A................A..........
clone 9 tooth 2                                                                                                                                                                                    .............................................A....................................................................................................
clone 10 tooth 2                                                                                                                                                                                   .............................Y..Y...............................................YY....Y...........................................................
clone 11 tooth 2                                                                                                                                                                                   ..........................................................................................................G.......................................

Sample R7 (RoIA)
                    1111111111111111111111111111111111111111111111111111111111111111111111111111111111111111111111111111111111111111111111111111111111111111111111111111111111111111111111111111111111111111111111111111111111111111111111111111111111111111111111111111111111111111111111111111111111111111111111111111111111111111111111111111111111111111
                    6666666666666666666666666666666666666666666666666666666666666666666666666666666666666666666666666666666666666666666666666666666666666666666666666666666666666666666666666666666666666666666666666666666666666666666666666666666666666666666666666666666666666666666666666666666666666666666666666666666666666666666666666666666666666666
                    0000000000000000000000000000000000000000000000000111111111111111111111111111111111111111111111111111111111111111111111111111111111111111111111111111122222222222222222222222222222222222222222222222222222222222222222222222222222222222222222222222222222222222223333333333333333333333333333333333333333333333333333333333333333333333333333333333333
                    5555555556666666666777777777788888888889999999999000000000011111111112222222222333333333344444444445555555555666666666677777777778888888888999999999900000000001111111111222222222233333333334444444444555555555566666666667777777777888888888899999999990000000000111111111122222222223333333333444444444455555555556666666666777777777788888
                    1234567890123456789012345678901234567890123456789012345678901234567890123456789012345678901234567890123456789012345678901234567890123456789012345678901234567890123456789012345678901234567890123456789012345678901234567890123456789012345678901234567890123456789012345678901234567890123456789012345678901234567890123456789012345678901234

CRS                 ACCACCCAAGTATTGACTCACCCATCAACAACCGCTATGTATTTCGTACATTACTGCCAGCCACCATGAATATTGTACGGTACCATAAATACTTGACCACCTGTAGTACATAAAAACCCAATCCACATCAAAACCCCCTCCCCATGCTTACAAGCAAGTACAGCAATCAACCCTCAACTATCACACATCAACTGCAACTCCAAAGCCACCCCTCACCCACTAGGATACCAACAAACCTACCCACCCTTAACAGTACATAGTACATAAAGCCATTTACCGTACATAGCACATTACAGTCAAATCCCTTCTCGTCCCCATGGATGACCCCCCTCAG

clone 1 tooth 1                                                                    L16119...................G..T.....................................................H16196
clone 2 tooth 1                                                                           .................................................T................T.T..T....
clone 3 tooth 1                                                                           ......................T................T....................................
clone 4 tooth 1                                                                           ............................................................................
clone 5 tooth 1                                                                           ............................................................................
clone 6 tooth 2                                                                           ............................................................................
clone 7 tooth 2                                                                           .............T..............................................................
clone 8 tooth 2                                                                           ............................................................................
clone 9 tooth 2                                                                           ............................................................................
clone 1 tooth 1                                                                                                                                                                              L16209...............................................T......T...........................................................................................H16356
clone 2 tooth 1                                                                                                                                                                                    ..................................................................................................................................................
clone 3 tooth 1                                                                                                                                                                                    .........................G........................................................................................................................
clone 4 tooth 1                                                                                                                                                                                    ..............A...................................................................................................................................
clone 5 tooth 1                                                                                                                                                                                    ..................................................................................................................................................
clone 6 tooth 1                                                                                                                                                                                    .T................T..................................................................................................T....T.......................
clone 7 tooth 2                                                                                                                                                                                    ..................................................................................................C....................A..........................
clone 8 tooth 2                                                                                                                                                                                    ................................................................................G.................................................................
clone 9 tooth 2                                                                                                                                                                                    ..................................................................................................................................................
clone 10 tooth 2                                                                                                                                                                                   ..................................................................................................................................................
clone 11 tooth 2                                                                                                                                                                                   ...............T..T...............................................................................................................................
clone 12 tooth 2                                                                                                                                                                                   .......................................T.................T........................................................................................

Sample R8 (RoIA)
                    1111111111111111111111111111111111111111111111111111111111111111111111111111111111111111111111111111111111111111111111111111111111111111111111111111111111111111111111111111111111111111111111111111111111111111111111111111111111111111111111111111111111111111111111111111111111111111111111111111111111111111111111111111111111111111
                    6666666666666666666666666666666666666666666666666666666666666666666666666666666666666666666666666666666666666666666666666666666666666666666666666666666666666666666666666666666666666666666666666666666666666666666666666666666666666666666666666666666666666666666666666666666666666666666666666666666666666666666666666666666666666666
                    0000000000000000000000000000000000000000000000000111111111111111111111111111111111111111111111111111111111111111111111111111111111111111111111111111122222222222222222222222222222222222222222222222222222222222222222222222222222222222222222222222222222222222223333333333333333333333333333333333333333333333333333333333333333333333333333333333333
                    5555555556666666666777777777788888888889999999999000000000011111111112222222222333333333344444444445555555555666666666677777777778888888888999999999900000000001111111111222222222233333333334444444444555555555566666666667777777777888888888899999999990000000000111111111122222222223333333333444444444455555555556666666666777777777788888
                    1234567890123456789012345678901234567890123456789012345678901234567890123456789012345678901234567890123456789012345678901234567890123456789012345678901234567890123456789012345678901234567890123456789012345678901234567890123456789012345678901234567890123456789012345678901234567890123456789012345678901234567890123456789012345678901234

CRS                 ACCACCCAAGTATTGACTCACCCATCAACAACCGCTATGTATTTCGTACATTACTGCCAGCCACCATGAATATTGTACGGTACCATAAATACTTGACCACCTGTAGTACATAAAAACCCAATCCACATCAAAACCCCCTCCCCATGCTTACAAGCAAGTACAGCAATCAACCCTCAACTATCACACATCAACTGCAACTCCAAAGCCACCCCTCACCCACTAGGATACCAACAAACCTACCCACCCTTAACAGTACATAGTACATAAAGCCATTTACCGTACATAGCACATTACAGTCAAATCCCTTCTCGTCCCCATGGATGACCCCCCTCAG

clone 1 tooth 1                                                                    L16119............................................................................H16196
clone 2 tooth 1                                                                           ...........................................................T....T.....T.....
clone 3 tooth 1                                                                           ............................................................................
clone 4 tooth 1                                                                           ...........A.............A.......A..........................................
clone 5 tooth 1                                                                           .....A.............................G........................................
clone 6 tooth 1                                                                           ...........A................................................................
clone 7 tooth 2                                                                           .........................A.......A..........................................
clone 8 tooth 2                                                                           ............................................................................
clone 9 tooth 2                                                                           ............................................................................
clone 10 tooth 2                                                                          ............................................................................
clone 11 tooth 2                                                                          ......................T.....................................................
clone 1 tooth 1                                                                                                                                                  L16181...................................................................H16249
clone 2 tooth 1                                                                                                                                                        ...................................................................
clone 3 tooth 1                                                                                                                                                        ...................................................................
clone 4 tooth 1                                                                                                                                                        ...................................................................
clone 5 tooth 1                                                                                                                                                        ...................................................................
clone 6 tooth 2                                                                                                                                                        ...........T...T.......T............T..TTT.........................
clone 7 tooth 2                                                                                                                                                        ...................................................................
clone 8 tooth 2                                                                                                                                                        ..............A.......A...A...................................A....
clone 9 tooth 2                                                                                                                                                        ...................................................................
clone 10 tooth 2                                                                                                                                                       ...................................................................
clone 1 tooth 1                                                                                                                                                                              L16209................................................................................................................................Y.................H16356
clone 2 tooth 1                                                                                                                                                                                    ..................................................T..................................................................T............................
clone 3 tooth 1                                                                                                                                                                                    ................................................................Y..................................A..............................................
clone 4 tooth 1                                                                                                                                                                                    ................................................................................................................................T.................
clone 5 tooth 1                                                                                                                                                                                    ................................................................................................................................T.................
clone 6 tooth 1                                                                                                                                                                                    .................G.............................................A...................................A..............................................
clone 7 tooth 2                                                                                                                                                                                    ..................................................................................................................................................
clone 8 tooth 2                                                                                                                                                                                    ....T...T...TT.T............................................................T.....................................................................
clone 9 tooth 2                                                                                                                                                                                    .......Y............................................Y.Y..YY.Y.....................................................................................
clone 10 tooth 2                                                                                                                                                                                   .T................................................................................................................................................
clone 11 tooth 2                                                                                                                                                                                   ........................................................................................................................................A.........

Sample R9 (RoIA)
                    1111111111111111111111111111111111111111111111111111111111111111111111111111111111111111111111111111111111111111111111111111111111111111111111111111111111111111111111111111111111111111111111111111111111111111111111111111111111111111111111111111111111111111111111111111111111111111111111111111111111111111111111111111111111111111
                    6666666666666666666666666666666666666666666666666666666666666666666666666666666666666666666666666666666666666666666666666666666666666666666666666666666666666666666666666666666666666666666666666666666666666666666666666666666666666666666666666666666666666666666666666666666666666666666666666666666666666666666666666666666666666666
                    0000000000000000000000000000000000000000000000000111111111111111111111111111111111111111111111111111111111111111111111111111111111111111111111111111122222222222222222222222222222222222222222222222222222222222222222222222222222222222222222222222222222222222223333333333333333333333333333333333333333333333333333333333333333333333333333333333333
                    5555555556666666666777777777788888888889999999999000000000011111111112222222222333333333344444444445555555555666666666677777777778888888888999999999900000000001111111111222222222233333333334444444444555555555566666666667777777777888888888899999999990000000000111111111122222222223333333333444444444455555555556666666666777777777788888
                    1234567890123456789012345678901234567890123456789012345678901234567890123456789012345678901234567890123456789012345678901234567890123456789012345678901234567890123456789012345678901234567890123456789012345678901234567890123456789012345678901234567890123456789012345678901234567890123456789012345678901234567890123456789012345678901234

CRS                 ACCACCCAAGTATTGACTCACCCATCAACAACCGCTATGTATTTCGTACATTACTGCCAGCCACCATGAATATTGTACGGTACCATAAATACTTGACCACCTGTAGTACATAAAAACCCAATCCACATCAAAACCCCCTCCCCATGCTTACAAGCAAGTACAGCAATCAACCCTCAACTATCACACATCAACTGCAACTCCAAAGCCACCCCTCACCCACTAGGATACCAACAAACCTACCCACCCTTAACAGTACATAGTACATAAAGCCATTTACCGTACATAGCACATTACAGTCAAATCCCTTCTCGTCCCCATGGATGACCCCCCTCAG

clone 1 tooth 1                                                                    L16119............................T.T.......................T........C.....C.TT...H16196
clone 2 tooth 1                                                                           ...............................................................C.....C......
clone 3 tooth 1                                                                           ...............................................................C.....C......
clone 4 tooth 2                                                                           ...............................................................C.....C......
clone 5 tooth 2                                                                           ...............Y.......................Y.........Y....Y........C.....CY.....
clone 6 tooth 2                                                                           ....................................A..........................C.....-......
clone 7 tooth 2                                                                           ............................................................................
clone 1 tooth 1                                                                                                                                                  L16181.......C..T....T......A............................................H16249
clone 2 tooth 1                                                                                                                                                        .........................................T.........................
clone 3 tooth 1                                                                                                                                                        .C.....C.......................A...................................
clone 4 tooth 2                                                                                                                                                        .C.....C...........................................................
clone 5 tooth 2                                                                                                                                                        .C.....C.........................................................T.
clone 6 tooth 2                                                                                                                                                        ...................................................................
clone 1 tooth 1                                                                                                                                                                              L16209.....................................................C............................................................................................H16356
clone 2 tooth 1                                                                                                                                                                                    ..................................................................................................................................................
clone 3 tooth 1                                                                                                                                                                                    .....................................................C............................................................................................
clone 4 tooth 1                                                                                                                                                                                    .....................................................C............................................................................................
clone 5 tooth 1                                                                                                                                                                                    ....T........................................................................T....................................................................
clone 6 tooth 1                                                                                                                                                                                    .....................................................C............................................................................................
clone 7 tooth 1                                                                                                                                                                                    ....T........................................................................T....................................................................
clone 8 tooth 1                                                                                                                                                                                    .....................................................C............................................................................................
clone 1 tooth 2                                                                                                                                                                                                                      L16249........................Y..........................................H16317
clone 2 tooth 2                                                                                                                                                                                                                            ...................................................................
clone 3 tooth 2                                                                                                                                                                                                                            ...................................................................
clone 4 tooth 2                                                                                                                                                                                                                            ...................................................................
clone 5 tooth 2                                                                                                                                                                                                                            ...................................................................
```

Sample R10 (RoIA)

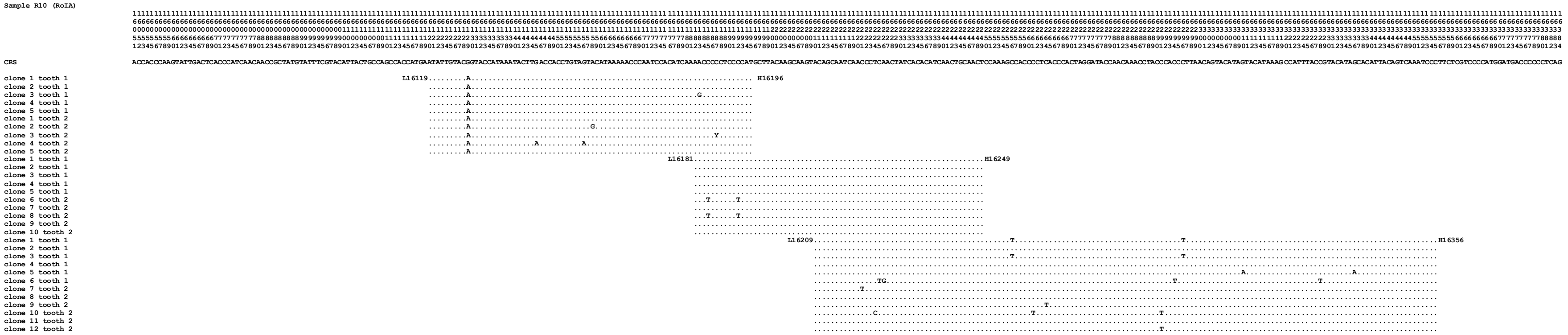

Sample R11 (RoIA)

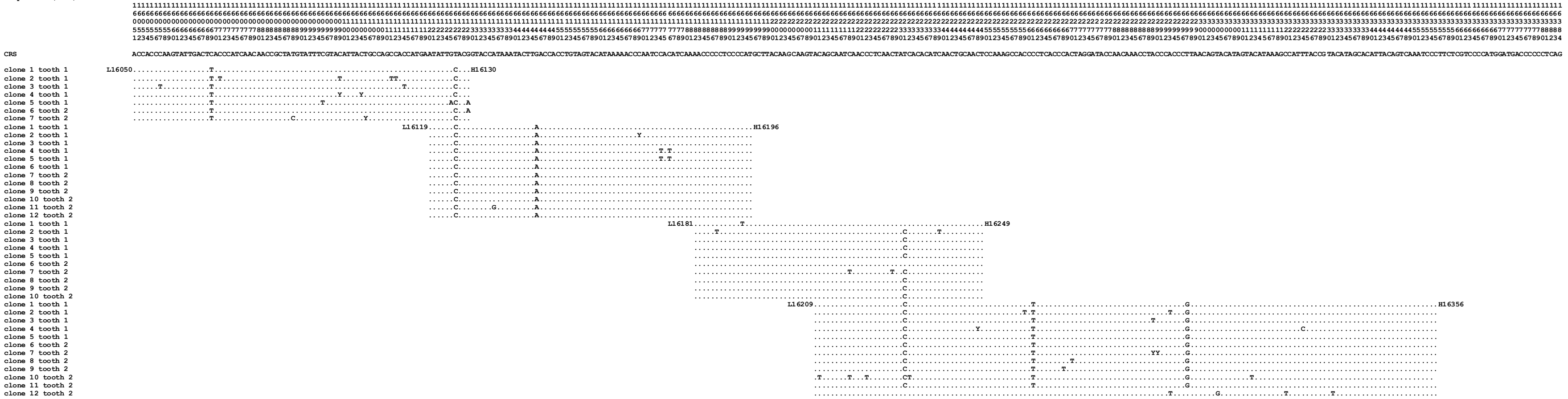

Sample R12 (RoIA)

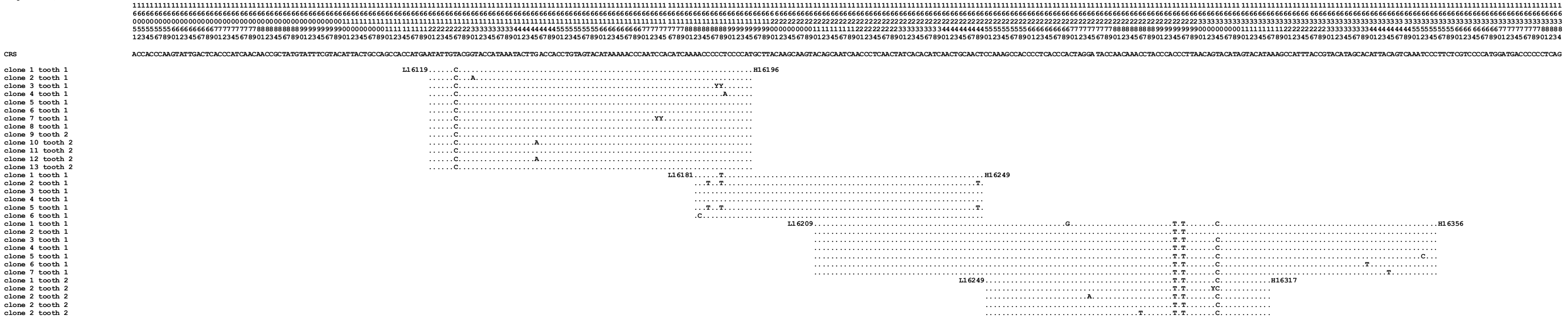

Sample K1 (RoIA)

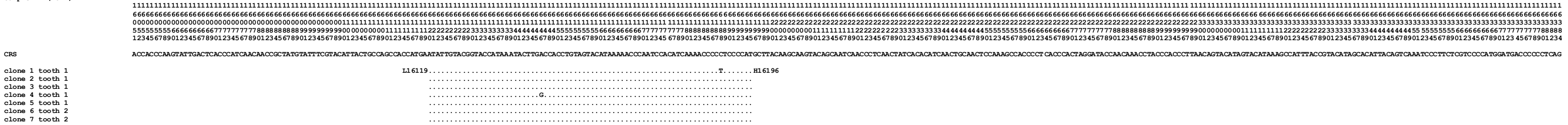

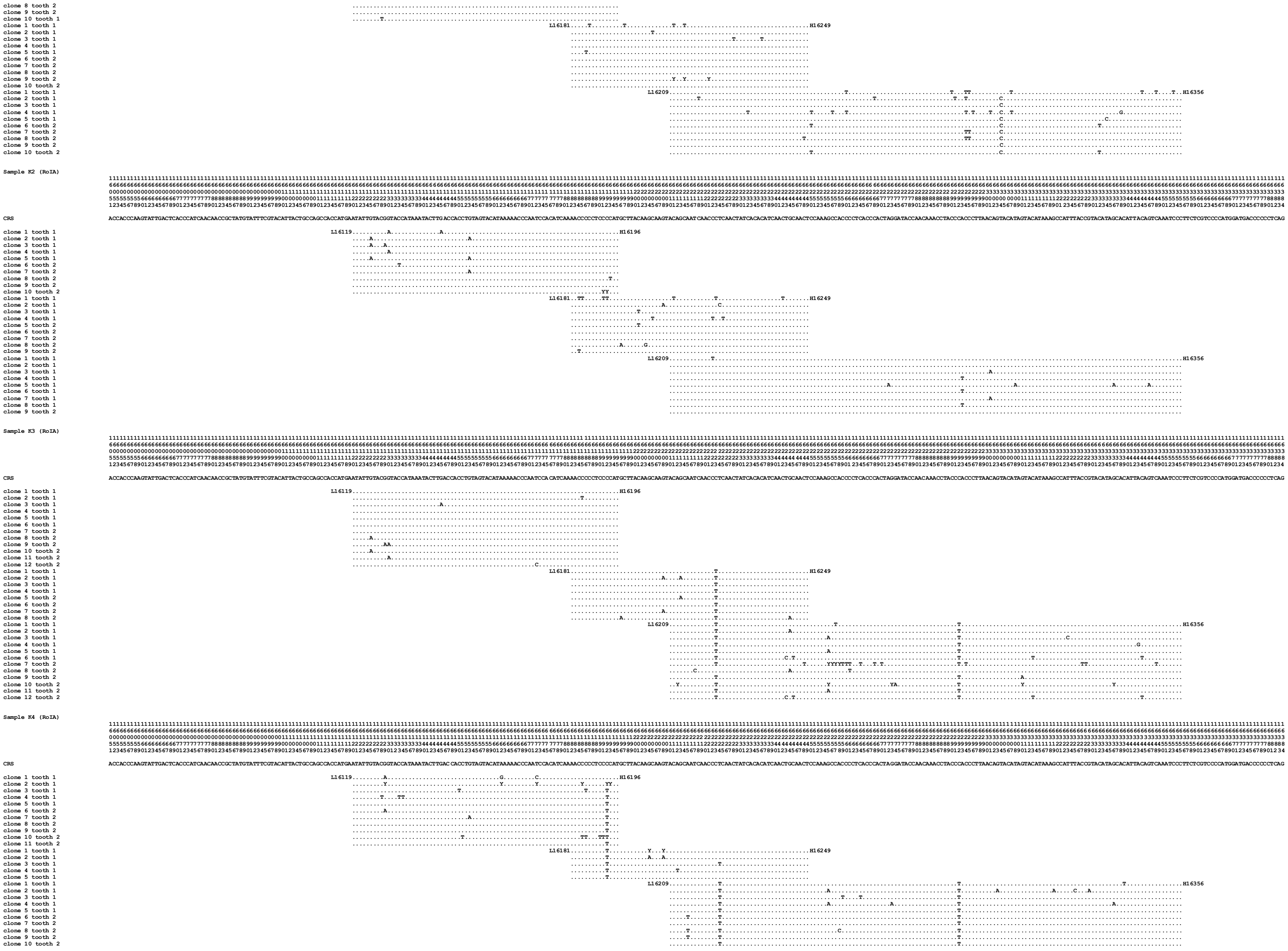
Sample K2 (RoIA)
CRS
Sample K3 (RoIA)
CRS
Sample K4 (RoIA)
CRS

Sample K5 (RoIA)

```
                  1111111111111111111111111111111111111111111111111111111111111111111111111111111111111111111111111111111111111111111111111111111111111111111111111111111111111111111111111111111111111111111111111111111111111111111111111111111111111111111111111111111111111111111111111111111111111111111111111111111111111111111111111111111111111
                  6666666666666666666666666666666666666666666666666666666666666666666666666666666666666666666666666666666666666666666666666666666666666666666666666666666666666666666666666666666666666666666666666666666666666666666666666666666666666666666666666666666666666666666666666666666666666666666666666666666666666666666666666666666666666
                  0000000000000000000000000000000000000000000000000111111111111111111111111111111111111111111111111111111111111111111111111111111111111111111111111111111122222222222222222222222222222222222222222222222222222222222222222222222222222222222222222222222222222222223333333333333333333333333333333333333333333333333333333333333333333333333333333333333
                  5555555556666666666777777777788888888889999999999000000000011111111112222222222333333333344444444445555555555666666666677777777778888888888999999999900000000001111111111222222222233333333334444444444555555555566666666667777777777888888888899999999990000000000111111111122222222223333333333444444444455555555556666666666777777777788888
                  1234567890123456789012345678901234567890123456789012345678901234567890123456789012345678901234567890123456789012345678901234567890123456789012345678901234567890123456789012345678901234567890123456789012345678901234567890123456789012345678901234567890123456789012345678901234567890123456789012345678901234567890123456789012345678901234

CRS               ACCACCCAAGTATTGACTCACCCATCAACAACCGCTATGTATTTCGTACATTACTGCCAGCCACCATGAATATTGTACGGTACCATAAATACTTGACCACCTGTAGTACATAAAAACCCAATCCACATCAAAACCCCCTCCCCATGCTTACAAGCAAGTACAGCAATCAACCCTCAACTATCACACATCAACTGCAACTCCAAAGCCACCCCTCACCCACTAGGATACCAACAAACCTACCCACCCTTAACAGTACATAGTACATAAAGCCATTTACCGTACATAGCACATTACAGTCAAATCCCTTCTCGTCCCCATGGATGACCCCCCTCAG

clone 1 tooth 1                                                                 L16119............................................................................H16196
clone 2 tooth 1                                                                       ......................T................T....................................
clone 3 tooth 1                                                                       ............................................................................
clone 4 tooth 1                                                                       ............................................................................
clone 5 tooth 1                                                                       .........A..................................................................
clone 6 tooth 2                                                                       ............................................................................
clone 7 tooth 2                                                                       .......................................T....................................
clone 8 tooth 2                                                                       ....................................A.......................................
clone 9 tooth 2                                                                       .................................................................T.T..T.....
clone 10 tooth 2                                                                      ....................................A.......................................
clone 1 tooth 1                                                                                                                                                L16181.........................................T.........................H16249
clone 2 tooth 1                                                                                                                                                      ........T..T...T.......T.................T.........................
clone 3 tooth 1                                                                                                                                                      ......................A...A....A.........T.........................
clone 4 tooth 1                                                                                                                                                      .........................................T.........................
clone 5 tooth 1                                                                                                                                                      ........................................TT.........................
clone 6 tooth 2                                                                                                                                                      .........................................T.........................
clone 7 tooth 2                                                                                                                                                      .........................................T.........................
clone 8 tooth 2                                                                                                                                                      ....................................T....T.........................
clone 9 tooth 2                                                                                                                                                      .........................................T.........................
clone 1 tooth 1                                                                                                                                                                            L16209.............T.T.............................................C..............T.....................................................................H16249
clone 2 tooth 1                                                                                                                                                                                  .............T..............................................................T.....................................................................
clone 3 tooth 1                                                                                                                                                                                  .............T........T.................TT.....................T............T........T............................................................
clone 4 tooth 1                                                                                                                                                                                  .............T..................................T...........................T...................................T.................................
clone 5 tooth 1                                                                                                                                                                                  .............T..............................................................T.....................................................................
clone 6 tooth 1                                                                                                                                                                                  .............T..............................................................T....................................A................................
clone 7 tooth 2                                                                                                                                                                                  .............T..............................................................T.....................................................................
clone 8 tooth 2                                                                                                                                                                                  .............T..............................................................T.....................................................................
clone 9 tooth 2                                                                                                                                                                                  .............T..............................................................T.....................................................................
clone 10 tooth 2                                                                                                                                                                                 .............T..............................................................T.....................................................................
clone 11 tooth 2                                                                                                                                                                                 .............T..............................................................T.....................................................................
```

Sample K6 (RoIA)

```
                  1111111111111111111111111111111111111111111111111111111111111111111111111111111111111111111111111111111111111111111111111111111111111111111111111111111111111111111111111111111111111111111111111111111111111111111111111111111111111111111111111111111111111111111111111111111111111111111111111111111111111111111111111111111111111
                  6666666666666666666666666666666666666666666666666666666666666666666666666666666666666666666666666666666666666666666666666666666666666666666666666666666666666666666666666666666666666666666666666666666666666666666666666666666666666666666666666666666666666666666666666666666666666666666666666666666666666666666666666666666666666
                  0000000000000000000000000000000000000000000000000111111111111111111111111111111111111111111111111111111111111111111111111111111111111111111111111111111122222222222222222222222222222222222222222222222222222222222222222222222222222222222222222222222222222222223333333333333333333333333333333333333333333333333333333333333333333333333333333333333
                  5555555556666666666777777777788888888889999999999000000000011111111112222222222333333333344444444445555555555666666666677777777778888888888999999999900000000001111111111222222222233333333334444444444555555555566666666667777777777888888888899999999990000000000111111111122222222223333333333444444444455555555556666666666777777777788888
                  1234567890123456789012345678901234567890123456789012345678901234567890123456789012345678901234567890123456789012345678901234567890123456789012345678901234567890123456789012345678901234567890123456789012345678901234567890123456789012345678901234567890123456789012345678901234567890123456789012345678901234567890123456789012345678901234

CRS               ACCACCCAAGTATTGACTCACCCATCAACAACCGCTATGTATTTCGTACATTACTGCCAGCCACCATGAATATTGTACGGTACCATAAATACTTGACCACCTGTAGTACATAAAAACCCAATCCACATCAAAACCCCCTCCCCATGCTTACAAGCAAGTACAGCAATCAACCCTCAACTATCACACATCAACTGCAACTCCAAAGCCACCCCTCACCCACTAGGATACCAACAAACCTACCCACCCTTAACAGTACATAGTACATAAAGCCATTTACCGTACATAGCACATTACAGTCAAATCCCTTCTCGTCCCCATGGATGACCCCCCTCAG

clone 1 tooth 1                                                                 L16119........................................................................T...H16196
clone 2 tooth 1                                                                       .........A....................................G.........................T...
clone 3 tooth 1                                                                       ........................................................................T...
clone 4 tooth 1                                                                       ..........A.............................................................T...
clone 5 tooth 2                                                                       ........................................................................T...
clone 6 tooth 2                                                                       ........................................................................T...
clone 7 tooth 2                                                                       ........................................................................T...
clone 8 tooth 2                                                                       ........................................................................T...
clone 9 tooth 2                                                                       ........................................................................T...
clone 1 tooth 1                                                                                                                                                L16181..........T........................................................H16249
clone 2 tooth 1                                                                                                                                                      ..........T........................................................
clone 3 tooth 1                                                                                                                                                      ..........T...........A............................................
clone 4 tooth 1                                                                                                                                                      ..........T...........A........A...................................
clone 5 tooth 2                                                                                                                                                      .........................................T.........................
clone 6 tooth 2                                                                                                                                                      ..........T........................................................
clone 7 tooth 2                                                                                                                                                      ..........T........................................................
clone 8 tooth 2                                                                                                                                                      ..........T............................T...........................
clone 9 tooth 2                                                                                                                                                      ..........T........................................................
clone 1 tooth 1                                                                                                                                                                            L16209............................................................T.....................................................................................H16356
clone 2 tooth 1                                                                                                                                                                                  ............................................................T.....................................................................................
clone 3 tooth 1                                                                                                                                                                                  ............................................................T.....................................................................................
clone 4 tooth 1                                                                                                                                                                                  ............................................................T.....................................................................................
clone 5 tooth 1                                                                                                                                                                                  ............................................................T.....................................................................................
clone 6 tooth 1                                                                                                                                                                                  ............................................................T..........................................................A..........................
clone 7 tooth 1                                                                                                                                                                                  ............................................................T.....................................................................................
clone 1 tooth 2                                                                                                                                                                                                                          L16249 ....................T..............................................
clone 2 tooth 2                                                                                                                                                                                                                                 ....................T..A...........................................
clone 3 tooth 2                                                                                                                                                                                                                                 ....................T..............................................
clone 4 tooth 2                                                                                                                                                                                                                                 .....A..............T..AA....................T............G........
```

Sample K7 (RoIA)

```
                  1111111111111111111111111111111111111111111111111111111111111111111111111111111111111111111111111111111111111111111111111111111111111111111111111111111111111111111111111111111111111111111111111111111111111111111111111111111111111111111111111111111111111111111111111111111111111111111111111111111111111111111111111111111111111
                  6666666666666666666666666666666666666666666666666666666666666666666666666666666666666666666666666666666666666666666666666666666666666666666666666666666666666666666666666666666666666666666666666666666666666666666666666666666666666666666666666666666666666666666666666666666666666666666666666666666666666666666666666666666666666
                  0000000000000000000000000000000000000000000000000111111111111111111111111111111111111111111111111111111111111111111111111111111111111111111111111111111122222222222222222222222222222222222222222222222222222222222222222222222222222222222222222222222222222222223333333333333333333333333333333333333333333333333333333333333333333333333333333333333
                  5555555556666666666777777777788888888889999999999000000000011111111112222222222333333333344444444445555555555666666666677777777778888888888999999999900000000001111111111222222222233333333334444444444555555555566666666667777777777888888888899999999990000000000111111111122222222223333333333444444444455555555556666666666777777777788888
                  1234567890123456789012345678901234567890123456789012345678901234567890123456789012345678901234567890123456789012345678901234567890123456789012345678901234567890123456789012345678901234567890123456789012345678901234567890123456789012345678901234567890123456789012345678901234567890123456789012345678901234567890123456789012345678901234

CRS               ACCACCCAAGTATTGACTCACCCATCAACAACCGCTATGTATTTCGTACATTACTGCCAGCCACCATGAATATTGTACGGTACCATAAATACTTGACCACCTGTAGTACATAAAAACCCAATCCACATCAAAACCCCCTCCCCATGCTTACAAGCAAGTACAGCAATCAACCCTCAACTATCACACATCAACTGCAACTCCAAAGCCACCCCTCACCCACTAGGATACCAACAAACCTACCCACCCTTAACAGTACATAGTACATAAAGCCATTTACCGTACATAGCACATTACAGTCAAATCCCTTCTCGTCCCCATGGATGACCCCCCTCAG

clone 1 tooth 1                                                                 L16119............................................................................H16196
clone 2 tooth 1                                                                       .............T.............T................................................
clone 3 tooth 1                                                                       ......................T.....................................................
clone 4 tooth 1                                                                       ............................................................................
clone 5 tooth 2                                                                       ............................................................................
clone 6 tooth 2                                                                       ............................................................................
clone 7 tooth 2                                                                       ...............................................T............................
clone 1 tooth 1                                                                                                                                                L16181......T.T.T............T...............T..........T................H16249
clone 2 tooth 1                                                                                                                                                      ....................................T..............................
clone 3 tooth 1                                                                                                                                                      ...................................................................
clone 4 tooth 2                                                                                                                                                      ...................................................................
clone 5 tooth 2                                                                                                                                                      ........................................TT.T.......................
clone 6 tooth 2                                                                                                                                                      ...................................................................
clone 7 tooth 2                                                                                                                                                      ...................................................................
clone 8 tooth 2                                                                                                                                                      ...................................................................
clone 1 tooth 1                                                                                                                                                                            L16209.....................................................................................................................................G............H16356
clone 2 tooth 1                                                                                                                                                                                  ...............................G.............A.......................................................................................G............
clone 3 tooth 1                                                                                                                                                                                  .....................................................................................................................................G............
clone 4 tooth 1                                                                                                                                                                                  ................................................................................................T....................................G............
clone 5 tooth 1                                                                                                                                                                                  .............T.......................................................................................................................G............
clone 6 tooth 2                                                                                                                                                                                  .............T........T...........................................................................................................T..G............
clone 7 tooth 2                                                                                                                                                                                  .............T....................................................................................................................T..G............
clone 8 tooth 2                                                                                                                                                                                  .............................................................................................A.......................................G............
clone 9 tooth 2                                                                                                                                                                                  .....................................................................................................................................G............
```

Sample KA1 (RoIA)

```
                  1111111111111111111111111111111111111111111111111111111111111111111111111111111111111111111111111111111111111111111111111111111111111111111111111111111111111111111111111111111111111111111111111111111111111111111111111111111111111111111111111111111111111111111111111111111111111111111111111111111111111111111111111111111111111
                  6666666666666666666666666666666666666666666666666666666666666666666666666666666666666666666666666666666666666666666666666666666666666666666666666666666666666666666666666666666666666666666666666666666666666666666666666666666666666666666666666666666666666666666666666666666666666666666666666666666666666666666666666666666666666
                  0000000000000000000000000000000000000000000000000111111111111111111111111111111111111111111111111111111111111111111111111111111111111111111111111111111122222222222222222222222222222222222222222222222222222222222222222222222222222222222222222222222222222222223333333333333333333333333333333333333333333333333333333333333333333333333333333333333
                  5555555556666666666777777777788888888889999999999000000000011111111112222222222333333333344444444445555555555666666666677777777778888888888999999999900000000001111111111222222222233333333334444444444555555555566666666667777777777888888888899999999990000000000111111111122222222223333333333444444444455555555556666666666777777777788888
                  1234567890123456789012345678901234567890123456789012345678901234567890123456789012345678901234567890123456789012345678901234567890123456789012345678901234567890123456789012345678901234567890123456789012345678901234567890123456789012345678901234567890123456789012345678901234567890123456789012345678901234567890123456789012345678901234

CRS               ACCACCCAAGTATTGACTCACCCATCAACAACCGCTATGTATTTCGTACATTACTGCCAGCCACCATGAATATTGTACGGTACCATAAATACTTGACCACCTGTAGTACATAAAAACCCAATCCACATCAAAACCCCCTCCCCATGCTTACAAGCAAGTACAGCAATCAACCCTCAACTATCACACATCAACTGCAACTCCAAAGCCACCCCTCACCCACTAGGATACCAACAAACCTACCCACCCTTAACAGTACATAGTACATAAAGCCATTTACCGTACATAGCACATTACAGTCAAATCCCTTCTCGTCCCCATGGATGACCCCCCTCAG

clone 1 tooth 1                                                                 L16119.........................................................................T..H16196
clone 2 tooth 1                                                                       ............................................................................
clone 3 tooth 1                                                                       ............................................................................
clone 4 tooth 1                                                                       ............................................................................
clone 5 tooth 1                                                                       ............................................................................
clone 6 tooth 2                                                                       .....................................................T......................
clone 7 tooth 2                                                                       .......................C....................................................
clone 8 tooth 2                                                                       ............................................................................
clone 9 tooth 2                                                                       ............................................................................
clone 10 tooth 2                                                                      ............................................................................
clone 11 tooth 2                                                                      ............................................................................
clone 12 tooth 2                                                                      ............................................................................
clone 1 tooth 1                                                                                                                                                L16181........................................T..........................H16249
clone 2 tooth 1                                                                                                                                                      ....................................T..TT..T.......................
clone 3 tooth 1                                                                                                                                                      ......................A........A........T..........................
clone 4 tooth 1                                                                                                                                                      ........................................T..........................
clone 5 tooth 1                                                                                                                                                      ........................................T..........................
clone 6 tooth 2                                                                                                                                                      ........................................T..........................
clone 7 tooth 2                                                                                                                                                      ........................................T..........................
clone 8 tooth 2                                                                                                                                                      ........................................T..........................
clone 9 tooth 2                                                                                                                                                      ........................................T..........................
clone 10 tooth 2                                                                                                                                                     ...................................................................
clone 1 tooth 1                                                                                                                                                                            L16209............T.....................................................................................................................................H16356
clone 2 tooth 1                                                                                                                                                                                  ............T.....................................................................................................................................
clone 3 tooth 1                                                                                                                                                                                  ............T.....................................................................................................................................
clone 4 tooth 1                                                                                                                                                                                  ............T.....................................................................................................................................
clone 5 tooth 1                                                                                                                                                                                  ............T......................................................................G..............................................................
clone 6 tooth 1                                                                                                                                                                                  ............T..................................................................................C...................................................
```

```
clone 7 tooth 1     ............T..................................................................G..................................................................
clone 8 tooth 1     ............T.....................................................................................................................................

Sample KA2 (RoIA)
                    1111111111111111111111111111111111111111111111111111111111111111111111111111111111111111111111111111111111111111111111111111111111111111111111111111111111111111111111111111111111111111111111111111111111111111111111111111111111111111111111111111111111111111111111111111111111111111111111111111111111111111111111111111111111111111111111111111
                    6666666666666666666666666666666666666666666666666666666666666666666666666666666666666666666666666666666666666666666666666666666666666666666666666666666666666666666666666666666666666666666666666666666666666666666666666666666666666666666666666666666666666666666666666666666666666666666666666666666666666666666666666666666666666666666666
                    0000000000000000000000000000000000000000000000000111111111111111111111111111111111111111111111111111111111111111111111111111111111111111111111111111122222222222222222222222222222222222222222222222222222222222222222222222222222222222222222222222222222222222222222222222222222222222222222222233333333333333333333333333333333333333333333333333333333333333333333333333333333333
                    5555555556666666666777777777788888888889999999999000000000011111111112222222222333333333344444444445555555555666666666677777777778888888888999999999900000000001111111111222222222233333333334444444444555555555566666666667777777777888888888899999999990000000000111111111122222222223333333333444444444455555555556666666666777777777788888
                    1234567890123456789012345678901234567890123456789012345678901234567890123456789012345678901234567890123456789012345678901234567890123456789012345678901234567890123456789012345678901234567890123456789012345678901234567890123456789012345678901234567890123456789012345678901234567890123456789012345678901234567890123456789012345678901234

CRS                 ACCACCCAAGTATTGACTCACCCATCAACAACCGCTATGTATTTCGTACATTACTGCCAGCCACCATGAATATTGTACGGTACCATAAATACTTGACCACCTGTAGTACATAAAAACCCAATCCACATCAAAACCCCCTCCCCATGCTTACAAGCAAGTACAGCAATCAACCCTCAACTATCACACATCAACTGCAACTCCAAAGCCACCCCTCACCCACTAGGATACCAACAAACCTACCCACCCTTAACAGTACATAGTACATAAAGCCATTTACCGTACATAGCACATTACAGTCAAATCCCTTCTCGTCCCCATGGATGACCCCCCTCAG

clone 1 tooth 1     L16119...........................A................................................H16196
clone 2 tooth 1     .........A..................................................................
clone 3 tooth 1     ...........................A................................................
clone 4 tooth 1     ...........................A..........G.....................................
clone 5 tooth 2     ...........................A................................................
clone 6 tooth 2     ...........................A................................................
clone 7 tooth 2     ...........................A................................................
clone 8 tooth 2     ...........................A................................................
clone 9 tooth 2     ...........................A................................................
clone 1 tooth 1     L16181.........Y........Y.......Y.............T.....................Y..T.H16249
clone 2 tooth 1     ........................................T........................T.
clone 3 tooth 1     ........................................T........................T.
clone 4 tooth 1     ............................T..T........T........................T.
clone 5 tooth 1     ..Y...............T.....................T.......A.............T..T.
clone 1 tooth 1     L16209.T..........TT.T......................T.................T.............................T.........T.............T..................................TH16356
clone 2 tooth 1     .T..........TT.T......................T..............C..T.............................T.........T.............T..................................T
clone 3 tooth 1     .T..........TT.T......................T.................T.............................T.........T.............T..................................T
clone 4 tooth 1     .T..........TT.T......................T.................T.............................T.........T.............T..................................T
clone 5 tooth 1     .T..........TT.T......................T.......................................................................T..................................T
clone 6 tooth 1     .T..........TT.T......................T.......................................................................T..................................T
clone 7 tooth 2     .............T........................T......................C................................................T..................................T
clone 8 tooth 2     .............T............T...........T.......................................................................TT.................................T
clone 9 tooth 2     .............T............T...........T.......................................................................TT.................................T

Sample G1 (RoIA)
                    1111111111111111111111111111111111111111111111111111111111111111111111111111111111111111111111111111111111111111111111111111111111111111111111111111111111111111111111111111111111111111111111111111111111111111111111111111111111111111111111111111111111111111111111111111111111111111111111111111111111111111111111111111111111111111111111
                    6666666666666666666666666666666666666666666666666666666666666666666666666666666666666666666666666666666666666666666666666666666666666666666666666666666666666666666666666666666666666666666666666666666666666666666666666666666666666666666666666666666666666666666666666666666666666666666666666666666666666666666666666666666666666666666666
                    0000000000000000000000000000000000000000000000000111111111111111111111111111111111111111111111111111111111111111111111111111111111111111111111111111122222222222222222222222222222222222222222222222222222222222222222222222222222222222222222222222222222222222222222222222222222222222222222222233333333333333333333333333333333333333333333333333333333333333333333333333333333333
                    5555555556666666666777777777788888888889999999999000000000011111111112222222222333333333344444444445555555555666666666677777777778888888888999999999900000000001111111111222222222233333333334444444444555555555566666666667777777777888888888899999999990000000000111111111122222222223333333333444444444455555555556666666666777777777788888
                    1234567890123456789012345678901234567890123456789012345678901234567890123456789012345678901234567890123456789012345678901234567890123456789012345678901234567890123456789012345678901234567890123456789012345678901234567890123456789012345678901234567890123456789012345678901234567890123456789012345678901234567890123456789012345678901234

CRS                 ACCACCCAAGTATTGACTCACCCATCAACAACCGCTATGTATTTCGTACATTACTGCCAGCCACCATGAATATTGTACGGTACCATAAATACTTGACCACCTGTAGTACATAAAAACCCAATCCACATCAAAACCCCCTCCCCATGCTTACAAGCAAGTACAGCAATCAACCCTCAACTATCACACATCAACTGCAACTCCAAAGCCACCCCTCACCCACTAGGATACCAACAAACCTACCCACCCTTAACAGTACATAGTACATAAAGCCATTTACCGTACATAGCACATTACAGTCAAATCCCTTCTCGTCCCCATGGATGACCCCCCTCAG

clone 1 tooth 1     L16119.........AA..........................C......................................H16196
clone 2 tooth 1     .........A..................................................................
clone 3 tooth 1     .........AA..........................C......................................
clone 4 tooth 1     .........A..............................................T...................
clone 5 tooth 1     .........A..................................................................
clone 6 tooth 2     ............................................................................
clone 7 tooth 2     ............................................................................
clone 8 tooth 2     .........A..................................................................
clone 9 tooth 2     .........A..................................................................
clone 10 tooth 2    .........A..................................................................
clone 11 tooth 2    .........A..................................................................
clone 12 tooth 2    .........Y.Y................................................................
clone 13 tooth 2    .........A..................................................................
clone 1 tooth 1     L16181...........T.................T..T...T...TT.........................H16249
clone 2 tooth 1     ...................................................................
clone 3 tooth 1     ..........................A........................................
clone 4 tooth 1     ...................................................................
clone 5 tooth 1     ...................................................................
clone 1 tooth 2     L16209..............................................................................................C...................................................H16356
clone 2 tooth 2     ..............................................................................................C...................................................
clone 3 tooth 2     ..............................................................................................C...................................................
clone 4 tooth 2     ..............................................................................................C...................................................
clone 5 tooth 2     ..............................................................................................C...................................................
clone 1 tooth 1     L16249......................................................C............H16317
clone 2 tooth 1     ......................................................C............
clone 3 tooth 1     T......T.T.TT.........................................C............
clone 4 tooth 1     ......................................................C............
clone 5 tooth 1     ......................................................C............

Sample G2 (RoIA)
                    1111111111111111111111111111111111111111111111111111111111111111111111111111111111111111111111111111111111111111111111111111111111111111111111111111111111111111111111111111111111111111111111111111111111111111111111111111111111111111111111111111111111111111111111111111111111111111111111111111111111111111111111111111111111111111111111
                    6666666666666666666666666666666666666666666666666666666666666666666666666666666666666666666666666666666666666666666666666666666666666666666666666666666666666666666666666666666666666666666666666666666666666666666666666666666666666666666666666666666666666666666666666666666666666666666666666666666666666666666666666666666666666666666666
                    0000000000000000000000000000000000000000000000000111111111111111111111111111111111111111111111111111111111111111111111111111111111111111111111111111122222222222222222222222222222222222222222222222222222222222222222222222222222222222222222222222222222222222222222222222222222222222222222222233333333333333333333333333333333333333333333333333333333333333333333333333333333333
                    5555555556666666666777777777788888888889999999999000000000011111111112222222222333333333344444444445555555555666666666677777777778888888888999999999900000000001111111111222222222233333333334444444444555555555566666666667777777777888888888899999999990000000000111111111122222222223333333333444444444455555555556666666666777777777788888
                    1234567890123456789012345678901234567890123456789012345678901234567890123456789012345678901234567890123456789012345678901234567890123456789012345678901234567890123456789012345678901234567890123456789012345678901234567890123456789012345678901234567890123456789012345678901234567890123456789012345678901234567890123456789012345678901234

CRS                 ACCACCCAAGTATTGACTCACCCATCAACAACCGCTATGTATTTCGTACATTACTGCCAGCCACCATGAATATTGTACGGTACCATAAATACTTGACCACCTGTAGTACATAAAAACCCAATCCACATCAAAACCCCCTCCCCATGCTTACAAGCAAGTACAGCAATCAACCCTCAACTATCACACATCAACTGCAACTCCAAAGCCACCCCTCACCCACTAGGATACCAACAAACCTACCCACCCTTAACAGTACATAGTACATAAAGCCATTTACCGTACATAGCACATTACAGTCAAATCCCTTCTCGTCCCCATGGATGACCCCCCTCAG

clone 1 tooth 1     L16119.....A......................................................................H16196
clone 2 tooth 1     ............................................................................
clone 3 tooth 1     ..........A.................................................................
clone 4 tooth 1     ............................................................................
clone 5 tooth 1     ............................................................................
clone 6 tooth 2     ............................................................................
clone 7 tooth 2     ............................................................................
clone 8 tooth 2     ............................................................................
clone 9 tooth 2     .............T..............................................................
clone 10 tooth 2    ............................................................................
clone 1 tooth 1     L16181....T..CT....................T...........................T.........H16249
clone 2 tooth 1     ...................................................................
clone 3 tooth 1     .......................................T...........................
clone 4 tooth 1     .................................................................T.
clone 5 tooth 1     ...................................................................
clone 1 tooth 1     L16209...................................................T..............................................................................................H16356
clone 2 tooth 1     ......................................T.......T......C......T...................................T.................................................
clone 3 tooth 1     ..............................................T..T...C......T.....................................................................................
clone 4 tooth 1     ..............................................T......C......T.....................................................................................
clone 5 tooth 1     ..............................................T.G....C......T.....................................................................................
clone 6 tooth 2     ..............................................T......C......T.....................................................................................
clone 7 tooth 2     ....T.........................................T......C......T.....................................................................................
clone 8 tooth 2     ....T.........................................T......C......T.....................................................................................
clone 9 tooth 2     ..............................................T......C......T.....................................................................................
clone 10 tooth 2    ....T.........................................T......C......T.....................................................................................
clone 11 tooth 1    ..............................................T......C......T.....................................................................................
clone 12 tooth 1    ...............................G..............T......C......T.....................................................................................
clone 13 tooth 1    ..............................................T......C......T.....................................................................................
clone 14 tooth 1    ..............................................T......C..T...T...................T....T............................................................
clone 15 tooth 1    ..............................................T......C......T.....................................................................................
clone 16 tooth 1    ..............................................T......C......T.....................................................................................

Sample C1 (ME)
                    1111111111111111111111111111111111111111111111111111111111111111111111111111111111111111111111111111111111111111111111111111111111111111111111111111111111111111111111111111111111111111111111111111111111111111111111111111111111111111111111111111111111111111111111111111111111111111111111111111111111111111111111111111111111111111111111
                    6666666666666666666666666666666666666666666666666666666666666666666666666666666666666666666666666666666666666666666666666666666666666666666666666666666666666666666666666666666666666666666666666666666666666666666666666666666666666666666666666666666666666666666666666666666666666666666666666666666666666666666666666666666666666666666666
                    0000000000000000000000000000000000000000000000000111111111111111111111111111111111111111111111111111111111111111111111111111111111111111111111111111122222222222222222222222222222222222222222222222222222222222222222222222222222222222222222222222222222222222222222222222222222222222222222222233333333333333333333333333333333333333333333333333333333333333333333333333333333333
                    5555555556666666666777777777788888888889999999999000000000011111111112222222222333333333344444444445555555555666666666677777777778888888888999999999900000000001111111111222222222233333333334444444444555555555566666666667777777777888888888899999999990000000000111111111122222222223333333333444444444455555555556666666666777777777788888
                    1234567890123456789012345678901234567890123456789012345678901234567890123456789012345678901234567890123456789012345678901234567890123456789012345678901234567890123456789012345678901234567890123456789012345678901234567890123456789012345678901234567890123456789012345678901234567890123456789012345678901234567890123456789012345678901234

CRS                 ACCACCCAAGTATTGACTCACCCATCAACAACCGCTATGTATTTCGTACATTACTGCCAGCCACCATGAATATTGTACGGTACCATAAATACTTGACCACCTGTAGTACATAAAAACCCAATCCACATCAAAACCCCCTCCCCATGCTTACAAGCAAGTACAGCAATCAACCCTCAACTATCACACATCAACTGCAACTCCAAAGCCACCCCTCACCCACTAGGATACCAACAAACCTACCCACCCTTAACAGTACATAGTACATAAAGCCATTTACCGTACATAGCACATTACAGTCAAATCCCTTCTCGTCCCCATGGATGACCCCCCTCAG

clone 1 tooth 1     L16119..........A.................................................................H16196
clone 2 tooth 1     ..................................C.........................................
clone 3 tooth 1     ............................................................................
clone 4 tooth 1     ............................................................................
clone 5 tooth 1     ............................................................................
clone 6 tooth 1     ..........A.................................................................
clone 7 tooth 2     ............................................................................
clone 8 tooth 2     ............................................................................
clone 9 tooth 2     ............................................................................
clone 10 tooth 2    ............................................................................
clone 1 tooth 1     L16181...................................................................H16249
clone 2 tooth 1     ...........................T..T...T..T...T.........................
clone 3 tooth 1     ............................................T...T.T................
clone 4 tooth 1     ..................................................T................
clone 5 tooth 1     ..................................................T................
clone 6 tooth 2     ...................................................................
clone 7 tooth 2     ..................................................T.T..............
clone 8 tooth 2     ..................................................T.T..............
clone 9 tooth 2     ....T..............................................................
clone 10 tooth 1    .....T.............................................................
clone 1 tooth 1     L16209..................................................................................................................................................H16356
clone 2 tooth 1     ..................................................................................................................................................
clone 3 tooth 1     ......Y...........................................................................................................................................
clone 4 tooth 1     ..................................................................................................................................................
clone 5 tooth 1     ..................................................................................................................................................
clone 6 tooth 2     ..................................................................................................................................................
```

```
clone 7 tooth 2                                                                                                                                                                                         ..................................................................................................................................................
clone 8 tooth 2                                                                                                                                                                                         ..................................................................................................................................................
clone 9 tooth 2                                                                                                                                                                                         ...................................................T..T.TT...........................................................TT...............T...........
clone 10 tooth 2                                                                                                                                                                                        ..................................................................................................................................................
```

Sample C2 (ME)

```
                        1111111111111111111111111111111111111111111111111111111111111111111111111111111111111111111111111111111111111111111111111111111111111111111111111111111111111111111111111111111111111111111111111111111111111111111111111111111111111111111111111111111111111111111111111111111111111111111111111111111111111111111111111111111111
                        6666666666666666666666666666666666666666666666666666666666666666666666666666666666666666666666666666666666666666666666666666666666666666666666666666666666666666666666666666666666666666666666666666666666666666666666666666666666666666666666666666666666666666666666666666666666666666666666666666666666666666666666666666666666
                        0000000000000000000000000000000000000000000000000111111111111111111111111111111111111111111111111111111111111111111111111111111111111111111111111111111112222222222222222222222222222222222222222222222222222222222222222222222222222222222222222222222222222222222222222222222222233333333333333333333333333333333333333333333333333333333333333333333333333333333333333333
                        5555555556666666666777777777788888888889999999999000000000011111111112222222222333333333344444444445555555555666666666677777777778888888888999999999900000000001111111111222222222233333333334444444444555555555566666666667777777777888888888899999999990000000000111111111122222222223333333333444444444455555555556666666666777777777788888
                        1234567890123456789012345678901234567890123456789012345678901234567890123456789012345678901234567890123456789012345678901234567890123456789012345678901234567890123456789012345678901234567890123456789012345678901234567890123456789012345678901234567890123456789012345678901234567890123456789012345678901234567890123456789012345678901234

CRS                     ACCACCCAAGTATTGACTCACCCATCAACAACCGCTATGTATTTCGTACATTACTGCCAGCCACCATGAATATTGTACGGTACCATAAATACTTGACCACCTGTAGTACATAAAAACCCAATCCACATCAAAACCCCCTCCCCATGCTTACAAGCAAGTACAGCAATCAACCCTCAACTATCACACATCAACTGCAACTCCAAAGCCACCCCTCACCCACTAGGATACCAACAAACCTACCCACCCTTAACAGTACATAGTACATAAAGCCATTTACCGTACATAGCACATTACAGTCAAATCCCTTCTCGTCCCCATGGATGACCCCCCTCAG

clone 1 tooth 1                                                                L16119............................................................................H16196
clone 2 tooth 1                                                                      .....A......................................................................
clone 3 tooth 1                                                                      ............................................................................
clone 4 tooth 1                                                                      ............................................................................
clone 5 tooth 1                                                                      ............................................................................
clone 6 tooth 2                                                                      ..............Y.............................................................
clone 7 tooth 2                                                                      .......................................T.........T..........................
clone 8 tooth 2                                                                      ...........................T..........................T.....................
clone 9 tooth 2                                                                      ...............................................T......T.....................
clone 10 tooth 2                                                                     ............................................................................
clone 1 tooth 1                                                                                                                                      L16181...................................................................H16249
clone 2 tooth 1                                                                                                                                            ...................................................................
clone 3 tooth 1                                                                                                                                            ...................................................................
clone 4 tooth 1                                                                                                                                            ...................................Y...............................
clone 5 tooth 1                                                                                                                                            ...................................................................
clone 1 tooth 1                                                                                                                                                                              L16209.............T..................................................................T............................A....................................H16356
clone 2 tooth 1                                                                                                                                                                                    ..................................................................................................................................................
clone 3 tooth 1                                                                                                                                                                                    .............T..................................................................T............................A....................................
clone 4 tooth 1                                                                                                                                                                                    ..................................................................................................................................................
clone 5 tooth 1                                                                                                                                                                                    ..................................................................................................................................................
clone 6 tooth 1                                                                                                                                                                                    ..................................................................................................................................................
clone 7 tooth 1                                                                                                                                                                                    ..................................................................................................................................................
clone 8 tooth 1                                                                                                                                                                                    ..................................................................................................................................................
clone 9 tooth 2                                                                                                                                                                                    ...............T......T...T..T...................TTT.....T......................T.T.TT................................T...T.......................
clone 10 tooth 2                                                                                                                                                                                   .............................T........................T...T.....................TTT...T...........................................................
clone 11 tooth 2                                                                                                                                                                                   .............T....................................T...............................................................................................
clone 12 tooth 2                                                                                                                                                                                   ...........T.T....T.....T.........................TT..T.....T.......TT..T.......TTT..TT.......................TT.....T....................T.......
clone 13 tooth 2                                                                                                                                                                                   .............T....................................T...............................................................................................
clone 14 tooth 2                                                                                                                                                                                   .............................................................................................................A....................................
clone 15 tooth 2                                                                                                                                                                                   ...........T.T....T.....T.........................TT..T.....T.......TT..T.......TTT..TT.......................TT.....T....................T.......
clone 16 tooth 2                                                                                                                                                                                   ...............T......T...T..T...................TTTT....T......................T.T.TT................................T...T.......................
```

Sample C3 (ME)

```
                        1111111111111111111111111111111111111111111111111111111111111111111111111111111111111111111111111111111111111111111111111111111111111111111111111111111111111111111111111111111111111111111111111111111111111111111111111111111111111111111111111111111111111111111111111111111111111111111111111111111111111111111111111111111111
                        6666666666666666666666666666666666666666666666666666666666666666666666666666666666666666666666666666666666666666666666666666666666666666666666666666666666666666666666666666666666666666666666666666666666666666666666666666666666666666666666666666666666666666666666666666666666666666666666666666666666666666666666666666666666
                        0000000000000000000000000000000000000000000000000111111111111111111111111111111111111111111111111111111111111111111111111111111111111111111111111111111112222222222222222222222222222222222222222222222222222222222222222222222222222222222222222222222222222222222222222222222222233333333333333333333333333333333333333333333333333333333333333333333333333333333333333333
                        5555555556666666666777777777788888888889999999999000000000011111111112222222222333333333344444444445555555555666666666677777777778888888888999999999900000000001111111111222222222233333333334444444444555555555566666666667777777777888888888899999999990000000000111111111122222222223333333333444444444455555555556666666666777777777788888
                        1234567890123456789012345678901234567890123456789012345678901234567890123456789012345678901234567890123456789012345678901234567890123456789012345678901234567890123456789012345678901234567890123456789012345678901234567890123456789012345678901234567890123456789012345678901234567890123456789012345678901234567890123456789012345678901234

CRS                     ACCACCCAAGTATTGACTCACCCATCAACAACCGCTATGTATTTCGTACATTACTGCCAGCCACCATGAATATTGTACGGTACCATAAATACTTGACCACCTGTAGTACATAAAAACCCAATCCACATCAAAACCCCCTCCCCATGCTTACAAGCAAGTACAGCAATCAACCCTCAACTATCACACATCAACTGCAACTCCAAAGCCACCCCTCACCCACTAGGATACCAACAAACCTACCCACCCTTAACAGTACATAGTACATAAAGCCATTTACCGTACATAGCACATTACAGTCAAATCCCTTCTCGTCCCCATGGATGACCCCCCTCAG

clone 1 tooth 1                                                                L16119............................................................................H16196
clone 2 tooth 1                                                                      ............................................................................
clone 3 tooth 1                                                                      ............................................................................
clone 4 tooth 1                                                                      ............................................................................
clone 5 tooth 1                                                                      ............................................................................
clone 6 tooth 1                                                                      ...............................T............................................
clone 7 tooth 1                                                                      ...............................T............................................
clone 8 tooth 1                                                                      ...............................T............................................
clone 9 tooth 2                                                                      ....................................A.......................................
clone 10 tooth 2                                                                     ...........................Y.............Y......Y...........................
clone 11 tooth 2                                                                     ............................................................................
clone 12 tooth 2                                                                     .....A...................A..................................................
clone 13 tooth 2                                                                     ............................................................................
clone 14 tooth 2                                                                     ............................................................................
clone 15 tooth 2                                                                     ..............T.............................................................
clone 16 tooth 2                                                                     ............................................................................
clone 1 tooth 2                                                                                                                                      L16181....T...........................T........................T.........H16249
clone 2 tooth 2                                                                                                                                            ...................................................................
clone 3 tooth 2                                                                                                                                            ...................................................................
clone 4 tooth 2                                                                                                                                            ...................................................................
clone 5 tooth 2                                                                                                                                            ...................................................................
clone 6 tooth 2                                                                                                                                            ...T.TT..T...............................................T.........
clone 7 tooth 2                                                                                                                                            ...................................................................
clone 8 tooth 2                                                                                                                                            ...................................................................
clone 9 tooth 2                                                                                                                                            ...................................................................
clone 1 tooth 2                                                                                                                                                                              L16209................................................................................................................................................T.H16356
clone 2 tooth 2                                                                                                                                                                                    ................................................................................................................................................T.
clone 3 tooth 2                                                                                                                                                                                    ................................................................................................................................................T.
clone 4 tooth 2                                                                                                                                                                                    ........................................................................A.......................................................................T.
clone 5 tooth 2                                                                                                                                                                                    ................................................................................................................................................T.
clone 6 tooth 2                                                                                                                                                                                    ................................................................................................................................................T.
clone 7 tooth 2                                                                                                                                                                                    ................................................................................................................................................T.
clone 8 tooth 2                                                                                                                                                                                    .......................................................................................................................A......A.........A.......T.
clone 9 tooth 1                                                                                                                                                                                    ........................................................................A..................T....................................................T.
clone 10 tooth 1                                                                                                                                                                                   ................................................................................................................................................T.
clone 11 tooth 1                                                                                                                                                                                   ...................TT.....T.....T..........T..T..T....TT.................................T.........T...........T................................T.
```

Sample C4 (ME)

```
                        1111111111111111111111111111111111111111111111111111111111111111111111111111111111111111111111111111111111111111111111111111111111111111111111111111111111111111111111111111111111111111111111111111111111111111111111111111111111111111111111111111111111111111111111111111111111111111111111111111111111111111111111111111111111
                        6666666666666666666666666666666666666666666666666666666666666666666666666666666666666666666666666666666666666666666666666666666666666666666666666666666666666666666666666666666666666666666666666666666666666666666666666666666666666666666666666666666666666666666666666666666666666666666666666666666666666666666666666666666666
                        0000000000000000000000000000000000000000000000000111111111111111111111111111111111111111111111111111111111111111111111111111111111111111111111111111111112222222222222222222222222222222222222222222222222222222222222222222222222222222222222222222222222222222222222222222222222233333333333333333333333333333333333333333333333333333333333333333333333333333333333333333
                        5555555556666666666777777777788888888889999999999000000000011111111112222222222333333333344444444445555555555666666666677777777778888888888999999999900000000001111111111222222222233333333334444444444555555555566666666667777777777888888888899999999990000000000111111111122222222223333333333444444444455555555556666666666777777777788888
                        1234567890123456789012345678901234567890123456789012345678901234567890123456789012345678901234567890123456789012345678901234567890123456789012345678901234567890123456789012345678901234567890123456789012345678901234567890123456789012345678901234567890123456789012345678901234567890123456789012345678901234567890123456789012345678901234

CRS                     ACCACCCAAGTATTGACTCACCCATCAACAACCGCTATGTATTTCGTACATTACTGCCAGCCACCATGAATATTGTACGGTACCATAAATACTTGACCACCTGTAGTACATAAAAACCCAATCCACATCAAAACCCCCTCCCCATGCTTACAAGCAAGTACAGCAATCAACCCTCAACTATCACACATCAACTGCAACTCCAAAGCCACCCCTCACCCACTAGGATACCAACAAACCTACCCACCCTTAACAGTACATAGTACATAAAGCCATTTACCGTACATAGCACATTACAGTCAAATCCCTTCTCGTCCCCATGGATGACCCCCCTCAG

clone 1 tooth 1                                                                L16119...........................T...............................T....T...TC......H16196
clone 2 tooth 1                                                                      .....................................................................C......
clone 3 tooth 1                                                                      ...............................................T............G........C......
clone 4 tooth 1                                                                      .....................................................................-......
clone 5 tooth 1                                                                      .....................................................................C......
clone 6 tooth 1                                                                      .....................................................................C......
clone 7 tooth 2                                                                      .....................................................................C......
clone 8 tooth 2                                                                      .............................Y.......................................C......
clone 9 tooth 2                                                                      .....................................................................C......
clone 10 tooth 2                                                                     .....................................................................C......
clone 11 tooth 2                                                                     .....................................................................C......
clone 1 tooth 1                                                                                                                                      L16181...................................................................H16249
clone 2 tooth 1                                                                                                                                            .......-...........................................................
clone 3 tooth 2                                                                                                                                            .C.....C...........................................................
clone 4 tooth 2                                                                                                                                            .....T...TT........T...T........T..................................
clone 1 tooth 1                                                                                                                                                                              L16209..................................................................................................................................................H16356
clone 2 tooth 1                                                                                                                                                                                    ..................................................................................................................................................
clone 3 tooth 1                                                                                                                                                                                    ..................................................................................................................................................
clone 4 tooth 1                                                                                                                                                                                    ..................................................................................................................................................
clone 5 tooth 1                                                                                                                                                                                    ..................................................................................................................................................
clone 6 tooth 2                                                                                                                                                                                    ..................................................................................................................................................
clone 7 tooth 2                                                                                                                                                                                    ........................T.......T..T.....TG...........T..T..........T............T..T.............................................................
clone 8 tooth 2                                                                                                                                                                                    ........................T.......T..T.....TG.....G.....T..T..........T............T..T.............................................................
clone 9 tooth 2                                                                                                                                                                                    ..................................................................................................................................................
clone 10 tooth 2                                                                                                                                                                                   ..................................................................................................................................................
```

Sample C5 (ME)

```
                        1111111111111111111111111111111111111111111111111111111111111111111111111111111111111111111111111111111111111111111111111111111111111111111111111111111111111111111111111111111111111111111111111111111111111111111111111111111111111111111111111111111111111111111111111111111111111111111111111111111111111111111111111111111111
                        6666666666666666666666666666666666666666666666666666666666666666666666666666666666666666666666666666666666666666666666666666666666666666666666666666666666666666666666666666666666666666666666666666666666666666666666666666666666666666666666666666666666666666666666666666666666666666666666666666666666666666666666666666666666
                        0000000000000000000000000000000000000000000000000111111111111111111111111111111111111111111111111111111111111111111111111111111111111111111111111111111112222222222222222222222222222222222222222222222222222222222222222222222222222222222222222222222222222222222222222222222222233333333333333333333333333333333333333333333333333333333333333333333333333333333333333333
                        5555555556666666666777777777788888888889999999999000000000011111111112222222222333333333344444444445555555555666666666677777777778888888888999999999900000000001111111111222222222233333333334444444444555555555566666666667777777777888888888899999999990000000000111111111122222222223333333333444444444455555555556666666666777777777788888
                        1234567890123456789012345678901234567890123456789012345678901234567890123456789012345678901234567890123456789012345678901234567890123456789012345678901234567890123456789012345678901234567890123456789012345678901234567890123456789012345678901234567890123456789012345678901234567890123456789012345678901234567890123456789012345678901234

CRS                     ACCACCCAAGTATTGACTCACCCATCAACAACCGCTATGTATTTCGTACATTACTGCCAGCCACCATGAATATTGTACGGTACCATAAATACTTGACCACCTGTAGTACATAAAAACCCAATCCACATCAAAACCCCCTCCCCATGCTTACAAGCAAGTACAGCAATCAACCCTCAACTATCACACATCAACTGCAACTCCAAAGCCACCCCTCACCCACTAGGATACCAACAAACCTACCCACCCTTAACAGTACATAGTACATAAAGCCATTTACCGTACATAGCACATTACAGTCAAATCCCTTCTCGTCCCCATGGATGACCCCCCTCAG

clone 1 tooth 1   L16050...........................................................................C...H1630
clone 2 tooth 1         ......................Y.........................Y..........................C...
clone 3 tooth 1         ...............................................................................
clone 4 tooth 1         ...............................................................................
clone 5 tooth 1         ...............................................................................
clone 1 tooth 1                                                                L16119......C.................................G...................................H16196
clone 2 tooth 1                                                                      ......C.....................................................................
clone 3 tooth 1                                                                      ......C.....................................................................
clone 4 tooth 1                                                                      ......C...........................................................T.........
clone 5 tooth 2                                                                      ......C.....................................................................
clone 6 tooth 2                                                                      ......C.....................................................................
clone 7 tooth 2                                                                      ......C.....................................................................
clone 8 tooth 2                                                                      ......C.....................................................................
```

```
clone 9 tooth 2   ......C.......Y...........................................Y.................
clone 1 tooth 2   L16181..T................................................................H16249
clone 2 tooth 2   ...................................................................
clone 3 tooth 2   ...................................................................
clone 4 tooth 2   ..............................................................A....
clone 5 tooth 2   ..............................................................A....
clone 1 tooth 2   L16209...........................................................G....A.................................................................................H16356
clone 2 tooth 2   .................................................T................................................................................................
clone 3 tooth 2   .................................................T................................................................................................
clone 3 tooth 2   .................................................T................................................................................................
clone 5 tooth 2   ....................................................T...TT................................................................T.......................
clone 6 tooth 2   .................................................T................................................................................................
clone 7 tooth 2   ..................................................................................................................................................
clone 8 tooth 2   .................................................T................................................................................................
clone 9 tooth 2   ......................................................T...........................................................................................
clone 1 tooth 1   L16249...................................................................H16317
clone 2 tooth 1   ........T..................................T.......................
clone 3 tooth 1   ...............T...................................................
clone 4 tooth 1   ..............T....................................................
clone 5 tooth 1   ..............T....................................................
clone 6 tooth 1   ...................................................................
clone 7 tooth 1   .........T.................................T.......................
clone 8 tooth 1   ................T..................................................
```

Sample C6 (ME)

```
11111111111111111111111111111111111111111111111111111111111111111111111111111111111111111111111111111111111111111111111111111111111111111111111111111111111111111111111111111111111111111111111111111111111111111111111111111111111111111111111111111111111111111111111111111111111111111111111111111111111111111111111111111111111111111111111
66666666666666666666666666666666666666666666666666666666666666666666666666666666666666666666666666666666666666666666666666666666666666666666666666666666666666666666666666666666666666666666666666666666666666666666666666666666666666666666666666666666666666666666666666666666666666666666666666666666666666666666666666666666666666666666
0000000000000000000000000000000000000000000000000111111111111111111111111111111111111111111111111111111111111111111111111111111111111111111111111111111111222222222222222222222222222222222222222222222222222222222222222222222222222222222222222222222222222222222222222222233333333333333333333333333333333333333333333333333333333333333333333333333333333333
5555555556666666666777777777788888888889999999999000000000011111111112222222222333333333344444444445555555555666666666677777777778888888888999999999900000000001111111111222222222233333333334444444444555555555566666666667777777777888888888899999999990000000000111111111122222222223333333333444444444455555555556666666666777777777788888
1234567890123456789012345678901234567890123456789012345678901234567890123456789012345678901234567890123456789012345678901234567890123456789012345678901234567890123456789012345678901234567890123456789012345678901234567890123456789012345678901234567890123456789012345678901234567890123456789012345678901234567890123456789012345678901234

CRS   ACCACCCAAGTATTGACTCACCCATCAACAACCGCTATGTATTTCGTACATTACTGCCAGCCACCATGAATATTGTACGGTACCATAAATACTTGACCACCTGTAGTACATAAAAACCCAATCCACATCAAAACCCCCTCCCCATGCTTACAAGCAAGTACAGCAATCAACCCTCAACTATCACACATCAACTGCAACTCCAAAGCCACCCCTCACCCACTAGGATACCAACAAACCTACCCACCCTTAACAGTACATAGTACATAAAGCCATTTACCGTACATAGCACATTACAGTCAAATCCCTTCTCGTCCCCATGGATGACCCCCCTCAG

clone 1 tooth 1    L16119....................................A.....G.................................H16196
clone 2 tooth 1    ..........................................G.................................
clone 3 tooth 1    ..........................................G.................................
clone 4 tooth 1    ..........................................G.................................
clone 5 tooth 1    ..........................................G.................................
clone 6 tooth 2    ..........................................G.................................
clone 7 tooth 2    ..........................................G........................T........
clone 8 tooth 2    .............T............................G...........T..T..T.....T...T.....
clone 9 tooth 2    .....A.......................G............G.......A.........................
clone 10 tooth 2   ..........................................G.................................
clone 1 tooth 1    L16181...................................................................H16249
clone 2 tooth 1    ...................................................................
clone 3 tooth 1    ...................................................................
clone 4 tooth 1    ...................................................................
clone 5 tooth 1    ..............................................................Y....
clone 6 tooth 2    ...................................................................
clone 7 tooth 2    ...................................................................
clone 8 tooth 2    ...................................................................
clone 9 tooth 2    ...................................................................
clone 10 tooth 2   ...................................................................
clone 1 tooth 1    L16209..................................................................................................................................................H16356
clone 2 tooth 1    ..................................................................................................................................................
clone 3 tooth 1    ................................................................................T.................................................................
clone 4 tooth 1    ......................T.T......................................................................................TT................T................
clone 5 tooth 1    .............T..T.....T.............................T.T................................................T..........................................
clone 6 tooth 2    ..................................................................................................................................................
clone 7 tooth 2    ............T.........................................................................................................................T...........
clone 8 tooth 2    ...A........................................................................................T................A....................................
clone 9 tooth 2    ...Y...........................................................Y.............................................A....................................
clone 10 tooth 2   ...............................................................A....................................A........A....................................
```

Sample C7 (ME)

```
11111111111111111111111111111111111111111111111111111111111111111111111111111111111111111111111111111111111111111111111111111111111111111111111111111111111111111111111111111111111111111111111111111111111111111111111111111111111111111111111111111111111111111111111111111111111111111111111111111111111111111111111111111111111111111111
66666666666666666666666666666666666666666666666666666666666666666666666666666666666666666666666666666666666666666666666666666666666666666666666666666666666666666666666666666666666666666666666666666666666666666666666666666666666666666666666666666666666666666666666666666666666666666666666666666666666666666666666666666666666666666666
0000000000000000000000000000000000000000000000000111111111111111111111111111111111111111111111111111111111111111111111111111111111111111111111111111111111222222222222222222222222222222222222222222222222222222222222222222222222222222222222222222222222222222222222222222233333333333333333333333333333333333333333333333333333333333333333333333333333333333
5555555556666666666777777777788888888889999999999000000000011111111112222222222333333333344444444445555555555666666666677777777778888888888999999999900000000001111111111222222222233333333334444444444555555555566666666667777777777888888888899999999990000000000111111111122222222223333333333444444444455555555556666666666777777777788888
1234567890123456789012345678901234567890123456789012345678901234567890123456789012345678901234567890123456789012345678901234567890123456789012345678901234567890123456789012345678901234567890123456789012345678901234567890123456789012345678901234567890123456789012345678901234567890123456789012345678901234567890123456789012345678901234

CRS   ACCACCCAAGTATTGACTCACCCATCAACAACCGCTATGTATTTCGTACATTACTGCCAGCCACCATGAATATTGTACGGTACCATAAATACTTGACCACCTGTAGTACATAAAAACCCAATCCACATCAAAACCCCCTCCCCATGCTTACAAGCAAGTACAGCAATCAACCCTCAACTATCACACATCAACTGCAACTCCAAAGCCACCCCTCACCCACTAGGATACCAACAAACCTACCCACCCTTAACAGTACATAGTACATAAAGCCATTTACCGTACATAGCACATTACAGTCAAATCCCTTCTCGTCCCCATGGATGACCCCCCTCAG

clone 1 tooth 1    L16119............................................................................H16196
clone 2 tooth 1    ............................................................................
clone 3 tooth 1    ............................................................................
clone 4 tooth 1    ............................................................................
clone 5 tooth 2    ............................................................................
clone 6 tooth 2    ............................................................................
clone 7 tooth 2    ............................................................................
clone 8 tooth 2    ............................................................................
clone 9 tooth 2    ............................................................................
clone 1 tooth 1    L16181..........................................C........................H16249
clone 2 tooth 1    ..........................................C........................
clone 3 tooth 1    ..........................................C........................
clone 4 tooth 1    ..........................................C........................
clone 5 tooth 1    ..........................................C........................
clone 6 tooth 2    ..........................................C........................
clone 7 tooth 2    ..........................................C........................
clone 8 tooth 2    ..............A...........................C........................
clone 1 tooth 1    L16209..............C......................................................................................C.....................T......................H16356
clone 2 tooth 1    ..............C......................................................................................C............................................
clone 3 tooth 1    ..............C........G.............................................................................C.....................T......................
clone 4 tooth 1    ..............C......................................................................................C.....................T......................
clone 5 tooth 1    ..............C......................................................................................C.....................T......................
clone 6 tooth 2    ........T.....C.............................................T...................................T....C..........................T..........T...T..
clone 7 tooth 2    ..............C......................................................................................C............................................
clone 8 tooth 2    .....................................................................................................C............................................
clone 9 tooth 2    ..............C......................................................................................C............................................
clone 10 tooth 2   ........T.....C.............................................T.....................T.............T....C..........................T..........T...T..
```

Sample C8 (ME)

```
11111111111111111111111111111111111111111111111111111111111111111111111111111111111111111111111111111111111111111111111111111111111111111111111111111111111111111111111111111111111111111111111111111111111111111111111111111111111111111111111111111111111111111111111111111111111111111111111111111111111111111111111111111111111111111111
66666666666666666666666666666666666666666666666666666666666666666666666666666666666666666666666666666666666666666666666666666666666666666666666666666666666666666666666666666666666666666666666666666666666666666666666666666666666666666666666666666666666666666666666666666666666666666666666666666666666666666666666666666666666666666666
0000000000000000000000000000000000000000000000000111111111111111111111111111111111111111111111111111111111111111111111111111111111111111111111111111111111222222222222222222222222222222222222222222222222222222222222222222222222222222222222222222222222222222222222222222233333333333333333333333333333333333333333333333333333333333333333333333333333333333
5555555556666666666777777777788888888889999999999000000000011111111112222222222333333333344444444445555555555666666666677777777778888888888999999999900000000001111111111222222222233333333334444444444555555555566666666667777777777888888888899999999990000000000111111111122222222223333333333444444444455555555556666666666777777777788888
1234567890123456789012345678901234567890123456789012345678901234567890123456789012345678901234567890123456789012345678901234567890123456789012345678901234567890123456789012345678901234567890123456789012345678901234567890123456789012345678901234567890123456789012345678901234567890123456789012345678901234567890123456789012345678901234

CRS   ACCACCCAAGTATTGACTCACCCATCAACAACCGCTATGTATTTCGTACATTACTGCCAGCCACCATGAATATTGTACGGTACCATAAATACTTGACCACCTGTAGTACATAAAAACCCAATCCACATCAAAACCCCCTCCCCATGCTTACAAGCAAGTACAGCAATCAACCCTCAACTATCACACATCAACTGCAACTCCAAAGCCACCCCTCACCCACTAGGATACCAACAAACCTACCCACCCTTAACAGTACATAGTACATAAAGCCATTTACCGTACATAGCACATTACAGTCAAATCCCTTCTCGTCCCCATGGATGACCCCCCTCAG

clone 1 tooth 1    L16119............................................................................H16196
clone 2 tooth 1    ............................................................................
clone 3 tooth 1    ............................................................................
clone 4 tooth 1    .............T..................................TT...............T..T.......
clone 5 tooth 1    ....................................A.......................................
clone 6 tooth 2    .........A..................................................................
clone 7 tooth 2    ............................................................................
clone 8 tooth 2    .................................A..........................................
clone 9 tooth 2    .................................A..........................................
clone 10 tooth 2   ............................................................................
clone 1 tooth 1    L16181................................A.......T.C...................A....H16249
clone 2 tooth 1    ......T........T.....................T..T.C........................
clone 3 tooth 1    ......T.......................T.T.......T.........T................
clone 4 tooth 1    ..........T..............................T.........................
clone 5 tooth 1    .......................Y..Y.............T.C........................
clone 6 tooth 2    ........................................T.C........................
clone 7 tooth 2    ...................................................................
clone 8 tooth 2    .............................T..........T.C.................T......
clone 9 tooth 2    ........................................T.C........................
clone 10 tooth 2   ........................................T.C........................
clone 1 tooth 1    L16209...........TT.CT..T.................................T.......T.................T........................C..........................................H16356
clone 2 tooth 1    ...........TT.CT..T.................................T.......T.................T........................C..........................................
clone 3 tooth 1    ...................................................T..............................................................................................
clone 4 tooth 1    ....................................................T.......T.................T........................C..........................................
clone 5 tooth 1    ............T.C.............................................T..........................................C..........................................
clone 6 tooth 1    ...........TT.CT..T.................................T.......T.................T........................C..........................................
clone 7 tooth 1    ............T.C.............................................T..........................................C..........................................
clone 8 tooth 2    ............T.C.......................T........Y............T..........................................C..........................................
clone 9 tooth 2    ............T.C...............................T....T........T..........................................C...................T......................
clone 10 tooth 2   ............T.C.......................T.....................T..........................................C..........................................
clone 11 tooth 2   ............T.C.......................T.....................T..........................................C..........................................
clone 12 tooth 2   ....T.......T.C.................T.............T.............T..........................................C.............T...........................
clone 13 tooth 2   ............T.C...............................T....T........T..........................................C...................T......................
clone 14 tooth 2   ............T.C...............................T....T........T..........................................C...................T......................
```

Sample C9 (ME)

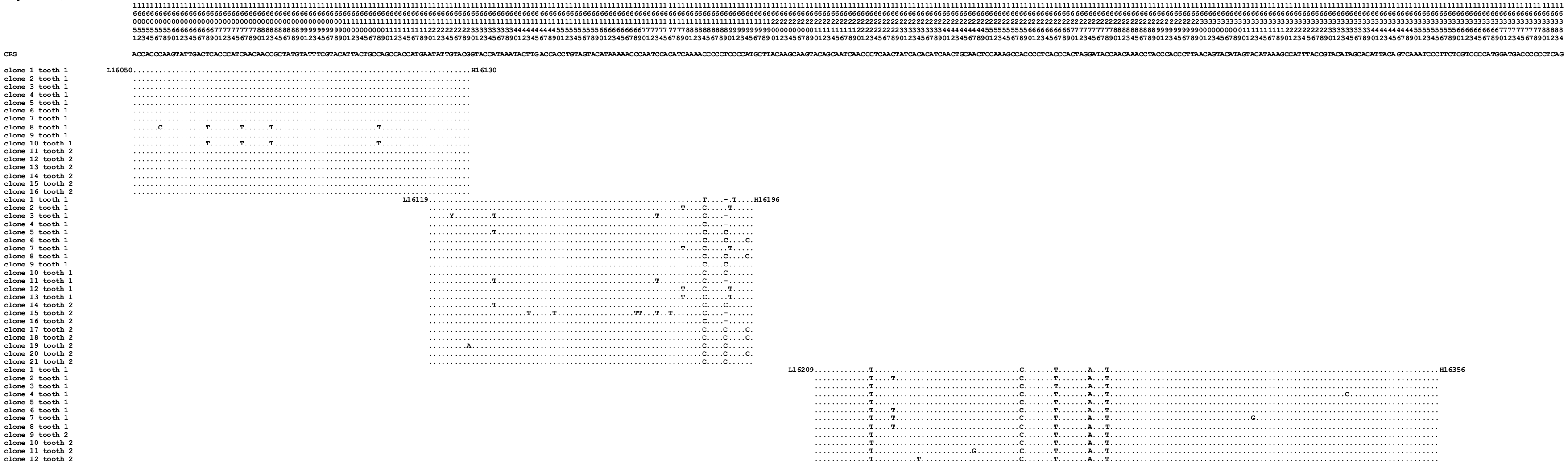

Sample C10 (ME)

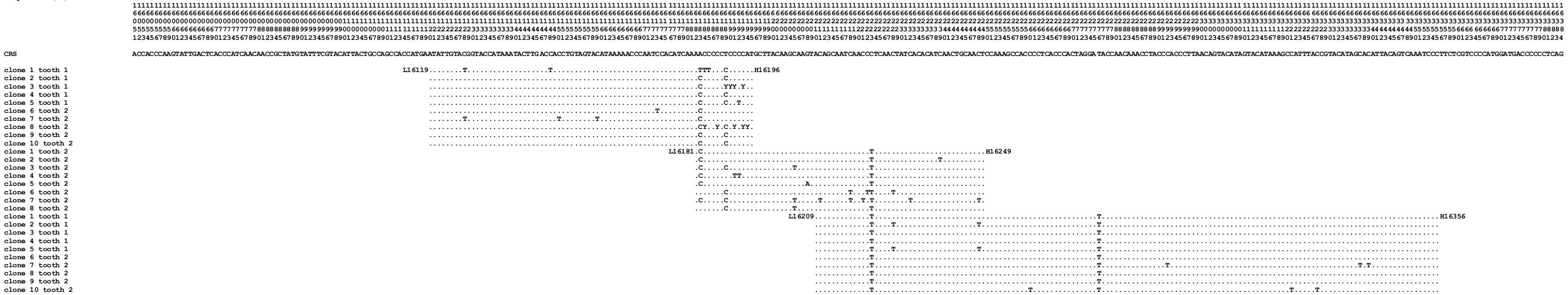

Sample C11 (ME)

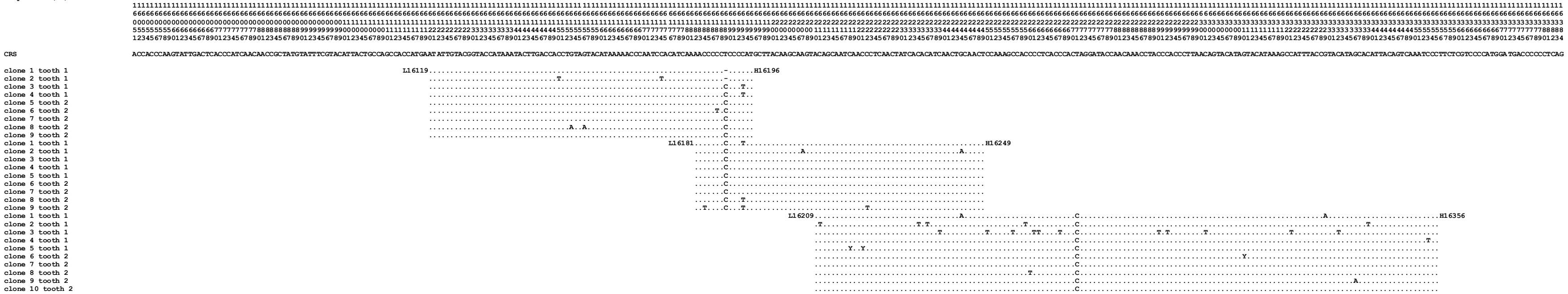

Sample C12 (ME)

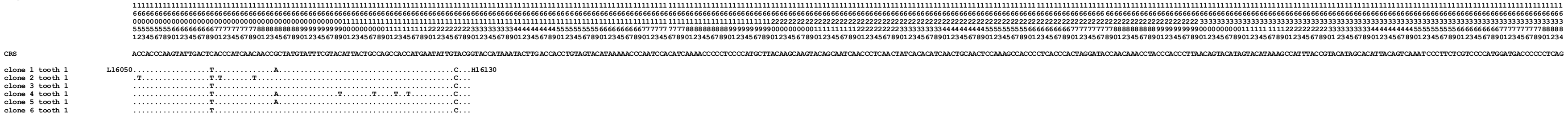

```
clone 7 tooth 1               ..................T........................................................C...
clone 8 tooth 1               ..................T........................................................C...
clone 9 tooth 1               ..................T....................................A...........A......AC..A
clone 10 tooth 1              ..................T........................................................C...
clone 11 tooth 1              ..................T........................................................C...
clone 1 tooth 1                                                                L16119......C..AC..............A..................................................H16196
clone 2 tooth 1                                                                      ......C.......T..........A........................................T.........
clone 3 tooth 1                                                                      ......C..................A..................................................
clone 4 tooth 1                                                                      ......C..................A.......A..A.......................................
clone 5 tooth 1                                                                      ......C..................A..................................................
clone 6 tooth 1                                                                      ......C..................A..................................................
clone 7 tooth 1                                                                      ......C..................A..................................................
clone 8 tooth 1                                                                      ......C..................A..................................................
clone 9 tooth 1                                                                      ......C..................A..................................................
clone 10 tooth 1                                                                     ......C..................A..................................................
clone 11 tooth 1                                                                     ......C..................A..................................................
clone 12 tooth 1                                                                     ......C..................A..................................................
clone 13 tooth 1                                                                     ......C..................A..................................................
clone 14 tooth 2                                                                     ......C..................A..................................................
clone 15 tooth 2                                                                     ......C..................A..................................................
clone 16 tooth 2                                                                     ......C................T.A.............T...................T................
clone 17 tooth 2                                                                     ......C.Y..............Y.A............Y.....................................
clone 18 tooth 2                                                                     ......C..................A..................................................
clone 19 tooth 2                                                                     ......C..................A..................................................
clone 20 tooth 2                                                                     ......C..................A.....................T......T.T..........T...AT.GA
clone 21 tooth 2                                                                     ......C..................A..................................................
clone 1 tooth 2                                                                                                                             L16181.........T.T....................T...T...T..........................H16249
clone 2 tooth 2                                                                                                                                   ........................................T..........................
clone 3 tooth 2                                                                                                                                   ........................................T..........................
clone 4 tooth 2                                                                                                                                   ........T...............................T..........................
clone 1 tooth 1                                                                                                                                                             L16209............T......................................T............................T..............................................C..................H16356
clone 2 tooth 1                                                                                                                                                                   ............T......................................T............................T.................................................................
clone 3 tooth 1                                                                                                                                                                   ............T......................................T............................T.................................................................
clone 4 tooth 1                                                                                                                                                                   ............T......................................T............................T.................................................................
clone 5 tooth 1                                                                                                                                                                   ............T......................................T............................T.................................................................
clone 6 tooth 1                                                                                                                                                                   ............T......................................T............................T.................................................................
clone 7 tooth 1                                                                                                                                                                   ............T......................................T.............................T...............................................................T
clone 8 tooth 1                                                                                                                                                                   ............T......................................T.T............................................................................................
clone 9 tooth 1                                                                                                                                                                   ............T....................................T.T.................T............................................................................
clone 10 tooth 1                                                                                                                                                                  ............T......................................T.............................T................................................................
clone 11 tooth 2                                                                                                                                                                  ............T......................................T..............................................................................................
clone 12 tooth 2                                                                                                                                                                  ............T......................................T..............................................................................................
clone 13 tooth 2                                                                                                                                                                  ............T.....................A..........A.....T..............................................................................................
clone 14 tooth 2                                                                                                                                                                  ............T......................................T..............................................................................................
clone 15 tooth 2                                                                                                                                                                  ............T......................................T.........................................................................N....................
clone 16 tooth 2                                                                                                                                                                  ............T...........T..........................T.......................T.............................................T........................
clone 17 tooth 2                                                                                                                                                                  ............T.....................A..........A.....T..............................................................................................
clone 18 tooth 2                                                                                                                                                                  ............T......................................T...............................................A..............................................
```

Sample C13 (ME)

```
                              1111111111111111111111111111111111111111111111111111111111111111111111111111111111111111111111111111111111111111111111111111111111111111111111111111111111111111111111111111111111111111111111111111111111111111111111111111111111111111111111111111111111111111111111111111111111111111111111111111111111111111111111111111111111111111111111
                              6666666666666666666666666666666666666666666666666666666666666666666666666666666666666666666666666666666666666666666666666666666666666666666666666666666666666666666666666666666666666666666666666666666666666666666666666666666666666666666666666666666666666666666666666666666666666666666666666666666666666666666666666666666666666666666666
                              0000000000000000000000000000000000000000000000000111111111111111111111111111111111111111111111111111111111111111111111111111111111111111111111111111122222222222222222222222222222222222222222222222222222222222222222222222222222222222222222222222222222222333333333333333333333333333333333333333333333333333333333333333333333333333333333333
                              5555555556666666666777777777788888888889999999999000000000011111111112222222222333333333344444444445555555555666666666677777777778888888888999999999900000000001111111111222222222233333333334444444444555555555566666666667777777777888888888899999999990000000000111111111122222222223333333333444444444455555555556666666666777777777788888
                              1234567890123456789012345678901234567890123456789012345678901234567890123456789012345678901234567890123456789012345678901234567890123456789012345678901234567890123456789012345678901234567890123456789012345678901234567890123456789012345678901234567890123456789012345678901234567890123456789012345678901234567890123456789012345678901234

CRS                           ACCACCCAAGTATTGACTCACCCATCAACAACCGCTATGTATTTCGTACATTACTGCCAGCCACCATGAATATTGTACGGTACCATAAATACTTGACCACCTGTAGTACATAAAAACCCAATCCACATCAAAACCCCCTCCCCATGCTTACAAGCAAGTACAGCAATCAACCCTCAACTATCACACATCAACTGCAACTCCAAAGCCACCCCTCACCCACTAGGATACCAACAAACCTACCCACCCTTAACAGTACATAGTACATAAAGCCATTTACCGTACATAGCACATTACAGTCAAATCCCTTCTCGTCCCCATGGATGACCCCCCTCAG

clone 1 tooth 2         L16050................................T..........................................C...H16130
clone 2 tooth 2               .............................................................T.T...........C...
clone 3 tooth 2               ...........................................................................C...
clone 4 tooth 2               ...........................................................................C...
clone 5 tooth 2               ...........................................................................C...
clone 6 tooth 2               ...........................................................................C...
clone 7 tooth 2               ...........................................................................C...
clone 1 tooth 1                                                                L16119......C.....................................................................H16196
clone 2 tooth 1                                                                      ......C.....................................................................
clone 3 tooth 1                                                                      ......C.....................................................................
clone 4 tooth 1                                                                      ......C.....................................................................
clone 5 tooth 1                                                                      ......C.....................................................................
clone 6 tooth 1                                                                      ......C.....................................................................
clone 7 tooth 1                                                                      ......C.....................................................................
clone 8 tooth 1                                                                      ......C.....................................................................
clone 9 tooth 2                                                                      ......C.....................................................................
clone 10 tooth 2                                                                     ......C.....................................................................
clone 11 tooth 2                                                                     ......C..................................................................T..
clone 12 tooth 2                                                                     ......C....................T...........T.........T......T...................
clone 13 tooth 2                                                                     ......C.....................................................................
clone 14 tooth 2                                                                     ......C.....................................................................
clone 15 tooth 2                                                                     ......C...................................................................Y.
clone 1 tooth 1                                                                                                                             L16181...................................................................H16249
clone 2 tooth 1                                                                                                                                   .........................................................T.........
clone 3 tooth 1                                                                                                                                   .........................................T...............T.........
clone 4 tooth 1                                                                                                                                   ...................................................................
clone 5 tooth 1                                                                                                                                   ...................................C...............................
clone 6 tooth 1                                                                                                                                   ...................................................................
clone 7 tooth 1                                                                                                                                   ...................................................................
clone 8 tooth 2                                                                                                                                   ...................................................................
clone 9 tooth 2                                                                                                                                   ...................................................................
clone 10 tooth 2                                                                                                                                  ...................................................................
clone 11 tooth 2                                                                                                                                  ...................................................................
clone 12 tooth 2                                                                                                                                  ...................................................................
clone 1 tooth 1                                                                                                                                                             L16209..................................A......................................................................................A........................H16356
clone 2 tooth 1                                                                                                                                                                   ..................................................................................................................................................
clone 3 tooth 1                                                                                                                                                                   ..................................................................................................................................................
clone 4 tooth 1                                                                                                                                                                   ..................................................................................................................................................
clone 5 tooth 1                                                                                                                                                                   ...............................................................................................A..................................................
clone 6 tooth 1                                                                                                                                                                   .T.....................................................................................T..........................................................
clone 7 tooth 2                                                                                                                                                                   ..................................................................................................................................................
clone 8 tooth 2                                                                                                                                                                   ..................................................................................................................................................
clone 9 tooth 2                                                                                                                                                                   ..................................................................................................................................................
clone 10 tooth 2                                                                                                                                                                  ..................................................................................................................................................
clone 11 tooth 2                                                                                                                                                                  ..................................................................................................................................................
```

Sample C14 (ME)

```
                              1111111111111111111111111111111111111111111111111111111111111111111111111111111111111111111111111111111111111111111111111111111111111111111111111111111111111111111111111111111111111111111111111111111111111111111111111111111111111111111111111111111111111111111111111111111111111111111111111111111111111111111111111111111111111111111111
                              6666666666666666666666666666666666666666666666666666666666666666666666666666666666666666666666666666666666666666666666666666666666666666666666666666666666666666666666666666666666666666666666666666666666666666666666666666666666666666666666666666666666666666666666666666666666666666666666666666666666666666666666666666666666666666666666
                              0000000000000000000000000000000000000000000000000111111111111111111111111111111111111111111111111111111111111111111111111111111111111111111111111111122222222222222222222222222222222222222222222222222222222222222222222222222222222222222222222222222222222333333333333333333333333333333333333333333333333333333333333333333333333333333333333
                              5555555556666666666777777777788888888889999999999000000000011111111112222222222333333333344444444445555555555666666666677777777778888888888999999999900000000001111111111222222222233333333334444444444555555555566666666667777777777888888888899999999990000000000111111111122222222223333333333444444444455555555556666666666777777777788888
                              1234567890123456789012345678901234567890123456789012345678901234567890123456789012345678901234567890123456789012345678901234567890123456789012345678901234567890123456789012345678901234567890123456789012345678901234567890123456789012345678901234567890123456789012345678901234567890123456789012345678901234567890123456789012345678901234

CRS                           ACCACCCAAGTATTGACTCACCCATCAACAACCGCTATGTATTTCGTACATTACTGCCAGCCACCATGAATATTGTACGGTACCATAAATACTTGACCACCTGTAGTACATAAAAACCCAATCCACATCAAAACCCCCTCCCCATGCTTACAAGCAAGTACAGCAATCAACCCTCAACTATCACACATCAACTGCAACTCCAAAGCCACCCCTCACCCACTAGGATACCAACAAACCTACCCACCCTTAACAGTACATAGTACATAAAGCCATTTACCGTACATAGCACATTACAGTCAAATCCCTTCTCGTCCCCATGGATGACCCCCCTCAG

clone 1 tooth 1                                                                L16119.........A...............A...............................G..................H16196
clone 2 tooth 1                                                                      .........A...............A..................................................
clone 3 tooth 1                                                                      .........A...............A.T...........................................T....
clone 4 tooth 1                                                                      .........A...............A..................................................
clone 5 tooth 1                                                                      .........A...............A.......A..........................................
clone 6 tooth 1                                                                      .........A...............A.......................................TT.........
clone 7 tooth 2                                                                      .........Y...............A..................................................
clone 8 tooth 2                                                                      .........A...............A..................................................
clone 9 tooth 2                                                                      .........A...............A..................................................
clone 10 tooth 2                                                                     .........A...............A..................................................
clone 11 tooth 2                                                                     .........A...............A..................................................
clone 1 tooth 1                                                                                                                             L16181.......................................................T...........H16249
clone 2 tooth 1                                                                                                                                   ...................................................................
clone 3 tooth 1                                                                                                                                   ..............................T..........T.........................
clone 4 tooth 1                                                                                                                                   ...................................................................
clone 5 tooth 1                                                                                                                                   ...................................................................
clone 6 tooth 2                                                                                                                                   ..........................A........................................
clone 7 tooth 2                                                                                                                                   ...............T.....................................T.T...........
clone 8 tooth 2                                                                                                                                   .........T.............T................T..........................
clone 9 tooth 2                                                                                                                                   ...................................................................
clone 10 tooth 2                                                                                                                                  ...................................................................
clone 1 tooth 1                                                                                                                                                             L16209..................................A............................A........................C....................A................A...................H16356
clone 2 tooth 1                                                                                                                                                                   ..................................A.....................................................C............C............................................
clone 3 tooth 1                                                                                                                                                                   ........................................T............................................TT.........T......T......TT..................................
clone 4 tooth 1                                                                                                                                                                   ........................................................................................C.........................................................
clone 5 tooth 1                                                                                                                                                                   ........................................................................................C.........................................................
clone 6 tooth 2                                                                                                                                                                   .......................................Y.....Y..........................................C.........................................................
clone 7 tooth 2                                                                                                                                                                   ............................................G...........................................C.........................................................
clone 8 tooth 2                                                                                                                                                                   ........................................................................................C.........................................................
clone 9 tooth 2                                                                                                                                                                   ........................................................................................C.....................T...................................
clone 10 tooth 2                                                                                                                                                                  ........................................................................................C...........Y.............................................
```

Sample C15 (ME)

```
                    1111111111111111111111111111111111111111111111111111111111111111111111111111111111111111111111111111111111111111111111111111111111111111111111111111111111111111111111111111111111111111111111111111111111111111111111111111111111111111111111111111111111111111111111111111111111111111111111111111111111111111111111111111111111111111
                    6666666666666666666666666666666666666666666666666666666666666666666666666666666666666666666666666666666666666666666666666666666666666666666666666666666666666666666666666666666666666666666666666666666666666666666666666666666666666666666666666666666666666666666666666666666666666666666666666666666666666666666666666666666666666666
                    0000000000000000000000000000000000000000000000000111111111111111111111111111111111111111111111111111111111111111111111111111111111111111111111111111111111222222222222222222222222222222222222222222222222222222222222222222222222222222222222222222222222222222222222222222223333333333333333333333333333333333333333333333333333333333333333333333333333333333333
                    5555555556666666666777777777788888888889999999999000000000011111111112222222222333333333344444444445555555555666666666677777777778888888888999999999900000000001111111111222222222233333333334444444444555555555566666666667777777777888888888899999999990000000000111111111122222222223333333333444444444455555555556666666666777777777788888
                    1234567890123456789012345678901234567890123456789012345678901234567890123456789012345678901234567890123456789012345678901234567890123456789012345678901234567890123456789012345678901234567890123456789012345678901234567890123456789012345678901234567890123456789012345678901234567890123456789012345678901234567890123456789012345678901234

CRS                 ACCACCCAAGTATTGACTCACCCATCAACAACCGCTATGTATTTCGTACATTACTGCCAGCCACCATGAATATTGTACGGTACCATAAATACTTGACCACCTGTAGTACATAAAAACCCAATCCACATCAAAACCCCCTCCCCATGCTTACAAGCAAGTACAGCAATCAACCCTCAACTATCACACATCAACTGCAACTCCAAAGCCACCCCTCACCCACTAGGATACCAACAAACCTACCCACCCTTAACAGTACATAGTACATAAAGCCATTTACCGTACATAGCACATTACAGTCAAATCCCTTCTCGTCCCCATGGATGACCCCCCTCAG

clone 1 tooth 1                                                                L16119..............T..........A............................T.G..T.....T.T........H16196
clone 2 tooth 1                                                                      .........................A..............................G...................
clone 3 tooth 1                                                                      .........................A..............................G...................
clone 4 tooth 1                                                                      .........................A..............................G...........T.......
clone 5 tooth 1                                                                      .........................A..............................G...................
clone 6 tooth 2                                                                      ....................C....A..............................G...................
clone 7 tooth 2                                                                      .........................A..............................G...................
clone 8 tooth 2                                                                      .........................A..............................G...................
clone 9 tooth 2                                                                      .........AA..............A..............................G...................
clone 10 tooth 2                                                                     .....A....A..............A..............................G...................
clone 11 tooth 2                                                                     ..........A..............A..........A...................G...................
clone 1 tooth 1                                                                                                                              L16181...........................C.............T.........................H16249
clone 2 tooth 1                                                                                                                                    ......................A....C.............T.........................
clone 3 tooth 1                                                                                                                                    ...........................C.............T.........................
clone 4 tooth 1                                                                                                                                    ......................A....C.............T.........................
clone 5 tooth 1                                                                                                                                    ...........................C............AT.........................
clone 6 tooth 1                                                                                                                                    ...........................C.............T.........................
clone 7 tooth 1                                                                                                                                    ...........................C............AT.........................
clone 8 tooth 2                                                                                                                                    ...........................C.............T.........................
clone 9 tooth 2                                                                                                                                    ...........................C.........G...T.........................
clone 10 tooth 2                                                                                                                                   ...........................C.......................................
clone 11 tooth 2                                                                                                                                   ...........................C.........G...T.........................
clone 12 tooth 2                                                                                                                                   ...........................C.......................................
clone 1 tooth 1                                                                                                                                                              L16209.......C................................................................................C.........................................................H16356
clone 2 tooth 1                                                                                                                                                                    .......C................................................................................C.........................................................
clone 3 tooth 1                                                                                                                                                                    .............T....................................................................................................................................
clone 4 tooth 1                                                                                                                                                                    .......C................................................................................C.........................................................
clone 5 tooth 1                                                                                                                                                                    .......C................................................................................C.........................................................
clone 6 tooth 1                                                                                                                                                                    .............T....................................................................................................................................
clone 7 tooth 1                                                                                                                                                                    .............T......................................................................T...........T.................................................
clone 8 tooth 1                                                                                                                                                                    .......C................................................................................C.........................................................
clone 9 tooth 2                                                                                                                                                                    .............T....................................................................................................................................
clone 10 tooth 2                                                                                                                                                                   .............T....................................................................................................................................
clone 11 tooth 2                                                                                                                                                                   .............T....................................................................................................................................
clone 12 tooth 2                                                                                                                                                                   .............T............................................................................................................T......T.......C........
clone 13 tooth 2                                                                                                                                                                   .............T..................................................A.................................................................................
```

Sample OL1 (ME)

```
                    1111111111111111111111111111111111111111111111111111111111111111111111111111111111111111111111111111111111111111111111111111111111111111111111111111111111111111111111111111111111111111111111111111111111111111111111111111111111111111111111111111111111111111111111111111111111111111111111111111111111111111111111111111111111111111
                    6666666666666666666666666666666666666666666666666666666666666666666666666666666666666666666666666666666666666666666666666666666666666666666666666666666666666666666666666666666666666666666666666666666666666666666666666666666666666666666666666666666666666666666666666666666666666666666666666666666666666666666666666666666666666666
                    0000000000000000000000000000000000000000000000000111111111111111111111111111111111111111111111111111111111111111111111111111111111111111111111111111111111222222222222222222222222222222222222222222222222222222222222222222222222222222222222222222222222222222222222222222223333333333333333333333333333333333333333333333333333333333333333333333333333333333333
                    5555555556666666666777777777788888888889999999999000000000011111111112222222222333333333344444444445555555555666666666677777777778888888888999999999900000000001111111111222222222233333333334444444444555555555566666666667777777777888888888899999999990000000000111111111122222222223333333333444444444455555555556666666666777777777788888
                    1234567890123456789012345678901234567890123456789012345678901234567890123456789012345678901234567890123456789012345678901234567890123456789012345678901234567890123456789012345678901234567890123456789012345678901234567890123456789012345678901234567890123456789012345678901234567890123456789012345678901234567890123456789012345678901234

CRS                 ACCACCCAAGTATTGACTCACCCATCAACAACCGCTATGTATTTCGTACATTACTGCCAGCCACCATGAATATTGTACGGTACCATAAATACTTGACCACCTGTAGTACATAAAAACCCAATCCACATCAAAACCCCCTCCCCATGCTTACAAGCAAGTACAGCAATCAACCCTCAACTATCACACATCAACTGCAACTCCAAAGCCACCCCTCACCCACTAGGATACCAACAAACCTACCCACCCTTAACAGTACATAGTACATAAAGCCATTTACCGTACATAGCACATTACAGTCAAATCCCTTCTCGTCCCCATGGATGACCCCCCTCAG

clone 1 tooth 1                                                                L16119............................................................................H16196
clone 2 tooth 1                                                                      ............................................................................
clone 3 tooth 1                                                                      ............................................................................
clone 4 tooth 2                                                                      .........A..................................................................
clone 5 tooth 2                                                                      ..............A.............................................................
clone 6 tooth 2                                                                      ..............A.............................................................
clone 7 tooth 2                                                                      ..........A.................................................................
clone 8 tooth 2                                                                      ............................................................................
clone 9 tooth 2                                                                      .......................C.A..................................................
clone 10 tooth 2                                                                     ............................................................................
clone 1 tooth 1                                                                                                                              L16181.........T.........................................................H16249
clone 2 tooth 1                                                                                                                                    ...................................................................
clone 3 tooth 1                                                                                                                                    ................................Y.............................Y....
clone 4 tooth 1                                                                                                                                    ...................................................................
clone 5 tooth 2                                                                                                                                    ........................................T..........................
clone 6 tooth 2                                                                                                                                    ...................................................................
clone 7 tooth 2                                                                                                                                    ........................................T..........................
clone 1 tooth 1                                                                                                                                                              L16209...................................................G..........................................C...................................................H16356
clone 2 tooth 1                                                                                                                                                                    ......................................T.......................................................C...................................................
clone 3 tooth 1                                                                                                                                                                    ...................................................G..........................................C...................................................
clone 4 tooth 1                                                                                                                                                                    ....T.................................T.......................G...............................C...................................................
clone 5 tooth 1                                                                                                                                                                    ..............................................................................................C...................................................
clone 6 tooth 2                                                                                                                                                                    ..............................................................................................C...................................................
clone 7 tooth 2                                                                                                                                                                    ..............................................................................................C...................................................
clone 8 tooth 2                                                                                                                                                                    ..............................................................................................C...................................................
clone 9 tooth 2                                                                                                                                                                    ..............................................................................................C...................................................
clone 10 tooth 2                                                                                                                                                                   ..............................................................................................C...................................................
clone 11 tooth 2                                                                                                                                                                   ..............................................................................................C...................................................
```

Sample OL2 (ME)

```
                    1111111111111111111111111111111111111111111111111111111111111111111111111111111111111111111111111111111111111111111111111111111111111111111111111111111111111111111111111111111111111111111111111111111111111111111111111111111111111111111111111111111111111111111111111111111111111111111111111111111111111111111111111111111111111111
                    6666666666666666666666666666666666666666666666666666666666666666666666666666666666666666666666666666666666666666666666666666666666666666666666666666666666666666666666666666666666666666666666666666666666666666666666666666666666666666666666666666666666666666666666666666666666666666666666666666666666666666666666666666666666666666
                    0000000000000000000000000000000000000000000000000111111111111111111111111111111111111111111111111111111111111111111111111111111111111111111111111111111111222222222222222222222222222222222222222222222222222222222222222222222222222222222222222222222222222222222222222222223333333333333333333333333333333333333333333333333333333333333333333333333333333333333
                    5555555556666666666777777777788888888889999999999000000000011111111112222222222333333333344444444445555555555666666666677777777778888888888999999999900000000001111111111222222222233333333334444444444555555555566666666667777777777888888888899999999990000000000111111111122222222223333333333444444444455555555556666666666777777777788888
                    1234567890123456789012345678901234567890123456789012345678901234567890123456789012345678901234567890123456789012345678901234567890123456789012345678901234567890123456789012345678901234567890123456789012345678901234567890123456789012345678901234567890123456789012345678901234567890123456789012345678901234567890123456789012345678901234

CRS                 ACCACCCAAGTATTGACTCACCCATCAACAACCGCTATGTATTTCGTACATTACTGCCAGCCACCATGAATATTGTACGGTACCATAAATACTTGACCACCTGTAGTACATAAAAACCCAATCCACATCAAAACCCCCTCCCCATGCTTACAAGCAAGTACAGCAATCAACCCTCAACTATCACACATCAACTGCAACTCCAAAGCCACCCCTCACCCACTAGGATACCAACAAACCTACCCACCCTTAACAGTACATAGTACATAAAGCCATTTACCGTACATAGCACATTACAGTCAAATCCCTTCTCGTCCCCATGGATGACCCCCCTCAG

clone 1 tooth 1                                                                L16119............................................................................H16196
clone 2 tooth 1                                                                      ............................................................................
clone 3 tooth 1                                                                      ............................................................................
clone 4 tooth 1                                                                      ............................................................................
clone 5 tooth 1                                                                      ............................................................................
clone 6 tooth 2                                                                      ............................................................................
clone 7 tooth 2                                                                      ............................................................................
clone 8 tooth 2                                                                      ............................................................................
clone 9 tooth 2                                                                      ............................................................................
clone 10 tooth 2                                                                     ............................................................................
clone 1 tooth 2                                                                                                                              L16181.........T.........................................................H16249
clone 2 tooth 2                                                                                                                                    .........T.........................................................
clone 3 tooth 2                                                                                                                                    .........T.........................................................
clone 1 tooth 2                                                                                                                                                              L16209.....................................................................................................C............................................H16356
clone 2 tooth 2                                                                                                                                                                    .............T......G................................................................................C........T...................................
clone 3 tooth 1                                                                                                                                                                    .....................................................................................................C............................................
clone 4 tooth 1                                                                                                                                                                    .....................................................................................................C............................................
clone 5 tooth 1                                                                                                                                                                    .....................................................................................................C............................................
clone 6 tooth 1                                                                                                                                                                    .............T......G................................................................................C........T...................................
clone 7 tooth 1                                                                                                                                                                    .....................................................................................................C............................................
```

Sample OL3 (ME)

```
                    1111111111111111111111111111111111111111111111111111111111111111111111111111111111111111111111111111111111111111111111111111111111111111111111111111111111111111111111111111111111111111111111111111111111111111111111111111111111111111111111111111111111111111111111111111111111111111111111111111111111111111111111111111111111111111
                    6666666666666666666666666666666666666666666666666666666666666666666666666666666666666666666666666666666666666666666666666666666666666666666666666666666666666666666666666666666666666666666666666666666666666666666666666666666666666666666666666666666666666666666666666666666666666666666666666666666666666666666666666666666666666666
                    0000000000000000000000000000000000000000000000000111111111111111111111111111111111111111111111111111111111111111111111111111111111111111111111111111111111222222222222222222222222222222222222222222222222222222222222222222222222222222222222222222222222222222222222222222223333333333333333333333333333333333333333333333333333333333333333333333333333333333333
                    5555555556666666666777777777788888888889999999999000000000011111111112222222222333333333344444444445555555555666666666677777777778888888888999999999900000000001111111111222222222233333333334444444444555555555566666666667777777777888888888899999999990000000000111111111122222222223333333333444444444455555555556666666666777777777788888
                    1234567890123456789012345678901234567890123456789012345678901234567890123456789012345678901234567890123456789012345678901234567890123456789012345678901234567890123456789012345678901234567890123456789012345678901234567890123456789012345678901234567890123456789012345678901234567890123456789012345678901234567890123456789012345678901234

CRS                 ACCACCCAAGTATTGACTCACCCATCAACAACCGCTATGTATTTCGTACATTACTGCCAGCCACCATGAATATTGTACGGTACCATAAATACTTGACCACCTGTAGTACATAAAAACCCAATCCACATCAAAACCCCCTCCCCATGCTTACAAGCAAGTACAGCAATCAACCCTCAACTATCACACATCAACTGCAACTCCAAAGCCACCCCTCACCCACTAGGATACCAACAAACCTACCCACCCTTAACAGTACATAGTACATAAAGCCATTTACCGTACATAGCACATTACAGTCAAATCCCTTCTCGTCCCCATGGATGACCCCCCTCAG

clone 1 tooth 1                                                                L16119......C....................................G......................T..C......H16196
clone 2 tooth 1                                                                      ......C....................................G......................T..C......
clone 3 tooth 1                                                                      ......C....................................G......................T..C......
clone 4 tooth 1                                                                      ......C....................................G......................T..C......
clone 5 tooth 1                                                                      .....AC....................................G......................T..C......
clone 6 tooth 2                                                                      ......C....................................G......................T..C......
clone 7 tooth 2                                                                      ......C....................................G......................T..C......
clone 8 tooth 2                                                                      ......C....................................G......................T..C......
clone 9 tooth 2                                                                      ......C....................................G......................T..C......
clone 10 tooth 2                                                                     ......C....................................G......................T..C......
clone 1 tooth 1                                                                                                                              L16181....T..C...........................................................H16249
clone 2 tooth 1                                                                                                                                    ....T..C......................................................A....
clone 3 tooth 1                                                                                                                                    ....T..C......A....................................................
clone 4 tooth 1                                                                                                                                    ....T..C...........................................................
clone 5 tooth 1                                                                                                                                    ....T..C...........................................................
clone 6 tooth 2                                                                                                                                    ....T..C...........................................................
clone 7 tooth 2                                                                                                                                    ...................................................................
clone 1 tooth 1                                                                                                                                                              L16209....................................................................................T.............................................................H16356
clone 2 tooth 1                                                                                                                                                                    ....................................................................................T..................................A..........................
clone 3 tooth 1                                                                                                                                                                    ....................................................................................T.............................................................
clone 4 tooth 1                                                                                                                                                                    ....................................................................................T.............................................................
clone 5 tooth 1                                                                                                                                                                    ....................................................................................T..................................A..........................
clone 6 tooth 2                                                                                                                                                                    ....................................................................................T.............................................................
clone 7 tooth 2                                                                                                                                                                    ....................................................................................T.............................................................
clone 8 tooth 2                                                                                                                                                                    ....................................................................................T.............................................................
```

Sample OL4 (ME)

```
                        1111111111111111111111111111111111111111111111111111111111111111111111111111111111111111111111111111111111111111111111111111111111111111111111111111111111111111111111111111111111111111111111111111111111111111111111111111111111111111111111111111111111111111111111111111111111111111111111111111111111111111111111111111111111
                        6666666666666666666666666666666666666666666666666666666666666666666666666666666666666666666666666666666666666666666666666666666666666666666666666666666666666666666666666666666666666666666666666666666666666666666666666666666666666666666666666666666666666666666666666666666666666666666666666666666666666666666666666666666666
                        0000000000000000000000000000000000000000000000000111111111111111111111111111111111111111111111111111111111111111111111111111111111111111111111111111111122222222222222222222222222222222222222222222222222222222222222222222222222222222222222222222222222222222222223333333333333333333333333333333333333333333333333333333333333333333333333333333333333
                        5555555556666666666777777777788888888889999999999000000000011111111112222222222333333333344444444445555555555666666666677777777778888888888999999999900000000001111111111222222222233333333334444444444555555555566666666667777777777888888888899999999990000000000111111111122222222223333333333444444444455555555556666666666777777777788888
                        1234567890123456789012345678901234567890123456789012345678901234567890123456789012345678901234567890123456789012345678901234567890123456789012345678901234567890123456789012345678901234567890123456789012345678901234567890123456789012345678901234567890123456789012345678901234567890123456789012345678901234567890123456789012345678901234

CRS                     ACCACCCAAGTATTGACTCACCCATCAACAACCGCTATGTATTTCGTACATTACTGCCAGCCACCATGAATATTGTACGGTACCATAAATACTTGACCACCTGTAGTACATAAAAACCCAATCCACATCAAAACCCCCTCCCCATGCTTACAAGCAAGTACAGCAATCAACCCTCAACTATCACACATCAACTGCAACTCCAAAGCCACCCCTCACCCACTAGGATACCAACAAACCTACCCACCCTTAACAGTACATAGTACATAAAGCCATTTACCGTACATAGCACATTACAGTCAAATCCCTTCTCGTCCCCATGGATGACCCCCCTCAG

clone 1 tooth 1   L16050........G.........TG.......................................................C...H16130
clone 2 tooth 1         ..................T........................................................C...
clone 3 tooth 1         ..................T........................................................C...
clone 4 tooth 1         ..................T........................................................C...
clone 5 tooth 1         ..................T........................................................C...
clone 1 tooth 1                                                                        L16119......C..................A..........................C.......................H16196
clone 2 tooth 1                                                                              ......C..................A..........Y...............C.......................
clone 3 tooth 1                                                                              ......C..................A..........................C.......................
clone 4 tooth 1                                                                              ......C..................A..........................C.......................
clone 5 tooth 1                                                                              ......C..................A..........................C.......................
clone 6 tooth 1                                                                              ......C..A...............A..........................C.......................
clone 7 tooth 2                                                                              ......C..................A..........................C.......................
clone 8 tooth 2                                                                              ......C..................A..........................C.......................
clone 9 tooth 2                                                                              ......C..................A..........................C................Y......
clone 10 tooth 2                                                                             ......C..................AG.........................C.......................
clone 1 tooth 2                                                                                                                                      L16181.Y......................................T..........................H16249
clone 2 tooth 2                                                                                                                                            ........................................T..........................
clone 3 tooth 2                                                                                                                                            ........................................T..........................
clone 4 tooth 2                                                                                                                                            ........................................T..........................
clone 1 tooth 1                                                                                                                                                                  L16209............T....................................T.T........T.........................................................................T....G......H16356
clone 2 tooth 1                                                                                                                                                                        ............T...........T...............T.....T...TT.....T................................................................................T.......
clone 3 tooth 1                                                                                                                                                                        ............T................T.....................T...........................................................................................T..
clone 4 tooth 1                                                                                                                                                                        ............T......................................T............................................................................................T.
clone 5 tooth 1                                                                                                                                                                        ............T......................................TT.............................................................................................
clone 6 tooth 2                                                                                                                                                                        ............T......................................T..............................................................................................
clone 7 tooth 2                                                                                                                                                                        ............T......................................T..............................................................................................
```

Sample OL5 (ME)

```
                        1111111111111111111111111111111111111111111111111111111111111111111111111111111111111111111111111111111111111111111111111111111111111111111111111111111111111111111111111111111111111111111111111111111111111111111111111111111111111111111111111111111111111111111111111111111111111111111111111111111111111111111111111111111111
                        6666666666666666666666666666666666666666666666666666666666666666666666666666666666666666666666666666666666666666666666666666666666666666666666666666666666666666666666666666666666666666666666666666666666666666666666666666666666666666666666666666666666666666666666666666666666666666666666666666666666666666666666666666666666
                        0000000000000000000000000000000000000000000000000111111111111111111111111111111111111111111111111111111111111111111111111111111111111111111111111111111122222222222222222222222222222222222222222222222222222222222222222222222222222222222222222222222222222222222223333333333333333333333333333333333333333333333333333333333333333333333333333333333333
                        5555555556666666666777777777788888888889999999999000000000011111111112222222222333333333344444444445555555555666666666677777777778888888888999999999900000000001111111111222222222233333333334444444444555555555566666666667777777777888888888899999999990000000000111111111122222222223333333333444444444455555555556666666666777777777788888
                        1234567890123456789012345678901234567890123456789012345678901234567890123456789012345678901234567890123456789012345678901234567890123456789012345678901234567890123456789012345678901234567890123456789012345678901234567890123456789012345678901234567890123456789012345678901234567890123456789012345678901234567890123456789012345678901234

CRS                     ACCACCCAAGTATTGACTCACCCATCAACAACCGCTATGTATTTCGTACATTACTGCCAGCCACCATGAATATTGTACGGTACCATAAATACTTGACCACCTGTAGTACATAAAAACCCAATCCACATCAAAACCCCCTCCCCATGCTTACAAGCAAGTACAGCAATCAACCCTCAACTATCACACATCAACTGCAACTCCAAAGCCACCCCTCACCCACTAGGATACCAACAAACCTACCCACCCTTAACAGTACATAGTACATAAAGCCATTTACCGTACATAGCACATTACAGTCAAATCCCTTCTCGTCCCCATGGATGACCCCCCTCAG

clone 1 tooth 1                                                                        L16119.....................................................................Y......H16196
clone 2 tooth 1                                                                              ........T....T.............T.........................................C......
clone 3 tooth 1                                                                              ............................................................................
clone 4 tooth 1                                                                              ............................................................................
clone 5 tooth 1                                                                              ....................................A.......................................
clone 6 tooth 1                                                                              .........................Y..................................................
clone 7 tooth 2                                                                              ............................................................................
clone 8 tooth 2                                                                              ................................................T...........................
clone 1 tooth 1                                                                                                                                      L16181.........................................T.........Y...............H16249
clone 2 tooth 1                                                                                                                                            .........................................T.........................
clone 3 tooth 1                                                                                                                                            ...................T.....................T.........................
clone 4 tooth 1                                                                                                                                            .........................................T....................A....
clone 5 tooth 1                                                                                                                                            ...............................A.........T.........................
clone 6 tooth 2                                                                                                                                            ......T..................................T.........................
clone 7 tooth 2                                                                                                                                            ...............................A.........T.........................
clone 8 tooth 2                                                                                                                                            .........................................T.........................
clone 9 tooth 2                                                                                                                                            ...............................A.........T.........................
clone 10 tooth 2                                                                                                                                           .......C.................................T.........................
clone 1 tooth 1                                                                                                                                                                  L16209.............T....................................................................................................................................H16356
clone 2 tooth 1                                                                                                                                                                        .............T....................................................................................................................................
clone 3 tooth 1                                                                                                                                                                        .............TC...................................................................................................................................
clone 4 tooth 1                                                                                                                                                                        .............T....................................................................................................................................
clone 5 tooth 1                                                                                                                                                                        .............TC...................................................................................................................................
clone 6 tooth 1                                                                                                                                                                        .............T....................A..............................A................................................................................
clone 7 tooth 1                                                                                                                                                                        .............T....................A..............................A................................................................................
clone 8 tooth 1                                                                                                                                                                        .............T....................A..............................A................................................................................
clone 9 tooth 1                                                                                                                                                                        .............T....................A..............................A..........G.....................................................................
clone 10 tooth 1                                                                                                                                                                       .............T....................A..............................A................................................................................
clone 11 tooth 2                                                                                                                                                                       .............T.....................................................................A..............................................................
clone 12 tooth 2                                                                                                                                                                       .............T.......................................................................................A............................................
clone 13 tooth 2                                                                                                                                                                       .............T....................................................................................................................................
clone 14 tooth 2                                                                                                                                                                       .............T....................................................................................................................................
clone 15 tooth 2                                                                                                                                                                       .............T....................A..............................A................................................................................
```
