# Supplementary material for: Ancient DNA Reveals Matrilineal Continuity in Present-Day Poland over the Last Two Millennia
Source: PLoS One. 2014 Oct 22;9(10):e110839. doi: 10.1371/journal.pone.0110839 (PMC4206425; doi:10.1371/journal.pone.0110839)
Supplement: Table S2 — PCR primers used in the present study. (DOCX) [file pone.0110839.s003.docx]

**Table S2.** PCR primer pairs used to amplify mtDNA sequences in the present study.

| **Name** | **Sequence (5'-3')** | **Amplicon size (bp)** | **Reference** |
| --- | --- | --- | --- |
| L16048 | CGCCAGGGTTTTCCCAGTCACGACTTCTTTCATGGGGAAGCAGA | 89 | Present study |
| H16132 | TCACACAGGAAACAGCTATGACCAGGTGGTCAAGTATTTATGGT |  |  |
| L16050 | GGGAAGCAGATTTGGGT | 120 | [1] |
| H16130 | AGGTGGTCAAGTATTTATGGTAC |  |  |
| L16119 | TTACTGCCAGCCACCATGA | 118 | [1] |
| H16196 | GATTGCTGTACTTGCTTGTAAGC |  |  |
| L16181 | CATAAAAACCCAATCCACATCAA | 109 | [1] |
| H16249 | GGTGAGGGGTGGCTTTGG |  |  |
| L16249 | AACTATCACACATCAACTGCAACT | 113 | [1] |
| H16317 | TGCTATGTACGGTAAATGGCTT |  |  |
| L16209 | CCCCATGCTTACAAGCAAGT | 145 | [2] |
| H16356 | GTCATCCATGGGGACGAGAA |  |  |
| L16307 | CGCCAGGGTTTTCCCAGTCACGACCCCACCCTTAACAGTACA | 96 | Present study |
| H16403 | TCACACAGGAAACAGCTATGACATTGATTTCACGGAGGATGG |  |  |
| H_7028_F | TACTACACGACACGTACTACG | 75 | Present study |
| H_7028_R | TAGGAGCTGTATTTGCCATCATAG |  |  |
| H5a1_15833_F | GCATCCGTACTATACTTCACAACA | 63 | Present study |
| H5a1_15833_R | GTTTTCAATTAGGGAGATAGTTGGT |  |  |
| X1_146,195_F | ATGTCGCAGTATCTGTCTTTG | 111 | Present study |
| X1_146,195_R | AGTGTGTTAATTAATTAATGCTTGT |  |  |
| X2_1719_F | ACCCACTCCACCTTACTACCAG | 67 | Present study |
| X2_1719_R | TACCCAAATAAAGTATAGGCGATAG |  |  |
| T2b_5147_F | CCTACTACTCAACTTAAACTCC | 60 | Present study |
| T2b_5147_R | TTGTTTCAGGTGCGAGATAG |  |  |
| HVO_72_F | ATGCATTTGGTATTTTCGTCTG | 53 | Present study |
| HVO_72_R | CGTCTCGCAATGCTATCG |  |  |
| R1_11719_F | GCGCAGTCATTCTCATAATC | 58 | Present study |
| R1_11719_R | GCTAGGCAGAATAGTAATGAG |  |  |
| HV_14766_F | CACCAATGACCCCAATACG | 68 | Present study |
| HV_14766_R | GAGGTCGATGAATGAGTGGTTAA |  |  |
| V_4580_F | TACCTGAGTAGGCCTAGAA | 54 | Present study |
| V_4580_R | TTGGTTAGAACTGGAATAAAAG |  |  |
| K1_1189_F | CTCAAAGGACCTGGCGGTG | 55 | Present study |
| K1_1189_R | CGATTACAGAACAGGCTCCTC |  |  |
| K2_146_F | ATGTCGCAGTATCTGTCTTT | 65 | Present study |
| K2_146_R | AATATTGAACGTAGGTGCGAT |  |  |
| ROA_F | AAAGCCATTTACCGTACATAG | 72 | Present study |
| ROA_R | TATCTGAGGGGGGTCATC |  |  |
| J_10398_F | TGGCCTATGAGTGACTACAA | 65 | Present study |
| J_10398_R | CGTTTTGTTTAAACTATATACC |  |  |
| J2a_7476_F | AAGGAAGGAATCGAACCCC | 55 | Present study |
| J2a_7476_R | ATGGAGGCCATGGGGTTG |  |  |
| U_12308_F | ACAGCTATCCATTGGTCTTA | 60 | Present study |
| U_12308_R | TATTACTTTTATTTGGAGTTGC |  |  |
| U5a1_15218_F | TACTATCCGCCATCCCATACATT | 60 | Present study |
| U5a1_15218_R | AGTAGCCTCCTCAGATTCATTG |  |  |
| U5a1a1a_3816 _F | CTCCACCCTTATCACAACACAAG | 55 | Present study |
| U5a1a1a_3816 _R | GGGTCATGATGGCAGGAGT |  |  |
| U5b_7768 | CTAATACTAACATCTCAGACGCTC | 59 | Present study |
| U5b_7768 | GGCGGGCAGGATAGTTCAGA |  |  |
| U3_14139_F | TTCCCACTCATCCTAACCCT | 55 | Present study |
| U3_14139_R | GATTGCTCGGGGGAATAGG |  |  |
| N1_10238_F | CCCTTTCTCCATAAAATTCTTC | 68 | Present study |
| N1_10238_R | GGAGGGCAATTTCTAGATCAAA |  |  |
| W_8252F | AATTAATTCCCCTAAAAATCTTTG | 58 | Present study |
| W_8252R | AGGGGGTGCTATAGGGTA |  |  |

**References**

1. Malmström H, Svensson EM, Gilbert MTP, Willerslev E, Gotherstrom A, et al. (2007) More on Contamination: The Use of Asymmetric Molecular Behavior to Identify Authentic Ancient Human DNA. Mol Biol Evol 24: 998-1004.
2. Handt O, Krings M, Ward R, Pääbo S (1996) The retrieval of ancient human DNA sequences. Am J Hum Genet 59: 368-376.
