# Supplementary material for: Ancient DNA Reveals Matrilineal Continuity in Present-Day Poland over the Last Two Millennia
Source: PLoS One. 2014 Oct 22;9(10):e110839. doi: 10.1371/journal.pone.0110839 (PMC4206425; doi:10.1371/journal.pone.0110839)
Supplement: Table S3 — Modern and ancient populations used in the comparative analysis. (DOCX) [file pone.0110839.s004.docx]

**Table S3.** Modern and ancient populations used in the comparative analysis.

| **Age/culture** | **Population** | **Symbol** | **N** | **Reference** |
| --- | --- | --- | --- | --- |
| Iron Age | Danish | DIA | 24 | [1] |
| Middle Age | Danish | DM | 29 | [1] |
| Neolithic (LBK*) | Germans | NEO | 42 | [2] |
| Modern populations | Belarusians | BY | 267 | [3] |
|  | Bosnians | BH | 144 | [4] |
|  | Bulgarians | BG | 855 | [5] |
|  | Croatians | HR | 488 | [6] |
|  | Czechs | CZ | 179 | [7] |
|  | Estonians | EE | 113 | [8] |
|  | Finns | FI | 200 | [9] |
|  | Germans | DE | 287 | [10] |
|  | Latvians | LV | 112 | [8] |
|  | Lithuanians | LT | 149 | [8] |
|  | Macedonians | MK | 200 | [11] |
|  | Poles | PL | 413 | [12] |
|  | Russians (European part) | RU | 201 | [12], [13] |
|  | Serbs | SI | 100 | [14] |
|  | Slovaks | SK | 207 | [15] |
|  | Slovenians | SLO | 104 | [4] |
|  | Swedes | SE | 286 | [16] |
|  | Ukrainians | UA | 607 | [17] |

*Linear Pottery culture

**References**

1. Melchior L, Lynnerup N, Siegismund HR, Kivisild T, Dissing J (2010) Genetic diversity among ancient Nordic populations. PLoS ONE 5: e11898.
2. Haak W, Balanovsky O, Sanchez JJ, Koshel S, Zaporozhchenko V, et al. (2010) Ancient DNA from European early Neolithic farmers reveals their Eastern Affinities. PLoS Biology 8: e1000535.
3. Kushniarevich A, Sivitskaya L, Danilenko N, Novogrodskii T, et al. (2013) Uniparental Genetic Heritage of Belarusians: Encounter of Rare Middle Matrilineages with a Central European Mitochondrial DNA Pool. PLoS ONE 8: e66499.
4. Malyarchuk BA, Grzybowski T, Derenko MV, Czarny J, Drobnič K, et al. (2003) Mitochondrial DNA variability in Bosnians and Slovenians. Ann of Hum Genet 67: 412-425.
5. Karachanak S, Carossa V, Nesheva D, Olivieri A, Pala M, et al. (2011) Bulgarians vs the other European populations: a mitochondrial DNA perspective. Int J Legal Med DOI: 10.1007/s00414-011-0589y.
6. Šarac J, Šarić T, Auguštin DB, Jeran N, Kovačević L, et al. (2014) Maternal Genetic Heritage of Southeastern Europe Reveals a New Croatian Isolate and a Novel, Local Sub-Branching in the X2 Haplogroup. Ann Hum Genet 78(3): 178-194.
7. Malyarchuk BA, Vanecek T, Perkova MA, Derenko MV, Sip M (2006) Mitochondrial DNA variability in the Czech population, with application to the ethnic history of Slavs. Hum Biol 78: 681-696.
8. Lappalainen T, Laitinen V, Salmela E, Anderson P, Huoponen K, et al. (2008) Migration waves to the Baltic Sea region. Ann Hum Genet 72: 337-348.
9. Hedman M, Brandstätter A, Pimenoff V, Sistonen P, Palo JU, et al. (2007) Finnish mitochondrial DNA HVS-I and HVS-II population data. Forensic Sci Int 172: 171-178
10. Poetsch M, Wittig H, Krause D, Lignitz E (2003) Mitochondrial diversity of a northeast German population sample. Forensic Sci Int 137: 125-132.
11. Zimmermann B, Brandstätter A, Duftner N, Niederwieser D, Spiroski M, et al. (2007) Mitochondrial DNA control region population data from Macedonia. Forensic Sci Int: Genetic 1: e4-9.
12. Grzybowski T, Malyarchuk BA, Derenko MV, Perkova MA, Bednarek J, et al. (2007) Complex interactions of the Eastern and Western Slavic populations with other European groups as revealed by mitochondrial DNA analysis. Forensic Sci Int: Genetic, 1: 141-147.
13. Malyarchuck, B A, Grzybowski, T, Derenko, MV, Czarny, J, Wozniak, et al. (2003) Mitochondrial DNA variability in Poles and Russians. Ann Hum Genet 66: 261-83.
14. Zgonjanin D, Veselinović I, Kubat M, Furac I, Antov M, et al. (2010) Sequence polymorphism of the mitochondrial DNA control region in the population of Vojvodina Povince, Serbia. Leg Med (Tokyo) 12: 104-107.
15. Malyarchuk BA, Perkova MA, Derenko MV, Vanecek T, Lazur J, et al. (2008) Mitochondrial DNA variability in Slovaks, with application to the Roma origin. Ann Hum Genet 72: 228-40.
16. Tillmar A.O, Coble MD, Wallerström T, Holmlund G (2010) Homogenity in mitochondrial DNA control region in Swedish subpopulations. Int J Legal Med, 124: 91-98.
17. Pshenichnov A, Balanovsky O, Utevska O, Metspalu E, Zaporozhchenko V, et al. (2013) Genetic Affinities of Ukrainians From the Maternal Perspective. Am J Phys Anthropol, 152: 543-550.
