# Supplementary material for: Ancient DNA Reveals Matrilineal Continuity in Present-Day Poland over the Last Two Millennia
Source: PLoS One. 2014 Oct 22;9(10):e110839. doi: 10.1371/journal.pone.0110839 (PMC4206425; doi:10.1371/journal.pone.0110839)
Supplement: Table S5 — FST distances by Slatkin's based on haplogroup frequencies (P<0.01). (DOCX) [file pone.0110839.s006.docx]

|  | **Genetic distances (Fst values by Slatkin) on distribution of haplogroup frequencies; p<0.01** | | | | | | | | |  |  |  |  |  |  |  |  |  |  |  |  |  |  |  |
| --- | --- | --- | --- | --- | --- | --- | --- | --- | --- | --- | --- | --- | --- | --- | --- | --- | --- | --- | --- | --- | --- | --- | --- | --- |
|  |  |  |  |  |  |  |  |  |  |  |  |  |  |  |  |  |  |  |  |  |  |  |  |  |
|  |  | 1 | 2 | 3 | 4 | 5 | 6 | 7 | 8 | 9 | 10 | 11 | 12 | 13 | 14 | 15 | 16 | 17 | 18 | 19 | 20 | 21 | 22 | 23 |
| 1 | RoIA-present study |  | - | - | - | + | - | - | - | - | + | + | - | - | - | - | - | - | - | + | - | - | - | + |
| 2 | ME - present study | 0.019 |  | - | - | + | - | - | - | - | - | - | - | - | - | - | - | - | - | - | - | - | - | - |
| 3 | Iron Age Denmark | 0.016 | 0.000 |  | - | + | - | - | - | - | - | - | - | - | - | - | - | - | - | - | - | - | - | - |
| 4 | Middle Ages Denmark | 0.044 | 0.000 | 0.000 |  | + | - | - | - | - | - | - | - | - | - | - | - | - | - | - | - | - | - | - |
| 5 | Neolithic (LBK, Germany) | 0.138 | 0.044 | 0.055 | 0.030 |  | + | + | + | + | + | + | + | + | + | + | + | + | + | + | + | + | + | + |
| 6 | Czech Republic | 0.016 | 0.000 | 0.000 | 0.000 | 0.067 |  | - | - | - | - | - | - | - | - | - | - | - | - | + | - | - | - | + |
| 7 | Poland | 0.014 | 0.002 | 0.000 | 0.002 | 0.068 | 0.002 |  | - | - | - | - | - | - | - | - | - | + | - | - | - | + | + | + |
| 8 | Slovakia | 0.010 | 0.000 | 0.000 | 0.002 | 0.080 | 0.000 | 0.003 |  | - | - | + | - | - | - | - | - | - | - | + | - | - | - | + |
| 9 | Russia (European part) | 0.016 | 0.000 | 0.000 | 0.000 | 0.064 | 0.000 | 0.000 | 0.000 |  | - | - | - | - | - | - | - | - | - | - | - | + | - | + |
| 10 | Ukraine | 0.029 | 0.000 | 0.000 | 0.000 | 0.047 | 0.001 | 0.000 | 0.005 | 0.000 |  | - | - | - | - | - | - | - | + | - | - | + | - | + |
| 11 | Belarus | 0.026 | 0.000 | 0.000 | 0.000 | 0.051 | 0.003 | 0.000 | 0.006 | 0.000 | 0.000 |  | - | - | - | - | + | + | + | - | - | + | + | + |
| 12 | Croatia | 0.018 | 0.003 | 0.000 | 0.000 | 0.062 | 0.000 | 0.000 | 0.003 | 0.000 | 0.000 | 0.000 |  | - | - | - | - | - | - | - | - | + | - | + |
| 13 | Bosnia and Herzegovina | 0.004 | 0.005 | 0.000 | 0.010 | 0.099 | 0.002 | 0.000 | 0.002 | 0.000 | 0.005 | 0.004 | 0.002 |  | - | - | - | + | - | + | - | - | - | + |
| 14 | Slovenia | 0.000 | 0.000 | 0.000 | 0.007 | 0.088 | 0.000 | 0.000 | 0.000 | 0.000 | 0.002 | 0.002 | 0.000 | 0.000 |  | - | - | - | - | - | - | - | - | + |
| 15 | Serbia | 0.003 | 0.000 | 0.000 | 0.000 | 0.065 | 0.000 | 0.000 | 0.000 | 0.000 | 0.000 | 0.000 | 0.000 | 0.000 | 0.000 |  | - | - | - | - | - | - | - | + |
| 16 | Macedonia | 0.006 | 0.000 | 0.000 | 0.004 | 0.075 | 0.000 | 0.002 | 0.000 | 0.001 | 0.003 | 0.005 | 0.002 | 0.002 | 0.000 | 0.000 |  | - | - | + | - | + | - | + |
| 17 | Bulgaria | 0.017 | 0.000 | 0.000 | 0.003 | 0.057 | 0.000 | 0.002 | 0.001 | 0.000 | 0.000 | 0.003 | 0.002 | 0.005 | 0.002 | 0.000 | 0.000 |  | + | + | - | + | - | + |
| 18 | Lithuania | 0.000 | 0.022 | 0.004 | 0.021 | 0.112 | 0.005 | 0.005 | 0.005 | 0.002 | 0.014 | 0.012 | 0.006 | 0.000 | 0.000 | 0.000 | 0.007 | 0.011 |  | + | - | - | + | + |
| 19 | Latvia | 0.034 | 0.018 | 0.000 | 0.001 | 0.060 | 0.012 | 0.004 | 0.012 | 0.006 | 0.003 | 0.003 | 0.006 | 0.013 | 0.010 | 0.009 | 0.010 | 0.009 | 0.020 |  | - | + | + | + |
| 20 | Estonia | 0.020 | 0.009 | 0.000 | 0.000 | 0.068 | 0.000 | 0.001 | 0.001 | 0.000 | 0.002 | 0.002 | 0.000 | 0.005 | 0.000 | 0.000 | 0.003 | 0.004 | 0.004 | 0.006 |  | + | - | + |
| 21 | Germany | 0.005 | 0.009 | 0.000 | 0.016 | 0.111 | 0.003 | 0.010 | 0.005 | 0.006 | 0.012 | 0.016 | 0.009 | 0.003 | 0.001 | 0.001 | 0.006 | 0.009 | 0.004 | 0.030 | 0.010 |  | - | + |
| 22 | Sweden | 0.009 | 0.000 | 0.000 | 0.004 | 0.087 | 0.000 | 0.004 | 0.000 | 0.002 | 0.006 | 0.007 | 0.003 | 0.000 | 0.000 | 0.000 | 0.000 | 0.002 | 0.007 | 0.016 | 0.004 | 0.003 |  | + |
| 23 | Finland | 0.038 | 0.032 | 0.013 | 0.016 | 0.094 | 0.024 | 0.013 | 0.024 | 0.017 | 0.026 | 0.014 | 0.017 | 0.015 | 0.016 | 0.019 | 0.021 | 0.025 | 0.022 | 0.020 | 0.018 | 0.043 | 0.020 |  |
